# Cancer Stem Cells—Origins and Biomarkers: Perspectives for Targeted Personalized Therapies

**DOI:** 10.3389/fimmu.2020.01280

**Published:** 2020-08-07

**Authors:** Lia Walcher, Ann-Kathrin Kistenmacher, Huizhen Suo, Reni Kitte, Sarah Dluczek, Alexander Strauß, André-René Blaudszun, Tetyana Yevsa, Stephan Fricke, Uta Kossatz-Boehlert

**Affiliations:** ^1^Department of Immunology, Fraunhofer Institute for Cell Therapy and Immunology, Leipzig, Germany; ^2^Department of Gastroenterology, Hepatology and Endocrinology, Hannover Medical School, Hannover, Germany

**Keywords:** cancer stem cells, senescence, targeted therapy, CAR cells, biomarkers, precision therapy

## Abstract

The use of biomarkers in diagnosis, therapy and prognosis has gained increasing interest over the last decades. In particular, the analysis of biomarkers in cancer patients within the pre- and post-therapeutic period is required to identify several types of cells, which carry a risk for a disease progression and subsequent post-therapeutic relapse. Cancer stem cells (CSCs) are a subpopulation of tumor cells that can drive tumor initiation and can cause relapses. At the time point of tumor initiation, CSCs originate from either differentiated cells or adult tissue resident stem cells. Due to their importance, several biomarkers that characterize CSCs have been identified and correlated to diagnosis, therapy and prognosis. However, CSCs have been shown to display a high plasticity, which changes their phenotypic and functional appearance. Such changes are induced by chemo- and radiotherapeutics as well as senescent tumor cells, which cause alterations in the tumor microenvironment. Induction of senescence causes tumor shrinkage by modulating an anti-tumorigenic environment in which tumor cells undergo growth arrest and immune cells are attracted. Besides these positive effects after therapy, senescence can also have negative effects displayed post-therapeutically. These unfavorable effects can directly promote cancer stemness by increasing CSC plasticity phenotypes, by activating stemness pathways in non-CSCs, as well as by promoting senescence escape and subsequent activation of stemness pathways. At the end, all these effects can lead to tumor relapse and metastasis. This review provides an overview of the most frequently used CSC markers and their implementation as biomarkers by focussing on deadliest solid (lung, stomach, liver, breast and colorectal cancers) and hematological (acute myeloid leukemia, chronic myeloid leukemia) cancers. Furthermore, it gives examples on how the CSC markers might be influenced by therapeutics, such as chemo- and radiotherapy, and the tumor microenvironment. It points out, that it is crucial to identify and monitor residual CSCs, senescent tumor cells, and the pro-tumorigenic senescence-associated secretory phenotype in a therapy follow-up using specific biomarkers. As a future perspective, a targeted immune-mediated strategy using chimeric antigen receptor based approaches for the removal of remaining chemotherapy-resistant cells as well as CSCs in a personalized therapeutic approach are discussed.

## Introduction

In 2018, according to the GLOBOCAN study, the malignant neoplasms with the highest mortality were lung (1.76 million deaths), stomach (783,000 deaths), liver (782,000 deaths), breast (627,000 deaths), and colorectal cancers (551,000 deaths) as well as blood cancers including leukemia (309,000 deaths) (1). All of these cancers are heterogeneous tumors containing cells with various stem cell properties, as described below. Already in 1877, Virchow's student Cohnheim noticed this cell population and pointed out that it possesses an embryonic character (2). Today, those cells are called cancer stem cells (CSCs) or tumor-initiating cells (TICs) and are seen as drivers of tumor establishment and growth (2–5), often correlated to aggressive, heterogeneous and therapy-resistant tumors (6, 7). Upon application of therapeutic regimens such as chemo- or radiotherapy the composition of tumor cell subpopulations changes (6, 8). At first, tumor cells with a high proliferative capacity are targeted and depleted causing a decrease in tumor size while CSCs survive (9). Additionally, some tumor cells will become senescent [therapy-induced senescence (TIS)], and subsequently can cause a change in the tumor microenvironment (TME) with tumor promoting effects due to the senescence-associated secretory phenotype (SASP) (6, 10–12).

It is well-known that CSCs are resistant to treatment and can cause tumor relapses (13). However, under the therapeutic pressure and changed microenvironment CSCs can be newly generated. In this case, these cells do originate from non-CSCs or from therapy-induced senescent tumor cells (14–18). It is therefore of importance to characterize these cells in detail and to understand their origin at the time of tumor initiation and tumor relapse.

This review underlines the role for a thorough investigation of tumors especially in the post-therapeutic period. Such post-therapeutic or therapy follow-up diagnostics are not conducted in the clinic on a regular basis, yet. The importance of specific biomarkers that analyze several parameters, such as CSCs phenotypes, senescence and TME composition, will allow the detection of therapy-resistant CSCs that cause tumor recurrence. A precise elimination of those cells of risk in a timely fashion using targeted cellular therapeutic approaches as the second line therapy is discussed in this study.

## CSCs and Their Origin at Tumor Initiation

**Figure 1:**
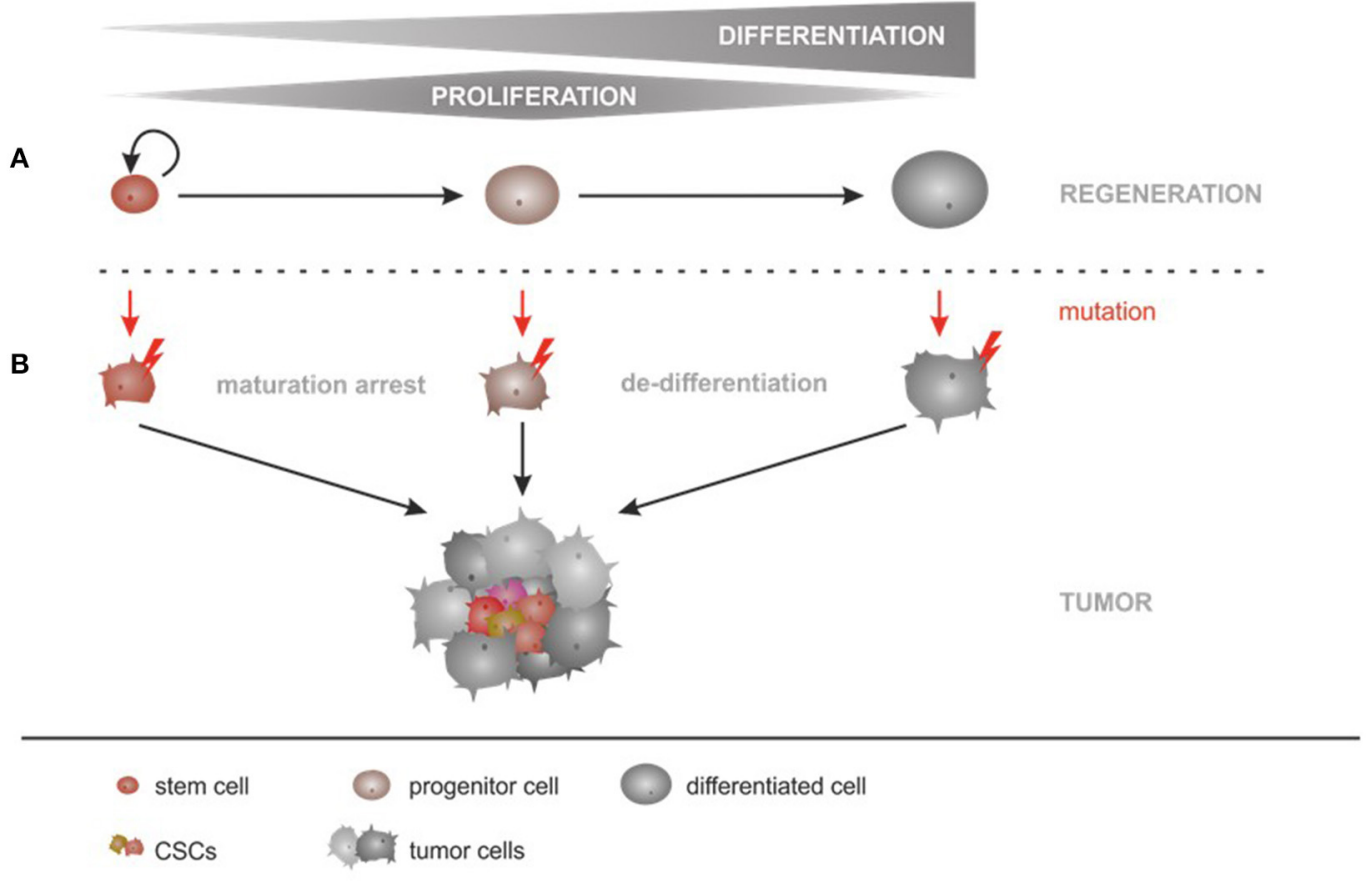
The origin of CSCs at tumor initiation: The two hypotheses of CSC generation. **(A)** The proliferation and differentiation of adult tissue resident stem cells is part of the physiological regeneration program that maintains tissue homeostasis. Adult tissue resident stem cells divide asymmetrically and generate transient amplifying cells, which possess a high proliferative capacity. These cells terminally differentiate; a process during which they will lose their proliferative capacity to finally support organ homeostasis. **(B)** Tumors can be generated by step-wise accumulation of several mutations (red lightening) that transform differentiated cells and cause a de-differentiation. Tissue resident stem cells as well as their progeny can accumulate mutations that lead to uncontrolled and niche independent growth. Heterogeneous tumors are generated. CSCs share phenotypic characteristics and several markers have been described in solid as well as in liquid cancers.

Tumor initiation can either be driven by transformed differentiated cells or transformed tissue resident stem cells (19) (compare Figure 1). The transformation can take place during tissue regeneration and can additionally, be initiated and/or accelerated as a response to infections, toxins, radiation or metabolic influences causing mutations (20, 21). During the transformation process, oncogenes are overexpressed and tumor suppressors are inactivated promoting uncontrolled growth of the cells (19). As a consequence, cells de-differentiate and acquire stem cell characteristics (19). The transformation of tissue resident stem cells or their progeny is believed to presuppose a different set of genomic changes allowing uncontrolled, niche-independent proliferation (5, 22). As stem cells already possess unlimited growth potential, it is believed that the transformation of stem cells and their progeny requires only few genomic changes (5, 22, 23). For example, the low mutagenic changes, identified in more than 10% of gastric cancers suggest that these tumors arise from tissue resident stem cell populations (24). Two stem cell populations have been identified in gastric cancers: slow cycling cells expressing the transcription factor Mist1 in the gastric corpus and Leucine-rich repeat-containing G-protein coupled receptor 5 (Lgr5)-expressing cells in the gastric antrum (25–27). Both populations have been linked to cancer generation in mouse models (24, 26, 27). In colon cancers, recent studies in mice have shown that even differentiated intestinal epithelial cells can be potential CSCs (28). The fact that adult differentiated cells, tissue resident stem cells or their progeny can promote tumor generation has also been shown in the liver. Cell tracking, *in vitro* and *in vivo* studies showed that liver cancer can originate from adult hepatocytes (29–32) as well as from hepatoblasts and hepatic progenitors (31, 32).

Tumor type, prognosis and aggressiveness are also influenced by the origin of the tumor, as analyzed for instance in breast cancers (33–35). Breast tumors originating from luminal progenitors are associated with a good prognosis, except those overexpressing Her2 (34, 36). Tumors originating from basal-like progenitors show a very aggressive phenotype (34).

In squamous cell carcinomas the differentiation phenotype seems to be influenced by the cell of origin and the kind of driver mutation, both responsible for the invasiveness and aggressiveness of the tumor (37, 38). Loss of the phosphatase and tensin homolog (Pten) as well as the liver kinase B1 (Lkb1) in lung epithelia causes tumor formation of highly penetrant tumors. These tumors are rarely metastatic and are characterized by a differentiated phenotype (37). Basal cells located within the trachea and main bronchi have been shown to self-renew and to form heterogeneous spheres (39). These basal stem cells can cause basal cell hyperplasia or epithelial hypoplasia, finally resulting in squamous cell metaplasia or dysplasia, which are discussed as precursors of squamous cell lung carcinomas (SCC) (39, 40). Studies by Fukui et al. suggest that high basal cell signatures correlate to a clinically aggressive phenotype in lung adenocarcinoma (40). Adenocarcinomas are considered to originate from sub-segmental airways of the bronchioalveolar stem cells or Type I and Type II pneumocytes (39). These bronchioalveolar stem cells are quiescent in healthy lungs but can enter proliferation cycles and could be targets of mutations causing transformation (39, 41). In mouse models, data indicate that small cell lung cancers (SCLC) can also originate from other cell types, i.e., neuroendocrine cells (42).

While in solid tumors the origin is heavily discussed, in hematological tumors the situation seems to be clearer. In acute myeloid leukemia (AML), the cell of origin is thought to be a hematopoietic stem or progenitor cell (43). However, a subgroup of human AML has been shown to share expression profiles with lymphoid T-cell progenitors. The authors showed that under oncogenic conditions, DN2 (double negative 2) T-cell progenitors process into lymphoid, biphenotypic, and myeloid leukemia cells (43–45). In chronic myeloid leukemia (CML), the cell of origin is characterized by the expression of the Bcr-Abl oncogene, generated from a chromosomal translocation between chromosome 9 and 22 (46, 47). This molecular aberration defines the chronic phase in CML, which progresses into blast crisis upon additional mutations that promote self-renewal (46, 47). While leukemic stem cells (LSCs) are well-defined and characterized in AML and CML, the concept of CSCs in acute lymphoblastic leukemia (ALL) and also in non-hodgkin lymphoma (NHL) is less established (48–50).

Tumors generated on the basis of CSCs are believed to follow a unidirectional hierarchy, in which only the CSC population is able to initiate tumor growth (51). At the time point of tumor initiation, it is suggested that cancer stem cells divide asymmetrically to maintain the CSC pool (52). These asymmetric divisions generate transient amplifying cells, which are undergoing symmetric divisions; therefore having a high proliferative capacity (51, 52). Based on recent data from hematological cancers (AML), the hierarchical model proposed by Bonnet and Dick (43) is most likely a simplified description. It is now believed that the organization of CSCs (in solid as well as in hematological cancers) is more complex (52–56). In contrast to the CSC model in which only a small subpopulation of cells is able to promote tumor initiation and growth, the clonal evolution model states that genetically unstable cells accumulate genomic and genetic alterations over time causing an increase in tumor aggressiveness, resistance and heterogeneity (5, 57). Both models are not mutually exclusive, which can be explained by the cellular plasticity (plasticity model) that suggests, that different cellular states can interconvert (as explained later) (5, 57).

Because CSCs have been shown to cause tumor initiation and tumor relapses, the search for biomarkers that characterize these cells and allow therapeutic as well as prognostic prediction or follow-up is ongoing. The most prominent markers of solid and hematological tumors are described in the following section.

### Biomarkers for CSC Populations in Solid Cancers

In solid cancers, the clinical use of CSC specific biomarkers is very limited, besides the use of the carcinoembryonic antigen (CAE), fragments of the cytokeratin 19 (YFRA 21-1) (58) and the alpha-fetoprotein (AFP) that is expressed by cancer stem cells (58, 59). Importantly, most markers expressed in CSCs can also be found in adult tissue resident stem cell populations, human embryonic stem cells (hESC) or adult tissues (60). Additionally, most markers label heterogeneous stem cell populations pointing to the fact that their characterization and isolation has to be based on marker combinations using several surface markers or combinations of extracellular as well as intracellular markers; to identify and isolate cells that promote tumor initiation, resistance and relapse.

Below, a short summary of the most prominent markers is provided. CSC markers that could have potential usefulness within therapeutic, diagnostic, and prognostic approaches are pointed out (compare Tables 1–7) and focus on most deadliest tumors of lung, liver, breast, stomach, and colorectal as well as AML and CML. Tables 1–7 provide an extensive list of markers expressed in CSCs. A comparison shows that several markers are expressed in several tumor types.

**Table 1:** Examples of lung cancer stem cell markers and their use as diagnostic, predictive, or therapeutic biomarkers.

**Marker**	**Stem cell marker**	**Biomarker diagnostic**	**Biomarker therapeutic**	**Biomarker prognostic**
**Surface markers, CD**				
CD44 (and its variants)	(61–66) (39, 67–69)* (70)**	(71)	(71–80)	(61, 64, 70, 81) (39, 69)*
CD87	(82)			
CD90	(83) (39, 67)*			
CD133	(84–99) (39, 67–69)* (70, 100)**		(74, 101–104) (69)*	(91, 105–112) (39, 67, 68)* (70)**
CD166	(62, 66, 113) (39, 68)*			(113)
**Surface markers, not CD**				
EpCAM	(62, 66, 86, 114, 115)	(116–120)	(121)	(117, 122–124)
**Intracellular markers**				
ALDH	(65, 84, 114, 125–129) (39, 68, 69, 130)*	(131)	(132–134)	(62, 128, 135) (39, 69, 130)* (70, 126)**
Nanog	(70)			(70, 126) (69)*
Oct-3/4	(96) (67, 69)*		(67)*	(136) (69)*

The table lists examples of cancer stem cell markers and indicates those which have been tested as biomarkers within a therapeutic (metastasis, tumor stage, size), diagnostic, or prognostic (survival, resistance etc.) approach. Starsindicate reviews (*) and contradictory results (**).

**Table 2:** Examples of breast cancer stem cell markers and their use as diagnostic, predictive, or therapeutic biomarkers.

**Marker**	**Stem cell marker**	**Biomarker diagnostic**	**Biomarker therapeutic**	**Biomarker prognostic**
**Surface markers, CD**				
CD24	(137)			
CD29 (ß1 integrin)	(137, 138)			
CD44 (and its variants)	(139–149)	(150–154)	(76, 150, 152, 154–166)	(166–171) (172, 173)**
CD49f	(174–176) (177)*		(178)	(175, 178, 179)
CD61	(137, 180)			
CD70				(181)
CD90	(182)			
CD133	(183) (184)*	(185–187)	(188–190) (184)*	(191–193) (184)*
**Surface markers, not CD**				
CXCR4	(194)			
EpCAM		(186)	(186)	
LGR5	(195)			(195)
ProC-R	(196)			
**Intracellular markers**				
ALDH	(147, 148, 197, 198) (199, 200)*		(198, 201, 202) (199)*	(171, 192, 197, 203–208) (200)* (209, 210)**
BMI-1			(143, 211–218) (219)*	
Nanog			(142)	(220, 221)
Notch	(222–224)	(222, 225)	(187, 212, 222, 224, 226–230)	(222, 226, 231–234) (235)*
Oct-3/4		(142)		(220, 221)
Sox2		(142)		
**Signaling pathways**				
Wnt/ß-Catenin	(195, 236, 237)	(236)	(237)	

The table lists examples of cancer stem cell markers and indicates those which have been tested as biomarkers within a therapeutic (metastasis, tumor stage, size), diagnostic, or prognostic (survival, resistance etc.) approach. Stars indicate reviews (*) and contradictory results (**).

**Table 3:** Examples of gastric cancer stem cell markers and their use as diagnostic, predictive, or therapeutic biomarkers.

**Marker**	**Stem cell marker**	**Biomarker diagnostic**	**Biomarker therapeutic**	**Biomarker prognostic**
**Surface markers, CD**				
CD24	(238) (239)*	(240)*	(241)	(242–244) (239, 245)*
CD44 (and its variants)	(246–251) (239, 240, 245, 252)*	(247, 251, 253, 254) (240)*	(255–257) (239, 240, 245)*	(247, 251, 254, 258–260) (239, 240, 245, 252)*
CD90	(251) (239, 245)*			
CD133	(247, 249, 250) (239, 240, 252)*	(254, 261) (240)*	(257) (239, 240)*	(254, 262–265) (239, 240, 252)*
**Surface markers, no CD**				
CXCR4	(266)		(267)*	(268–271)
EpCAM	(248, 249, 272) (239, 240, 252)*		(273)	(265, 272)
LGR5	(274) (252)*	(240)*	(275, 276) (252)*	(275, 277–279)
LINGO2	(280)			(280)
**Intracellular markers**				
ALDH	(249, 281, 282) (239, 240, 252)*			(260, 281, 282)
Letm1	(283)			(283)
Musashi2	(284)			(284)
Nanog	(285) (239, 286)*	(287) (240)*		(287, 288) (286)*
Oct-3/4	(239, 252)* (289)**			(247, 265, 288) (289)**
Sox2	(247) (239, 240, 252, 290)*	(240)* (291)**	(292)	(247, 288, 293) (265)**

The table lists examples of cancer stem cell markers and indicates those which have been tested as biomarkers within a therapeutic (metastasis, tumor stage, size, resistance), diagnostic (i.e., resistance), or prognostic (survival, resistance etc.) approach. Stars indicate reviews (*) and contradictory results (**).

**Table 4:** Examples of liver cancer stem cell markers and their use as diagnostic, predictive, or therapeutic biomarkers.

**Marker**	**Stem cell marker**	**Biomarker diagnostic**	**Biomarker therapeutic**	**Biomarker prognostic**
**Surface markers, CD**				
CD24	(294–296) (297, 298)*		(298)*	(295)
CD44	(299, 300) (298)*			(300–303) (298)* (304)**
CD90	(295, 300, 305–308) (297, 298)*			(295, 300, 304, 309) (298)*
CD133	(295, 296, 300, 310–313), (297, 298)*	(314)		(295, 300, 304, 311, 314–319), (320)**, (298)*
**Surface markers, not CD**				
EpCAM	(297, 298)* (294, 300, 304, 311, 321)	(322)	(298)*	(300, 301, 304, 311, 319, 321–327) (298)*
**Intracellular markers and pathways**				
AFP	(311, 321)		(328)	(311, 321, 329), (330)*
Nanog	(312, 313, 331), (298)*		(298)*	(331) (298)*
Notch	(295, 296, 305)		(295)	
Oct-3/4	(313, 331), (298)*			(309, 331), (298)*
Sox2	(313) (298)*			
Wnt/ ß-catenin	(295, 313)		(295)	(313) (330)*, **

The table lists examples of cancer stem cell markers and indicates those which have been tested as biomarkers within a therapeutic (metastasis, tumor stage, size, resistance), diagnostic (i.e., resistance), or prognostic (survival, resistance etc.) approach. Stars indicate reviews (*) and contradictory results (**).

**Table 5:** Examples of colorectal cancer stem cell markers and their use as diagnostic, predictive, or therapeutic biomarkers.

**Marker**	**Stem cell marker**	**Biomarker diagnostic**	**Biomarker therapeutic**	**Biomarker prognostic**
**Surface markers, CD**				
CD24	(332)			
CD44	(333–335) (336)*		(337, 338)	(339)
CD133	(334, 340) (336)*	(340)	(338, 341–343)	(340, 344)
CD166	(333) (336)*			(333)
**Surface markers, not CD**				
EpCAM	(335) (336)*		(345, 346) (347)*	
LGR5	(335, 348–350) (336)*	(351)	(352)	(353, 354)
**Intracellular markers**				
ALDH	(335, 355, 356) (336)*			(355) (357)*
Letm1	(358)			(358)
Nanog	(359, 360) (336)*		(361)	(361, 362)
Oct-3/4	(363, 364) (336)*			(363, 365)
Sall4		(366)		(366)
Sox2	(359, 367, 368) (336)*			(367–369)

The table lists examples of cancer stem cell markers and indicates those which have been tested as biomarkers within a therapeutic (metastasis, tumor stage, size, resistance), diagnostic (i.e., resistance), or prognostic (survival, resistance etc.) approach. Starsindicate reviews (*).

**Table 6:** Examples of AML cancer stem cell markers and their use as diagnostic, predictive, or therapeutic biomarkers.

**Marker**	**Stem cell marker**	**Biomarker diagnostic**	**Biomarker therapeutic**	**Biomarker prognostic**
**Surface markers, CD**				
CD33		(370)	(371–392)	(393)
CD123	(370, 394–396)	(395, 397–399)	(373–376, 397, 400–412)	(394, 399, 403, 413)
**Surface markers, not CD**				
CLL-1	(414–416)	(370)	(414, 417–419)	(415, 420)
TIM3	(421)		(422)	(420, 423)
**Intracellular markers**				
ALDH	(424)			(424, 425)
Nanog	(426)	(427)		(426)
Oct-3/4	(428)	(429)		(429–431)
Sox2				(431, 432)

*The table lists examples of cancer stem cell markers and indicates those which have been tested as biomarkers within a therapeutic (metastasis, tumor stage, size, resistance), diagnostic (i.e., resistance), or prognostic (survival, resistance etc.) approach*.

**Table 7:** Examples of CML cancer stem cell markers and their use as diagnostic, predictive, or therapeutic biomarkers.

**Marker**	**Stem cell marker**	**Biomarker diagnostic**	**Biomarker therapeutic**	**Biomarker prognostic**
**Surface markers, CD**				
CD25	(433–437) (438–440)*	(439)*	(441)	
CD26	(433–437, 442–445) (438–440)*	(443, 446) (439)*	(434, 447, 448)	(443)
CD33	(433, 434) (438–440)*			
CD36	(434, 435) (438)*		(435)	
CD117	(433, 434, 437) (439, 440)*			
CD123	(434, 449–451) (439, 440)*		(449, 450)	
**Surface markers, not CD**				
IL1RAP	(433–437, 452, 453) (438–440)*	(439)*	(452, 453)	(437)
**Intracellular markers**				
JAK/STAT	(433) (438)*			
Wnt/β-catenin	(454–456) (438, 457)*		(454, 458, 459) (457)*	
FOXO	(460) (438)*		(460)	
Hedgehog/Smo/Gli2	(461) (438)*		(461)	

The table lists examples of cancer stem cell markers and indicates those which have been tested as biomarkers within a therapeutic (metastasis, tumor stage, size, resistance), diagnostic (i.e., resistance), or prognostic (survival, resistance etc.) approach. Stars indicate reviews (*).

#### CD44

CD44 is a biomarker which is not only expressed in solid but also in hematological cancers (see below). Its expression is associated with increased proliferation, self-renewal and metastasis (61, 149, 462, 463). For example, in colorectal cancers, expression of CD44/CD166 characterizes a cell population able to form tumor spheres, suggesting anchorage-independent proliferation of these cells (333). In other studies, CD44^high^/CD133^high^ cells showed increased tumorigenic capabilities (334). Also in breast cancers, the percentage of CD44^+^/CD24^**−**^**/**CK^+^/CD45^−^ cells was shown to be increased in malignant lesions compared to non-malignant lesions (139). A significant decrease in proliferation and migration of breast cancer cells was observed after the knock-down of CD44 (140). In gastric cancers, the knock-down of CD44 reduced sphere formation and caused decreased tumor growth in severe combined immunodeficiency mice (246). In many tumors (e.g., breast and liver), CD44 is expressed as isoform and its expression has been associated with increased cancer stem cell properties (141). In lung cancers, CD44v9 expression correlates significantly with early-stage lung adenocarcinoma and epidermal growth factor receptor (EGFR) mutations (464). Variants of CD44 are also expressed in gastric cancers and promote tumor initiation (248).

The CSC marker CD44 has been indicated as a biomarker for diagnostic, therapeutic, and prognostic approaches (compare Tables 1–5). In gastric cancer patients, CD44^+^ circulating tumor cells correlated with a poor prognosis (465). In colorectal cancers, a prognostic quantitative real-time PCR was established to analyze the expression of CD44v2 showing that a high expression correlated with a worse prognosis (339). In gastric cancers, the expression of CD44 and CD90 correlated with distant metastasis and could therefore be used as a diagnostic biomarker (251) and was suggested as a biomarker for treatment response (253). Therapeutic approaches targeting CD44 have been made using e.g. adenoviral delivery of siRNA *in vitro* (337). Furthermore, CD44-targeting drug conjugated aptamers (76) or hyaluronic acid coated onto nanoparticles have been in the focus of research (155). Antibody-based photosensitizer conjugates for combined fluorescent detection and photo-immunotherapy (PIT) of CD44-expressing cells in triple-negative breast cancers (TNBC) (150) or other antibody-based approaches tested in safety studies (466–468).

#### CD133

The biomarker CD133 (Prominin-1) is expressed on hESCs and rarely found on normal tissue cells (60). The marker has been additionally identified in tumors of breast, liver, stomach, and colon (compare Tables 1–5) and has also been described as a marker that characterizes cells with high tumorigenicity and a high ability to form spheroids (184, 469). In breast cancers, its expression correlates with N-cadherin expression that was found to be significantly higher in patients with metastasis (191). In lung cancers, the expression of CD133 has been correlated to epithelial to mesenchymal transitions (EMT), in combination with other additional stem cell markers, such as BMI1 (84).

The expression of CD44 and CD133 in colorectal cancers can predict metastasis (470), however, no correlation to patient outcome could be detected (471). In breast cancers, CD133 mRNA was suggested to be suitable for prognosis prediction (193, 472) and CD133 protein has been correlated to a poor prognosis (193). Pre-clinical therapeutic approaches cover antibody-based targeting of colorectal (341, 342) as well as breast cancers (188) (compare Tables 1–5).

#### EpCAM

The epithelial cell adhesion molecule (EpCAM, CD326) is expressed on CSCs in various tumor types including colon and hepatocellular cancers (473–476). Furthermore, it is expressed in non-transformed tissues such as epithelial cells (476), and various stem and progenitor cells (477, 478). EpCAM is involved in proliferation and differentiation as well as in cell signaling and formation and maintenance of organ morphology (479). In cancer tissue, EpCAM is homogeneously expressed on the cell surface, while in epithelia it is expressed on the basolateral side (476).

In breast cancers, the expression of EpCAM is correlated to CSC-like phenotypes that promote formation of bone metastases in mice (480). In lung cancers, the expression of EpCAM is often associated with the expression of CD44 and CD166. Triple positive cells show increased clonogenicity, spheroid formation, self-renewal capacity, and show increased resistance to both 5-fluouracil and cisplatin (62).

As one of the first CSC markers, EpCAM has been evaluated as a therapeutic biomarker (compare Tables 1–5). Targeting EpCAM with different antibody formats has been performed in colorectal as well as breast cancers (347). In colorectal cancers, a therapeutic approach targeting EpCAM^+^ cells with aptamers has been performed in pre-clinical conditions (345, 346).

#### Intracellular Biomarkers as Regulators of Stemness in Solid Cancers

Both embryonic and CSCs show unlimited growth, invasive capacity and are characterized by an undifferentiated cellular state (481). This feature depends on transitions between epithelial and mesenchymal states, regulated by a network of intracellular pluripotency transcription factors. As reviewed by Hadjimichael et al. and also described by others pluripotency in ESC is regulated by a core-network of transcription factors, consisting amongst others of Oct-3/4, Sox2, Nanog, Klf4, and c-MYC as well as signaling pathways such as the Jak/Stat, Wnt/ß-catenin, Hedgehog/Notch, TGF-ß as well as FGF signaling pathways (367, 482, 483). The core-pluripotency network consisting of Nanog, Oct-3/4 and Sox2 (described in detail below) activates genes of self-renewal and suppresses genes involved in differentiation (482). Pluripotency factors as well as signaling pathways have been indicated as biomarkers for CSCs as shortly described below (compare Tables 1–5). Of note, the tables do not include all biomarkers, however describe the most abundant ones reported in the literature.

#### Sox2

The transcription factor Sox2 belongs to the SRY-related HMG-box (SOX) family, and is involved in the maintenance of an undifferentiated cellular phenotype (367). Its aberrant expression in cancers often leads to increased chemotherapy resistance and asymmetric divisions, as observed in colorectal cancers (368). In those, Sox2 expression correlates with a stem cell state and with a decreased expression of the caudal-related homeobox transcription factor 2 (CDX2), which could serve as a prognostic marker for a poor prognosis (367, 368). In gastric cancers, expression of Sox2 correlates with the tumor stage as well as with a poor prognosis (247, 288). The formation of tumor spheroids *in vitro* also correlates to the overexpression of CD44 and CD133 as well as the transcription factors Sox2, Nanog and Oct-3/4 (247). However, in another study, Sox2 levels were downregulated in gastric cancers in comparison to normal tissue and high Sox2 expression correlated with decreased metastasis and a better prognosis for the patient due to increased p21 levels (293). Therefore, the oncogenic functions of Sox2 are controversially discussed in gastric cancers, in which Sox2 might also have tumor-suppressor functions. These different functions seem to depend on the cancer origin and cellular context (484).

#### Oct-3/4

Oct-3/4, also known as POU5F1, belongs to the POU homeobox gene family and is also a regulator of pluripotency in mammalian stem cell population. Oct-3/4 is upregulated in several cancers and may support the neoplastic transformation and resistance (485). In colorectal cancer cells, Oct-3/4 causes increased migration and liver metastasis (363, 486) correlating with poor survival (365). As reviewed by Prabavathy et al. Oct-3/4 expression is correlated to increased self-renewal and metastasis in lung cancer cells (67). A meta-analysis showed that Oct-3/4 expression in lung cancer was associated with poor outcomes concerning the differentiation degree, the TNM Classification of Malignant Tumors (TNM) and lymphatic metastasis (136). In hepatocellular carcinoma (HCC) Oct-3/4 expression was correlating with tumor size and recurrence (309).

#### Nanog

Nanog is a homeobox domain transcription factor widely expressed in human cancers (487). In colorectal tumors its expression was significantly increased in CD133^+^ cells, and on the basis of a univariate analysis, Nanog expression correlated linearly to liver and lymph node metastasis and the TNM stage. It might therefore be useful as a prognostic biomarker in post-operative liver metastasis (362). In breast cancer, expression of Nanog and Oct-3/4 has been correlated to a poor prognosis of the patient as well as EMT (220, 221). In HCC cell lines Nanog expression drives selfrenewal and invasion, metastasis, and drug resistance (298).

### Biomarkers for CSC Populations in Hematological Cancer

CSC biomarkers of AML and CML have been listed in Tables 6, 7. They indicate commonly used markers and point out possible functions of these markers as biomarkers in prognosis, therapy, and diagnosis. Below a short introduction of the most relevant markers is given.

#### CD44

As mentioned above, CD44 is a common marker shared by many cancers (60). In hematological cancers, CD44 expression is functionally associated with chemotherapy resistance (488, 489). The expression of CD44 in AML is significantly correlated with a poor overall survival (OS) (490). Furthermore, CD44 was shown to be significantly higher expressed in non-remission AML patients (490). A highly relevant function of CD44 for LSCs is the adhesion to the bone marrow niche (491).

#### CD123 and CD33

In hematological malignancies, such as AML, CD123 as well as CD33 have been described as the “classical” CSC markers (492, 493). CD123 is a marker expressed on LSCs (395, 397, 494), but not exclusively (395, 398). In AML patients, CD123 expression correlates to the therapy response rate (413, 495), the relapse risk (403), and a shorter disease-free period and OS (399, 413). CD123 has been associated with increased proliferation and differentiation (494, 496).

CD33 is historically, the most commonly used marker for AML stem cells, with clinical implementation of CD33 targeting, dating back to the Food and Drug Administration (FDA) approval of gemtuzumab ozogamicin (GO) in 2000 (497). CD33 is highly expressed on blasts in around 85–90% of AML patients (433, 438, 497) and also expressed at higher densities in CML (433, 438) but less on healthy hematopoietic stem cells (HSCs). These cells are additionally characterized by expression of CD25, CD26, and Interleukin-1 receptor accessory protein (IL-1RAP) and also other markers (440).

#### CLL-1

The C-type lectin-like molecule-1 (CLL-1) is a promising alternative to the “classical” LSC targets (414). The majority of AML patients shows CLL-1^**+**^ LSCs, a marker not being expressed on HSCs (370, 414–416). Compared to CD33, CLL-1 was not only more frequently and stronger expressed on LSCs, but also not or more weakly expressed on normal tissues leading to reduced off-target effects after treatment with a respective antibody-drug conjugate. Therefore, CLL-1 might be a more suitable and specific LSC target than CD33 (414). A high expression of CLL-1 is associated with poor prognosis (420) and a faster relapse (415) in AML. Interestingly, controversial observations have been made using CLL-1 as a biomarker after chemotherapy. The diagnostic value of CLL-1 is discussed controversially: while Zhang et al. showed that CLL-1 was increased after chemotherapy (371), others showed that there is no difference between CLL-1 expression at diagnosis and at relapse (415) or even detected a decreased CLL-1 expression at relapse (370). The relevance of CLL-1 as a prognostic biomarker for chemotherapy failure or relapse is therefore still unclear. Its expression is not detectablewithin the chronic phase of CML (440).

#### TIM-3

Another “non-classical” LSC biomarker is T-cell immunoglobulin and mucin 3 (TIM-3), that is highly expressed on LSCs but not expressed on healthy HSCs (498). It is correlated to a poor prognosis (420) and treatment failure (423). Stem cell properties of TIM-3^+^ cells were confirmed by engraftment in a xenograft mouse model (421).

#### Intracellular Biomarkers as Regulators of Stemness in Hematological Cancers

The core-network of pluripotency associated transcription factors as well as signaling pathways have also been analyzed in hematological cancers. Fifty AML patients have been analyzed for the expression of Sox proteins, which are overexpressed in 10–22% of the patients. The analysis showed that high levels of Sox proteins may have a prognostic value (432). The analysis of Oct-3/4 expression correlated with an unfavorable prognosis and is associated with FMS-like tyrosine kinase 3-internal tandem duplications (FLT3-ITD) (430).

Activation of stemness-associated pathways especially in CML has been shown to promote extensive proliferation and has been linked to the onset of blast crisis, which is associated with a loss of differentiation of the leukemia initiating cells. An important impact on this effect has the Wnt/ß-catenin pathway (46) that promotes HSC proliferation, independent of the bone marrow niche (5, 22, 499). Especially, resistance to the tyrosine kinase inhibitor imatinib has been shown to correlate to an increased nuclear localization of ß-catenin (454, 458, 500). Inhibitors targeting the Wnt pathway have been shown to be of advantage for example in long-term cell cultures (500). Additionally, the hedgehog pathway has been suggested to be involved in chemotherapeutic resistance in CML, which is also characteristic for chronic phase CML cells (47). Mouse studies also indicate the involvement of the hedgehog pathway (46, 47), which has been implicated as a therapeutic biomarker for CML (456, 461).

To summarize, CSCs at tumor initiation originate from either differentiated cells or adult tissue resident stem cells (5, 19, 22). Several data indicate that the origin strongly correlates to the aggressiveness of the tumor. Therefore, extra- and intracellular biomarkers that characterize CSCs have been identified and implemented to be of diagnostic and prognostic advantage. However, stem cells are subject to a high degree of plasticity modulated by the TME (19), that is significantly changed by chemo- and radiotherapies and composed of several different cell types. In the following section, focus will be laying on senescent tumor cells as part of the TME as they have long-term influence on TME and CSC development and progression.

## The Escape of Cancer Stem Cells From Therapy

**Figure 2:**
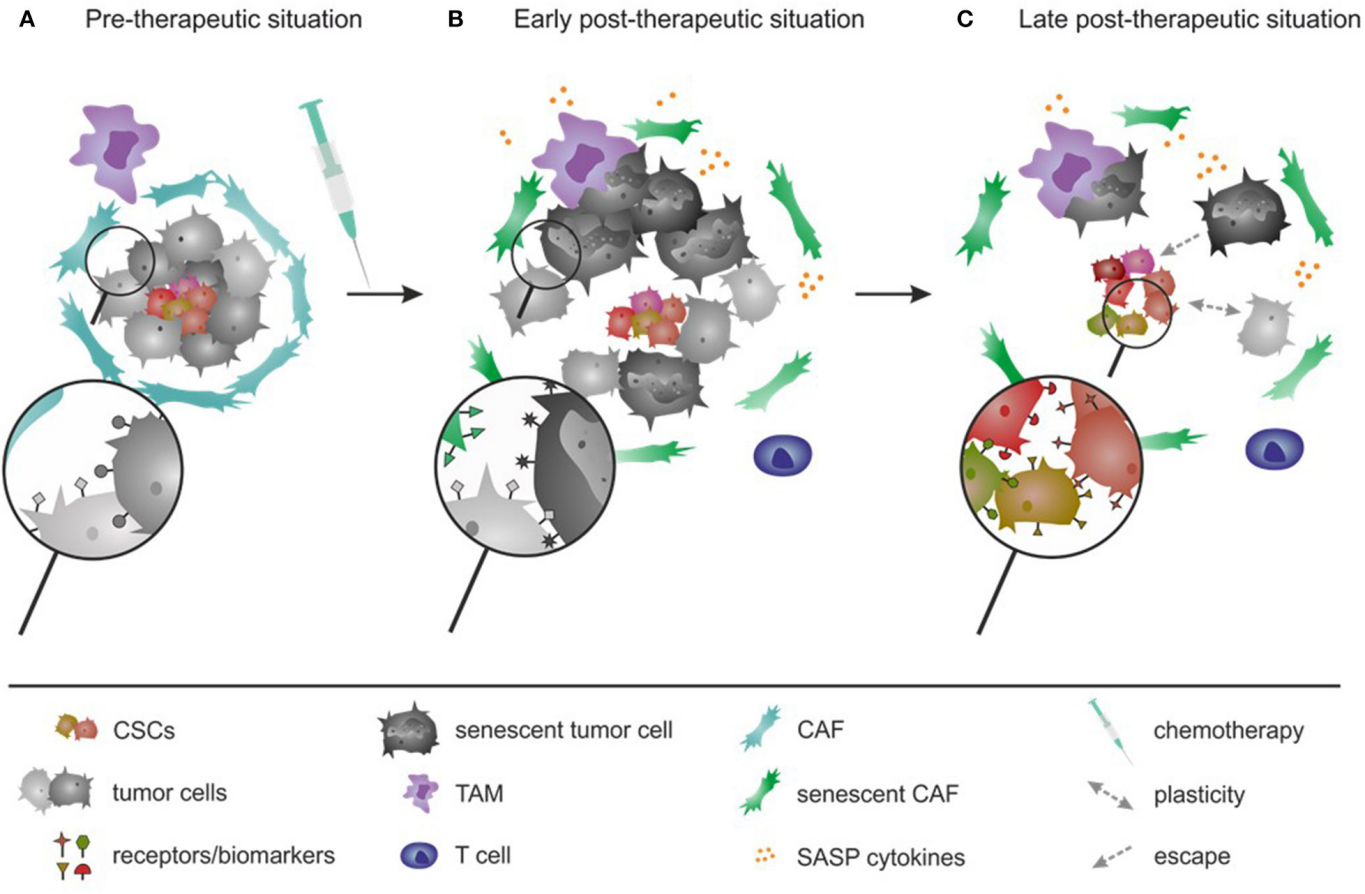
Kinetic of tumor development in pre-, early-, and late-therapeutic period upon application of chemo- and/or radiation therapy: current situation in the clinic. **(A)** In the pre-therapeutic situation, heterogeneous tumors are composed of several cell types, including CSC, tumor cells, TAMs, and CAFs; all characterized by biomarkers. **(B)** In the early post-therapeutic period, upon application of the first-line treatment (that currently uses mostly chemo- or radiotherapeutic regimens) several important changes occur in the tumor, in particular: tumor cells or CAFs die due to the therapy or become senescent, whereas CSCs mostly survive the treatment. Senescent cells (tumor cells and CAFs) attract immune cells toward the senescent site via SASP. The SASP therefore plays a positive role and attracts immune cells in this early post-therapeutic situation. Attracted immune cells promote the clearance of dead, of necrotic, and senescent tumor cells and CAFs. **(C)** In the late post-therapeutic situation uncleared senescent tumor cells and senescent CAFs and SASP thereof play a negative (pro-tumorigenic) role and support tumor development. SASP molecules provide stimulating factors for CSCs for further uncontrolled proliferation as well as their maintenance. Also, remaining senescent tumor cells acquire additional mutations that promote activation of a stemness phenotype and finally a tumor relapse.

At the moment first-line therapeutic treatments in progressed tumors include in the most cases surgery, chemo- as well as radiotherapies (501) (compare Figure 2). Those have been shown to induce DNA damage and to trigger senescence in cancer cells, a process known as therapy-induced senescence (TIS) (10, 502, 503). TIS will cause a decreased tumor size and attracts immune cells such as neutrophils, monocytes as well as T-cells toward senescent tumor site (503). However, over a long-term period the anti-tumorigenic effects of TIS are lost and the cancer might gain stemness causing tumor relapses (Figure 2).

### Therapy-Induced Senescence: Its Hallmarks, Biomarkers, and Its Role in CSC Generation

Agents that induce DNA damage such as chemo- and radiation therapies have been identified to trigger senescence in differentiated cancer cells (10). TIS has been in the research focus, because it significantly contributes to the long-term outcome of patients (12). The DNA damage response ultimately activates one or several tumor suppressors pathways [p53, p16 (Ink4a), p21 (Waf1), and retinoblastoma (RB)], that trigger and maintain the senescence growth arrest (504). However, it is important to mention that the senescence phenotype can also be induced in cancer cells which lack functional p53 and RB protein (504). TIS and senescence in general, are recognized as a double-edged sword, that on the one hand leads to the attraction of immune cells, inflammation, and elimination of senescent tumor cells and correlates with a positive post-treatment prognosis and treatment outcome (505–507). On the other hand, senescence can play a strong pro-tumorigenic role that supports CSC generation, as described below.

Senescent cells are characterized by biochemical and morphological changes such as flattening and/or nuclear enlargement (508). There are several classical biomarkers of cellular senescence and they comprise: senescence-associated beta-galactosidase (SA-ß-gal) activity, expression of p53 protein, the amount of p53 in the nucleus, increase in expression of p14 (Arf), p16 (Ink4a) and p21 (Waf1), SASP, and often senescence-associated heterochromatic foci (SAHF) (12, 505, 507, 509–515). Furthermore, senescent cells display low Ki67 levels and show levels of heterochromatin protein 1 (HP1) gamma (516), as well as di- or tri-methylated lysine 9 of histone H3 (H3K9Me2/3) and histone H2A variant (macroH2A) (505, 517, 518). The usefulness of telomere length as a biomarker of senescence has been questioned (505).

Biomarkers that underline the effect of a therapeutic approach based on the induction of senescence have to be evaluated carefully and quite often simultaneously. The investigation of senescence markers after post-operative chemotherapy in muscle-invasive bladder cancer (MIBC) revealed that the simultaneous expression of several markers involved in the p53 pathway has to be checked to correctly assess the pathological outcome of MIBC (509). The analysis revealed that the expression of p14 (Arf) was associated with an impaired response to chemotherapy and poor prognosis, whereas p21 (Waf1) expression was related to reduced tumor cell proliferation (509).

TIS can play an anti-cancerous role (503). As demonstrated in our studies in premalignant and malignant liver disease, the induction of senescence leads to a so-called “senescence surveillance” mechanism, which relies on innate and adaptive immune cells. These cells clear senescent premalignant cells, thereby protecting premalignant liver from cancer development (535, 536). Interestingly, in further studies, we could show that the chemokine (monocyte chemoattractant protein 1, MCP-1) axis is of importance for the induction and maintenance of senescence and for the sufficient immune surveillance in the liver (525). Several biomarkers of senescence were found to correlate with a disease-free survival or with an improved OS in several solid cancers (516, 524). One such indicator, a lysosomal-beta-galactosidase (GLB1) that hydrolyzes beta-galactose from glycoconjugates and represents the origin of SA-ß-gal, was reported as a reliable senescence biomarker in prostate cancer (516). Inhibition of the cyclin-dependent kinase 4/6 (CDK)-RB pathway by a novel drug, SHR6390, resulted in reducing the levels of Ser780-phosphorylated RB protein and correlated with the G1 arrest as well as with cellular senescence in a wide range of human RB^+^ tumor cells *in vitro* (520). Xiang et al. identified seven senescence-associated genes (SAGs, Table 8) significantly decreased in senescent cells and increased in HCC tissues (524). Interestingly, those SAGs were strongly associated with OS, especially in Asian populations, and had a better predictive accuracy in comparison to serum AFP in predicting OS of HCC patients (524). Recently, Smolle et al. reviewed and underlined the role of members of the inhibition of growth (ING) family. These act as tumor suppressors, regulating cell cycle, apoptosis, and cellular senescence. The authors proposed them as clinically useful biomarkers in the detection and prognosis of lung cancer (523).

**Table 8:** Biomarkers of therapy-induced senescence (TIS).

**Biomarker**	**References**
Senescence-associated beta-galactosidase (SA-β-Gal)	(12, 14, 504, 505, 510, 516–520)
P53	(14, 504, 520, 521) (507)*
Retinoblastoma (RB) Protein (CDKN2A; Ser780phosphorylated RB protein; cyclin-dependent kinase (CDK) 4/6-retinoblastoma)	(12, 14, 504, 519–521) (507)*
P14 (human) P19 (mouse)	(12, 509, 510, 514, 515, 519) (505, 507, 513)*
P16 (INK4A; CDKN2)	(12, 14, 509, 512, 515, 519, 522) (505, 507, 513, 514, 516)
P21 (WAF1)	(14, 509, 522) (505, 507, 513)*
Senescence-associated heterochromatic foci (SAHF)	(12, 509, 510, 515, 519) (505, 507, 513)
Heterochromatin protein 1 (HP1) gamma	(509, 516, 518)
Telomere length	(505)*
Di- or tri-methylated lysine 9 of histone H3 (H3K9Me2/3)	(505, 517, 518)
Histone H2A variant (macroH2A)	(505, 517, 518)
Lysosomal-beta-galactosidase (GLB1)	(516)
Inhibition of growth (ING) family of proteins (ING−1,−2,−3,−4,−5)	(523)
Senescence-associated genes (SAGs) family: [18B (KIF18B), Citron kinase (CIT), Centrosomal protein 55 (CEP55), minichromosome maintenance complex component 5/7 (MCM), Cell division cycle 45 (CDC45), enhancer of zeste homolog 2 (EZH2)]	(524)
Senescence-associated secretory phenotype (SASP)	(12, 14, 510, 519) (505, 507, 509, 522)
Soluble TNF-receptor-II	(11, 523)
Chemokine (C-C motif) receptor/ligand 2, (CCR2/CCL2); Monocyte chemoattractant protein 1 (MCP-1) axis	(525)
IL-1	(526)
IL-6	(527–531)
IL-8	(528, 531, 532) (526, 527)
Regulated on activation, normal T cell expressed and secreted (RANTES)	(533, 534)

Examples of the most important biomarkers of TIS are listed. Stars indicate reviews (*).

In line with the positive role of senescence, evidence exists regarding the role of TIS in turning “cold” tumors toward a “hot” phenotype that results in activating immune responses against tumor antigens (503). As reported in Her2^+^ breast cancer patients treated with Trastuzumab and chemotherapy, the treatment-induced epitope spreading was characterized by increased antibody responses not only to the tumor antigen Her2, but also to endogenous CEA, insulin-like growth factor-binding protein 2 (IGFBP2), and p53 (521).

TIS is a very important protective mechanism that is induced immediately after chemo- or radiation therapy. TIS mediates the recognition and clearance of senescent tumor cells by immune cells (503, 535). Induction of TIS after the therapy is associated with a better prognosis and OS (524). However, if senescent tumor cells are not cleared in a timely fashion, their role at a later time points shifts from positive to negative, as discussed in the section below.

### Negative Role of TIS: Cancer Progression

Several studies report a pro-tumorigenic effect of TIS leading to cancer recurrence and support of tumor development (503). Uncleared senescent cells acquire additional mutations, thereby escaping the cell cycle arrest and transform into malignant cells (536). Moreover, factors secreted by senescent cells are also reported to play a strong tumor-promoting role (526).

Was et al. suggested that senescent human colon cancer cells (HCT116) that appear during a doxorubicin-based therapy enter a “dormant” cellular state, survive the treatment, and cause tumor re-growth (537). Importantly, the recent findings by Scuric et al. suggest a long-term effect of chemotherapy and/or radiation exposure upon TIS (11). In this study, markers of cellular senescence, including higher DNA damage and lower telomerase activity, were observed many years later in breast cancer survivors (11). Elevated levels of a soluble tumor necrosis factor (TNF)-receptor-II, a pro-inflammatory biomarker and one of the main SASP molecules, were also detected (11). A negative effect of SASP was correlated to a p53 single-nucleotide polymorphism (SNP) at codon 72 which is correlated to increased risk of breast cancers (538). Using a humanized mouse model, Gunaratna et al. showed that SASP caused an increased invasion of pro-inflammatory macrophages (522). However, the inflammation proceeded into a chronic inflammation with pro-tumorigenic action and tumor-associated macrophages (TAMs) contributed to angiogenesis and increased tumor growth rates (522). Also, senescent cancer-associated fibroblasts (CAFs) and, in particular, expression of Caveolin-1 (CAV1) promote tumor invasion in pancreatic cancer (539). Moreover, in clinicopathological characteristics of patients, a high CAV1 expression directly correlates with higher levels of serum tumor antigens, with the rate of advanced tumor stage, and with significantly worse outcomes in both overall and disease-free survival (539).

It has been suggested that cancer therapies, especially chemo- and radiotherapies, possess long- and late-term pro-tumorigenic side effects and could therefore contribute to the relapse of the malignant disease they were intended to treat (540). Such long-term effects could be caused by the decreased removal of senescent cells, as described below.

#### Cancer Stemness: Senescence Escape

As mentioned above, cells undergoing senescence can still escape the senescence program and become malignant while acquiring additional mutations (519, 535, 536) (Figure 2). In our studies, we observed a spontaneous mutation [a deficiency in p19 (Arf)] in Ras-expressing hepatocytes, which resulted in a full-blown HCC development using a Ras-induced precancerous liver disease model (535, 536). The reversibility of TIS can be caused through the inactivation of tumor suppressors p53, p16 (Ink4A), p19 (Ink4d), and/or RB (504, 507, 519). Additionally, the over-expression of CDC2/CDK1 and survivin can promote cancer stem cell survival and can also promote the development of polyploidy (507). In general, mutations in CDKN2A, coding for p16 (Ink4a, CDKN2A), p21 (Waf1, CDKN1A), and p27 (Kip1, CDKN1B) as well as E2F3 and EZH2, and a high c-MYC expression might result in low percentages of senescent cells (504, 519). Moreover, particular mutations completely protect melanoma cells from cell cycle arrest upon chemotherapy: DMBC29 melanoma cells that carried a EZH2^S412C^ mutation, expressed c-MYC at a low level and a wild type of CDKN2A did not undergo senescence, in contrast to many melanoma cells treated with vemurafenib and trametinib (519).

An escape of cells from senescence was also identified by Milanovic et al. in B-cell lymphoma studies (14). In those studies, the researchers showed that senescent cells substantially upregulated an adult tissue stem cell signature and activated Wnt signaling (14). This senescence-associated stemness was an unexpected cell-autonomous phenotype that caused the generation of cells with a higher tumorigenic potential *in vitro* (14).

However, escape from senescence is not the only pathway that promotes an increase in the cancer stemness phenotype. Stemness within the tumor tissue is also regulated indirectly by signaling molecules which support the maintenance of stemness in CSCs as well as non-CSCs, as described in the following sections.

#### Cancer Stemness: SASP and CSC Maintenance

The stemness phenotype within a tumor can also be mediated via SASP (526). Several studies address the strong pro-tumorigenic phenotype (526) whose cytokines can mediate the maintenance of CSCs. The most prominent interleukins (IL) of SASP are IL-1,−6, and−8 (526). These cytokines can influence the CSC phenotype and functionality and therefore influence the plasticity phenotype of CSCs.

Using breast cancer cell lines, Di et al. showed that an induction of senescence in mesenchymal stem cells by hydrogen peroxide treatment causes an increased secretion of the inflammatory cytokine IL-6, which led to a higher migratory capacity of breast cancer cells *in vitro* as well as in xenotransplants (541). An increase in the aggressive metastatic chemoresistant phenotype upon inflammatory cytokine stimulation such as IL-1ß, IL-6, and RANTES (regulated on activation, normal T cell expressed, and secreted) was also observed by others (533, 534). Our own work indicated that IL-8 blocks differentiation of hepatocellular premalignant cells, a pathway mediated via mammalian target of rapamycin complex 1 (mTORC1) kinase, that causes an increase in chemotherapy resistance (532). An increase in tumorigenicity and EMT of breast cancer cells has been shown to correlate to an increased expression of CD44 or CSC-like properties and be caused by the senescence-associated IL-8 and IL-6 (527–529). Pathways that might be involved in such cellular reprogramming processes are the JAK2/STAT3 signaling pathway (542), the IL-6/STAT3 and NOTCH cross-talk signaling (187, 530) as well NFκB-IL-6 signaling axis, responsible for the generation of CSCs (531). Interestingly, interference with those pathways by aspirin increased chemosensitivity and decreased self-renewal in breast cancer cells (531). In colorectal cancer cells the inflammatory cytokine IL-6 mediates deacetylation, which subsequently activates NANOG transcription and accumulation of stemness phenotypes, correlating with malignant progression and poor prognosis (543).

To summarize, TIS on the one hand has positive effects that eliminates differentiated tumor cells and also causes invasion of immune cells with anti-tumorigenic functions. On the other hand, senescence causes negative effects that are reflected by pro-tumorigenic functions causing CSC development and a gain of cancer stemness (Figure 2).

An additional level of complexity is added by the plasticity of CSCs as well as non-CSCs, which also causes increased cancer stemness, resistance, and relapse. Examples are given in the next paragraph.

#### Cancer Stemness: Plasticity of CSCs and Non-CSCs

Cancer stemness is not only triggered by senescence escape and acquisition of stemness phenotypes or supported by maintenance of stemness (544) but also by the plasticity of CSCs and non-CSCs, altogether causing tumor relapses after treatment, as described below.

Plasticity is regulated by the TME that is very heterogeneous and consists of CAFs, TAMs, and neutrophils as well as of cancer-associated adipocytes, tumor-infiltrating lymphocytes, and cancer cells with or without stem cell characteristics (545). Therefore, a clear separation between SASP effects and plasticity cannot be made as several direct and also indirect regulatory networks are involved (Figure 2).

Mechanistically, plasticity of cells is a characteristic that ensures robust tissue regeneration and homeostasis (546, 547) and describes the phenotypic and molecular changes of tumor cells increasing stemness and reflecting the tumor's ability to self-renew (18, 548). This phenotype is ultimately closely linked to EMT (15, 548). As described, the transition from the epithelial to mesenchymal state is associated with defined regulatory networks, chromatin remodeling and gene expression programs that are specific to the epithelial, mesenchymal or hybrid cellular state (15–18). Plasticity increases the complexity by suggesting that CSCs can switch between different cellular states, characterized by the expression of surface markers as well as transcription factors (18, 56). Examples for this come from the analysis of different tumor cells: Chaffer et al. demonstrated that CD44^low^ cells (non-CSCs) can switch to a CD44^high^ phenotype (CSCs) resulting in mammosphere formation, a phenotype that could be induced by upregulation of the zinc finger E-box binding homeobox 1 (ZEB1) protein expression induced by TGF-ß (548), which is a major cytokine of the TME (545). In NSCLC cell lines, two distinct CSC subpopulations have been described by expression of CD133 and the aldehyde dehydrogenase (ALDH) (549). ALDHs compose an enzyme superfamily with metabolic functions. The analysis of its activity is often used to identify CSCs (550, 551). Analyzing CD133 and ALDH activity, Akunuru et al. separated cancer stem/progenitor cells (CD133^+^, ALDH^high^) from non-CSCs (CD133^−^ or ALDH^low^) and showed that non-CSCs can interconvert into CSCs. The latter process is activated by TGF-ß signaling or signaling by the zinc finger protein SNAI (Snail) transcription factor family. The described process underlines the dynamic plasticity of CSC/non-CSCs cells (549). After TGF-ß treatment, the authors observed an increase in IL-1ß and IL-6 as well as an increase in CD133^+^ and ALDH^high^ subpopulations (549).

Interferon-ß (IFN-ß) as well as Oncostatin M (OSM), also cytokines within the TME, have been shown to regulate CSC phenotypes (552). Activation of IFN-ß signaling pathways in non-CSCs blocks the expression of CD44 and Snail, which causes a decrease tumor sphere formation and additionally inhibits invasion (552). In contrast, OSM induces a stemness phenotype in non-CSCs (552). One of the major regulators of colorectal tumor plasticity (either CSCs or cancer cells) are the Wnt-ß-catenin and the KRAS/BRAF/ERK pathways, which have been implicated to regulate tumorsphere formation, self-renewal as well as resistance, as reviewed by Pereira et al. (553) and Zhan et al. (554). Activation of Wnt-signaling increased sphere and clone formation as well as drug resistance (555, 556). Acquisition of stemness was also described by Perekatt et al. using transgenic mice to analyze the function of Wnt-signaling in tumorigenesis and de-differentiation in the gut (28). The authors show that the inactivation of Smad 4, a factor that regulates the differentiation program, promoted the development of adenomas with characteristics of activated Wnt signaling over long-term periods (28). Such Wnt activation can correlate with increased treatment resistance as reviewed by Mohammed et al. (557). Also in gastric cancer, activation of the Wnt pathway causes an increase in CD44 as well as Oct-3/4 expression and correlates with an increased proliferation (558).

**Figure 3:**
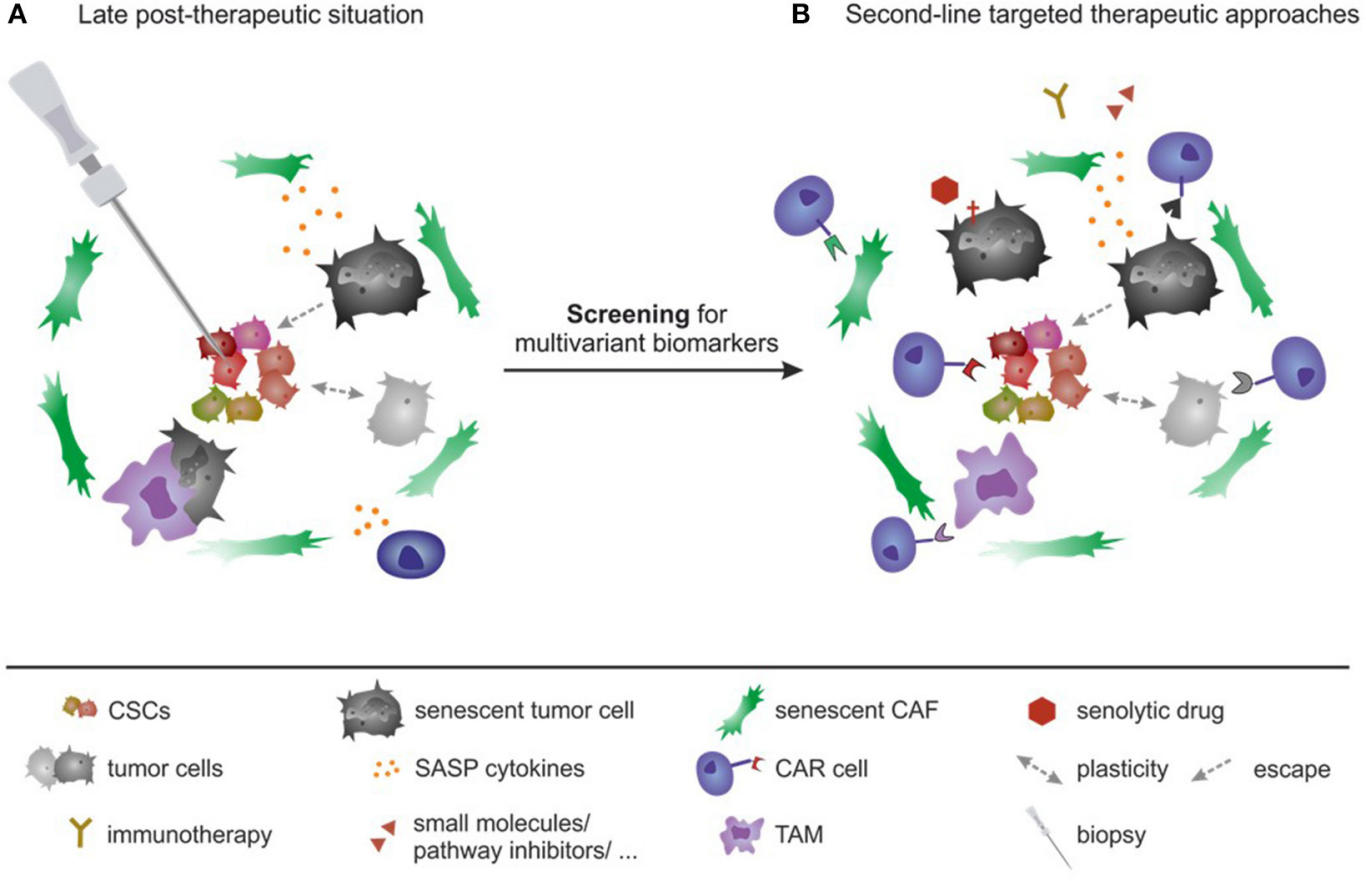
Targeted personalized second-line therapy as a future perspective. **(A)** Analysis of post-therapeutic biopsy samples: follow-up studies need to be included into regular clinical post-therapeutic relapse analysis. After therapy, local biopsies of remaining tumor tissue and/or satellite tissue should be taken periodically (even after several years post-therapy) and a multivariant analysis for biomarkers has to be performed, including the analysis of CSC biomarkers, pro-inflammatory cytokines, senescent markers as well as markers for CAFs. **(B)** Targeted second-line therapy needs to be performed based on the analysis described in **(A)** and will include a specific targeted eradication of remaining cells that could promote tumor relapse and metastasis. Targeted therapies comprise CAR-based approaches targeting CSCs as well as senescent cells or CAFs and TAMs. They also include senolytic drugs to deplete senescent cells independent of CAR approaches.

**Figure 4:**
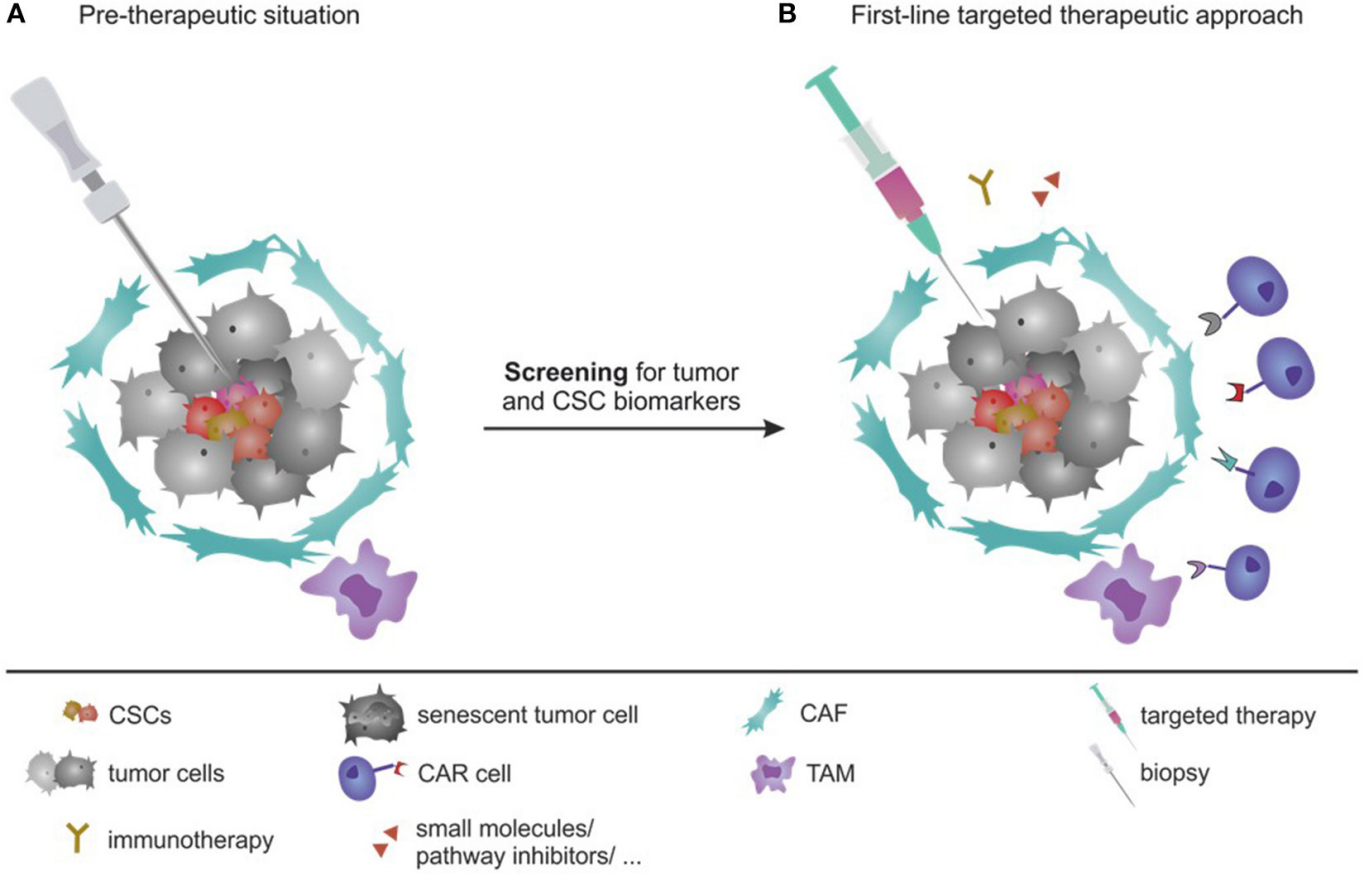
Targeted personalized first-line therapy as a future perspective. **(A)** Pre-therapeutic period: local biopsies before the therapy would allow to determine the heterogenic composition of the tumor, consisting of several biomarkers to be analyzed (CSC, CAFs, and TAMs biomarkers, tumor cell antigens, as well as e.g., T-cell compositions). **(B)** First-line targeted personalized therapeutic approach—therapeutic regimens could combine several approaches: the chemotherapy and small molecules (both selected based on tumor genotype), combined with immunotherapies (antibodies and checkpoint inhibitors based on tumor and analysis of T-cell phenotype), as well as CAR cell-based therapies targeting CSCs, CAFs, and TAMs. Combination therapy will allow a precise and efficient targeting of the heterogenic tumor composition from the beginning on.

As described above, a gain of stemness due to SASP and CSC maintenance or by plasticity of CSCs and non-CSCs, can cause increased resistance (Figure 2). CSCs (pre-existing or post-therapeutically generated *de novo*) can escape the treatment by the expression of drug exporters and detoxification proteins, entrance into dormancy as well as resistance to DNA damage induced cell death (4, 15, 185, 559, 560). Their survival causes tumor relapses (Figure 2). To interfere with the relapse, several strategies have been under investigation to block CSC resistance and growth (9, 13), as described below (Figures 3, 4).

## Eradication of CSCs: New Targeted Approaches

Targeting CSCs has been in the focus of research for many years (13). As reviewed by Shibata and Hoque, the combination of CSC-targeted therapies and conventional non-targeted therapies can result in a decreased chemoresistance (9). Approaches of CSC-targeted therapies include kinase inhibitors as well as targeting stem cell associated pathways such as Wnt and β-catenin, some of which have already entered the clinical phase (9, 13). Immunological approaches that target CSCs via MHC-restricted killing include adoptive cell transfer, targeting checkpoint inhibitors as well as antibody-based approaches and vaccination. MHC-unrestricted killing based on NK-, γδT-, and chimeric antigen receptor (CAR) T-cell approaches have been established (561, 562). Currently, these approaches are performed after failures of the first-line therapies.

Based on the promising results of CAR T-cellular therapy in treating hematological diseases, CAR T-cell-based approaches have also moved forward into the therapy of solid cancers (563, 564). Although, CAR T-cell-based approaches face difficulties in treating solid cancers, their therapeutic use could be a promising alternative (563, 564).

## CAR Therapies Targeting CSCs

### Targeting CD133^+^ CSCs

Targeting CD133^+^ CSCs in solid cancers has shown quite promising preclinical results either using monotherapeutic approaches (565, 566) or using combinational approaches together with cytostatic agents (567). Recently, a clinical trial testing CD133-directed CAR T-cells in patients with ALL, AML, breast, brain, liver, pancreatic and ovarian cancers as well as colorectal cancers has been completed (NCT02541370, Table 9). Initial results showed feasibility, safety, and efficacy of CD133-directed CAR T-cells in patients. Especially, HCC patients who were not responsive to sorafenib showed a median progression-free survival of 7 months (568). In all patients the duration of response ranged from 9 to 63 weeks; three patients showed a continued response at the time of publication. Stable disease was observed in 14 out of 23 patients for 9 weeks to 15.7 months and 21 patients did not show detectable signs of metastasis (568).

**Table 9:** Overview of clinical trials using current CAR-cell-based approaches in solid and hematological cancers targeting CSC.

**Phase**	**ID number**	**Approach**	**Target**	**Cell-based therapy**	**Condition**
I	NCT03423992	CAR T	CD133, EGFRvIII, IL13RvIII2, Her-2,EphA2, GD2,	Autologous CAR T-cells	Recurrent malignant glioma
I	NCT03563326	CAR T	EpCAM	WCH-GC-CAR T	Neoplasm, stomach metastases
I	NCT02915445	CAR T	EpCAM	CAR T-cells	Malignant neoplasm of nasopharynx TNM stagingdistant metastasis (M), Breast cancer recurrent
I	NCT03766126	CAR T	CD123	Autologous CAR T-cells	Relapsed/refractory AML
I	NCT03672851	CAR T	CD123	Autologous CAR T-cells	Relapsed/refractory AML
I	NCT03190278	UCAR T	CD123	Allogeneic CAR T-cells	Relapsed/refractory AML
I	NCT04106076	UCAR T	CD123	Allogeneic CAR T-cells	Newly diagnosed AML
I	NCT02159495	CAR T	CD123	Autologous/allogeneic CAR T-cells	AML (various) or blastic plasmacytoid dendritic cell neoplasms
I	NCT03585517	CAR T	CD123	CAR T-cells	Relapsed/refractory AML
I	NCT04014881	CAR T	CD123	CAR T-cells	Relapsed/refractory AML
I	NCT03114670	CAR T	CD123	Donor-derived CAR T-cells	Recurred AML after allogeneic hematopoetic stem cell transplantation
I	NCT03796390	CAR T	CD123	Autologous CAR T-cells	Relapsed/refractory AML
I	NCT03126864	CAR T	CD33	Autologous CAR T-cells	Relapsed/refractory AML
I	NCT03795779	cCAR T	CLL1-CD33	CAR T-cells	Relapsed and/or refractory, high risk hematologic malignancies
I	NCT02799680	CAR T	CD33	Allogeneic CAR T-cells	Relapsed/refractory AML
I/II	NCT04097301	CAR T	CD44v6	Autologous CAR T- cells	AML, multiple myeloma
I/II	NCT02541370	CAR T	CD133	Autologous or donor-derived T-cells	Liver cancer, pancreatic cancer, brain tumor, breast cancer, ovarian tumor, colorectal cancer, acute myeloid, and lymphoid leukemias
I/II	NCT03356782	CAR T	CD133	Autologous CAR T cells	Sarcoma, osteoid sarcoma, ewing sarcoma
I/II	NCT03013712	CAR T	EpCAM	Autologous CAR T-cells	Colon cancer; esophageal carcinoma; pancreatic, prostate cancer; gastric cancer, hepatic carcinoma
I/II	NCT03556982	CAR T	CD123	Autologous/allogeneic CAR T-cells	Relapsed/refractory AML
I/II	NCT03222674	Multi-CAR T	CD33, CD38, CD123, CD56, MucI, CLL-1	Autologous CAR T-cells	Relapsed/refractory AML
I/II	NCT04010877	Multiple CAR T	CLL-1, CD33, and/or CD123	Autologous/allogeneic CAR T-cells	AML
I/II	NCT04109482	CAR T	CD123	Autologous CAR T-cells	Relapsed or refractory blastic plasmacytoid dendritic cell neoplasm, acute myeloid leukemia, and high risk myelodysplastic syndrome
I/II	NCT02944162	CAR NK	CD33	NK-92-cells	Relapsed/refractory AML
I/II	NCT01864902	CAR T	CD33	Autologous or donor-derived T-cells	Relapsed/refractory AML
I/II	NCT03971799	CAR T	CD33	CAR T-cells	Children and adolescents/young adults (AYAs) with relapsed/refractory acute myeloid leukemia (AML)
II/III	NCT03631576	CAR T	CD123/CLL-1	CAR T-cells	Relapsed/refractory AML
-	NCT03473457	Single or double CAR T	CD33,CD38, CD56, CD123, CD117, CD133,CD34, or Mucl	CAR T-cells	Relapsed/refractory AML
II	NCT02729493	CAR T	EpCAM	Autologous CAR T-cells	Relapsed or refractory liver cancer
II	NCT02725125	CAR T	EpCAM	Autologous CAR T-cells	Relapsed or refractory stomach cancer
N.A.	NCT04151186	CAR T	EpCAM,TM4SF1	CAR T-cells	Solid tumor

*Source: http://clinicaltrials.gov/*.

Additional studies (Table 9) are ongoing for the treatment of relapsed or refractory AML (NCT03473457), relapsed or late staged sarcoma (NCT03356782), as well as glioma (NCT03423992). A case study of a patient receiving CD133-directed CAR T-cells after previous chemo- and radiotherapy as well as EGFR-directed CAR T-cell therapy reported a partial response for a period of 4.5 months (569). However, severe toxicities affecting the skin, the oral mucosa, and the gastrointestinal tract were reported (569).

### Targeting CD44^+^ CSCs

Although CD44 is a very prominent CSC antigen, only few CAR-based approaches targeting CD44 have been developed. Early approaches that entered clinical trials included monoclonal antibodies and antibody-conjugates. First studies involving ^186^Re-conjugated antibody against the splice variant CD44v6 showed advantageous effects at first, however a long-term stable disease was only observed in one patient (570, 571). Likewise, the CD44-directed monoclonal antibody RG7356 showed only modest success in clinical trials with AML patients (572) and solid tumors (468). Tijink et al. coupled the CD44v6-directed antibody bivatuzumab to the cytotoxic antimicrotubule agent mertansine to produce an antibody-prodrug conjugate (573). Bivatuzumab mertansine was administered to seven patients and two of them showed stable disease during the therapy phase. However, one patient with squamous cell carcinoma of the esophagus died after treatment due to toxic epidermal necrolysis, which caused the premature cancelation of this trial (573). Because of this fatality, two clinical trials that were conducted in parallel for patients with metastatic breast cancer (574) and head and neck squamous cell carcinoma (575) had to be terminated.

Still, there are some promising approaches involving CD44v6-directed CAR therapies. For instance, cytokine-induced killer (CIK) cells carrying a CAR against CD44v6 showed anti-cancer effects against sarcoma *in vitro* and *in vivo* (576). Furthermore, a phase I/IIa clinical trial using CD44v6-directed CAR T-cells for AML and multiple myeloma patients is currently recruiting (NCT04097301) (Table 9).

### Targeting EpCAM^+^ CSCs

Pre-clinical as well as clinical studies targeting EpCAM^+^ cancer cells using monoclonal antibodies or CAR constructs have been performed to date using co-culture and xenograft approaches (577–579) (Table 9). Combination therapy of EpCAM-directed CAR NK-92-cells and regorafenib, a potent multikinase inhibitor, resulted in a synergistic antitumor effect using for example colorectal cancer cells or xenograft models (580). CAR T-cells targeting EpCAM have been shown to significantly block tumor growth in xenografts and to secrete cytotoxic cytokines, including interferon-γ (IFN-γ) and tumor necrosis factor alpha (TNF-α) *in vitro* (579). Additionally, an injection of EpCAM-directed CAR T-cells led to delayed disease progression in immunodeficient mice with peritoneal ovarian and colorectal xenografts (581). Currently, there are several clinical trials with EpCAM-directed CAR T-cells listed for patients with various malignancies: three trials are ongoing (NCT02915445, NCT03563326, and NCT03013712), one trial is not yet recruiting (NCT04151186), and four trials are listed with unknown status (NCT02725125, NCT02728882, NCT02735291, and NCT02729493) (Table 9).

### LSC-Directed CAR Therapies

In the field of CAR therapeutics, CD123 and CD33 are frequent targets for AML-specific CAR cells (Table 9). CAR T- and CAR NK-92-cells redirected against CD33 have entered clinical trials (Table 9). Case reports show a good tolerability of CD33-directed CAR NK-92-cells (372), but disease progression after treatment with CD33-directed CAR T-cells was still present (387). Currently, numerous clinical trials using CAR T-cells targeting CD123 are ongoing. NCT03672851 with two participants had to be terminated due to adverse effects (582). Furthermore, first studies implement CLL-1 as a target of CAR T-cells [Table 9; (419), NCT04010877 and NCT03222674].

## Next Generation CARs and Targeting of CSCs in Combinational Therapies

For the more efficient CSC elimination, different approaches that have been developed can be used, i.e., tandem CAR T-cells (TanCAR) (583) as well as single universal (U) tricistronic transgene CAR T-cells (UCAR T-cells) (584). Multi-targeting of Her2, IL-13 receptor subunit alpha-2 (IL13Rα2), and ephrin-A2 (EphA2) was shown to overcome antigenic heterogeneity in 15 primary GBM samples and to increase the therapeutic success using xenograft models (584). Targeting two or more antigens may increase the risk for on-target/off-tumor toxicity, since most of the antigens are not only expressed on malignant cells, but also on healthy cells (60, 585). Improved safety, specificity, and flexibility can be obtained using universal CARs (UniCAR) or split, universal and programmable (SUPRA) CARs (585–589). Both consist of an inert and universal CAR construct without a single chain variable fragment (scFv) adaptor molecule domain in combination with a multiple tumor-targeting scFv adaptor molecule (585, 588, 589). In both cases, the activity of CAR T-cells can be regulated by the dosage of the scFv adaptor molecules or by introducing competitive molecules, such as leucine zippers as a regulator for the SUPRA CARs (588, 589). Additional safety of CAR T-cells can be achieved by the induction of suicide genes, e.g., iCasp9 (590, 591) or by inhibitory CAR (iCAR) constructs, in which signaling domains consist of an immuno-inhibitory receptor [e.g., CTLA-4 or PD-1; (592)]. An antigen only expressed on the surface of healthy cells is a target of iCAR and therefore the the attack of non-tumorigenic cells is greatly reduced (592). Specificity can be improved by using synthetic Notch (synNotch) receptors. The binding of synNotch specific to the antigen induces the cleavage of an intracellular domain and activates in turn the transcription of a second CAR, specific to another tumor antigen (593).

To enhance the targeting of solid tumors using CAR-based approaches, the combination treatment with conventional chemotherapeutic drugs could be a novel strategy to enhance antitumor response. To test this approach, NK-92 cells were modified with an EGFR-directed CAR construct against renal cell carcinoma (RCC) cell lines (594). In combination with the multikinase inhibitor cabozantinib, EGFR-directed CAR NK-92 cells showed synergistic effects *in vitro* and *in vivo* (594). Cabozantinib also caused a decrease of the anti-inflammatory PD-L1 surface expression in renal cell carcinoma cell lines (594). Furthermore, cabozantinib is known to reduce tumor infiltration of immuno-modulatory subpopulations like regulatory T-cells (Tregs) and myeloid-derived suppressor cells (MDSCs) (594, 595).

The combination of the multikinase inhibitor sunitinib and CAR T -cells targeting carbonic anhydrase IX (CAIX) has been shown to be of advantage as sunitinib reduces immunosuppressive components of the TME (596). Improvements could also be made using Her2-directed CAR NK-92-cells (92/5.137.z) in combination with apatinib (597). Treatment with CAR NK-92 alone resulted in an efficient elimination of small Her2^+^ tumor xenografts *in vivo*, but not in an elimination of larger solid tumors in gastric cancers (597). A combinatorial treatment with apatinib increased CAR NK-92 cell infiltration into these larger tumor xenografts and resulted in an enhanced antitumor efficacy of the cells (597).

In AML, early approaches focused on the targeting of single markers; combinatorial therapies, targeting more than one marker, have been tested here as well (598). Haubner et al. analyzed optimal combinations of different LSC markers and concluded that CD33/TIM-3 or CLL-1/TIM-3 combinatorial targeting is most suitable since these markers maximally cover AML cells and are minimally co-expressed on HSCs (370). Interestingly, the combination of CD33 and CD123 was found unsuitable (370). Approaches that already implement combinatorial targeting of AML LSCs include tri-specific killer engagers against CD33 and CD123 (373), compound CAR T-cells against CD33 and CD123 (374) or CLL-1 and CD33 (i.e., NCT03795779), universal CAR T-cells against CD33 and CD123 (375), and CAR CIK-cells against CD33 and CD123 (376).

## Future Perspectives

Studies obtained in the last 5–10 years confirmed the importance and the urgent need of diagnostic screening of the TME not only before the treatment, but also at several stages in the post-therapeutic period. This is within the context of personalized therapies that are based on the idea to identify the best therapeutic approach for the patient. This approach should be based on the tumors molecular signature, involving the TME. The best and the most appropriate therapeutic options, which match each individual patient's requirements will increase the therapeutic efficacy and will cause fewer side effects.

The particular value of post-therapeutic local biopsies is that they enable the evaluation of tumor relapse risk on the basis of multivariate biomarkers and also provide information on therapeutically addressable targets within the remaining tumor tissue. In-time detection of tumor-promoting cells, which re-emerge in the post-therapeutic period (Figure 3), will allow an application of the individualized and precise second-line therapy in a timely fashion. Detection of tumor cells with stemness phenotypes will allow for their directed and specific targeting using the second-line treatments, depending on a different mode of action (4, 560). This secondary specific therapy can include, targeted therapies such as e.g., immunotherapies, CAR NK-, and CAR T-cells that mediate a precise eradication of several types of cells: CSCs, CAFs, and/or remaining senescent cells. To increase the specificity and therapeutic outcome and to decrease severe side effects, CAR-based therapeutics are constantly being optimized, as discussed in the section above. Special needs are: improvement of target specificity in combination with decreased off-target effects. In addition, secondary therapies could also include senolytic drugs that selectively kill senescent cells as it was discussed in a recent comprehensive review by Short et al. (599). These therapies cause very low or minor side-effects after their administration (599). In the post-therapeutic period, however, it is important to focus on the biomarkers of CSCs as well as the biomarkers of senescent tumor cells, tumor-promoting SASP molecules, CAFs and TAMs. These cells and molecules strongly influence tumor relapse and their monitoring and their in-time elimination is crucial (Figure 3). As currently available blood test systems are not sensitive enough to detect local changes in the TME, other methods for instance local biopsies and subsequent multivariant analysis of obtained tissues should be used whenever possible and even after many years upon the first-line therapy (Figure 3).

The analysis of multivariant biomarker, however is not only of importance within the post-therapeutic situation. A detailed understanding of the tumor composition before the treatment could allow straight forward first line therapies (Figure 4). Target analysis includes CSCs, CAFs, tumors cells and TAMs, and other tumor-promoting cells. Therapeutic options such as chemotherapy and radiotherapy in combination with small molecules and immunotherapies (CAR cells) could tremendously improve the outcome of the first-line approaches and predict relapses (Figure 4). Combinations already in the first-line therapy are especially required in advanced stages of malignant disease.

In conclusion, our review gives an overview of the most important biomarkers of CSCs in the TME. Furthermore, we underline the value of local biopsies and precise diagnostics and screening of biomarkers in both pre- and post-therapeutic situations (Figures 3, 4). We suggest the implementation of those strategies in the first and second-line personalized therapy required to eradicate the remaining tumor-promoting senescent tumor cells, CAFs, TAMs, and finally CSCs to protect from tumor recurrence.

The high costs are one point of contention regarding the biopsies and their analysis as well as the implementation of immunotherapies into the first and secondary line targeted therapies. However, considering the costs for therapies, comprising resection, and medication strategies, as well as the patient's sufferings due to a re-emerged full-blown cancer, the targeted therapy will help to save the patients and clinics from high personnel, emotional, and medicinal costs.

## Author Contributions

SF, UK-B, and TY performed a conceptualization for the review and defined the future perspectives. LW, A-KK, HS, RK, SD, AS, A-RB, TY, SF, and UK-B analyzed the publications and created the figures and tables. All authors contributed to the article and approved the submitted version.

## Conflict of Interest

The authors declare that the research was conducted in the absence of any commercial or financial relationships that could be construed as a potential conflict of interest.

## References

[1] Bray F Ferlay J Soerjomataram I Siegel RL Torre LA Jemal A. Global cancer statistics 2018: globocan estimates of incidence and mortality worldwide for 36 cancers in 185 countries. CA Cancer J Clin. (2018) 68:394–424. 10.3322/caac.21492 30207593

[2] Capp J-P. Cancer stem cells: from historical roots to a new perspective. J Oncol. (2019) 2019:5189232. 10.1155/2019/5189232 31308849 PMC6594320

[3] Spillane JB Henderson MA. Cancer stem cells: a review. ANZ J Surg. (2007) 77:464–8. 10.1111/j.1445-2197.2007.04096.x 17501888

[4] Phi LT Sari IN Yang Y-G Lee S-H Jun N Kim KS. Cancer Stem Cells (CSCs) in drug resistance and their therapeutic implications in cancer treatment. Stem Cells Int. (2018) 2018:5416923. 10.1155/2018/5416923 29681949 PMC5850899

[5] Reya T Morrison SJ CLarke MF Weissman IL. Stem cells, cancer, and cancer stem cells. Nature. (2001) 414:105–11. 10.1038/35102167 11689955

[6] Ayob AZ Ramasamy TS. Cancer stem cells as key drivers of tumour progression. J Biomed Sci. (2018) 25:20. 10.1186/s12929-018-0426-4 29506506 PMC5838954

[7] Kuşoglu A Biray Avci Ç. Cancer stem cells: a brief review of the current status. Gene. (2019) 681:80–5. 10.1016/j.gene.2018.09.052 30268439

[8] Arnold CR Mangesius J Skvortsova I-I Ganswindt U. The role of cancer stem cells in radiation resistance. Front. Oncol. (2020) 10:164. 10.3389/fonc.2020.00164 32154167 PMC7044409

[9] Shibata M Hoque MO. Targeting cancer stem cells: a strategy for effective eradication of cancer. Cancers. (2019) 11:732. 10.3390/cancers11050732 31137841 PMC6562442

[10] Zeng S Shen WH Liu L Senescence and cancer. Cancer Transl Med. (2018) 4:70–4. 10.4103/ctm.ctm_22_18 30766922 PMC6372122

[11] Scuric Z Carroll JE Bower JE Ramos-Perlberg S Petersen L Esquivel S. Biomarkers of aging associated with past treatments in breast cancer survivors. NPJ Breast Cancer. 3:1–7. 10.1038/s41523-017-0050-6 29238750 PMC5727230

[12] Fan DN Schmitt CA Detecting markers of therapy-induced senescence in cancer cells. In: Nikiforov MA editor. Oncogene-Induced Senescence: Methods and Protocols. New York, NY: Humana Press (2017). p. 41–52. 10.1007/978-1-4939-6670-7_4 27812866

[13] Desai A Yan Y Gerson SL. Concise reviews: cancer stem cell targeted therapies: toward clinical success. Stem Cells Transl Med. (2019) 8:75–81. 10.1002/sctm.18-0123 30328686 PMC6312440

[14] Milanovic M Fan DN Belenki D Däbritz JH Zhao Z Yu Y. Senescence-associated reprogramming promotes cancer stemness. Nature. (2018) 553:96–100. 10.1038/nature25167 29258294

[15] Gupta PB Pastushenko I Skibinski A Blanpain C Kuperwasser C. Phenotypic plasticity: driver of cancer initiation, progression, and therapy resistance. Cell Stem Cell. (2019) 24:65–78. 10.1016/j.stem.2018.11.011 30554963 PMC7297507

[16] Poli V Fagnocchi L Zippo A. Tumorigenic cell reprogramming and cancer plasticity: interplay between signaling, microenvironment, and epigenetics. Stem Cells Int. (2018) 2018:4598195. 10.1155/2018/4598195 29853913 PMC5954911

[17] Angelis ML de Francescangeli F La Torre F Zeuner A. Stem cell plasticity and dormancy in the development of cancer therapy resistance. Front. Oncol. (2019) 9:626. 10.3389/fonc.2019.00626 31355143 PMC6636659

[18] Yuan S Norgard RJ Stanger BZ Cellular plasticity in cancer. Cancer Discov. (2019) 9:837–51. 10.1158/2159-8290.CD-19-0015 30992279 PMC6606363

[19] Hanahan D Weinberg RA. Hallmarks of cancer: the next generation. Cell. (2011) 144:646–74. 10.1016/j.cell.2011.02.013 21376230

[20] Basu AK DNA damage, mutagenesis and cancer. Int J Mol Sci. (2018) 19:970 10.3390/ijms19040970 PMC5979367 29570697

[21] Blackadar CB. Historical review of the causes of cancer. World J Clin Oncol. (2016) 7:54–86. 10.5306/wjco.v7.i1.54 26862491 PMC4734938

[22] Li L Neaves WB. Normal stem cells and cancer stem cells: the niche matters. Cancer Res. (2006) 66:4553–7. 10.1158/0008-5472.CAN-05-3986 16651403

[23] Sell S Cellular origin of cancer: dedifferentiation or stem cell maturation arrest? Environ Health Perspect. (1993) 101(Suppl. 5):15–26. 10.1289/ehp.93101s515 PMC1519468 7516873

[24] Hayakawa Y Fox JG Wang TC. The origins of gastric cancer from gastric stem cells: lessons from mouse models. Cell Mol Gastroenterol Hepatol. (2017) 3:331–8. 10.1016/j.jcmgh.2017.01.013 28462375 PMC5404024

[25] Koulis A Buckle A Boussioutas A. Premalignant lesions and gastric cancer: current understanding. World J Gastroentero Oncol. (2019) 11:665–78. 10.4251/wjgo.v11.i9.665 31558972 PMC6755108

[26] Hata M Hayakawa Y Koike K. Gastric stem cell and cellular origin of cancer. Biomedicines. (2018) 6:100. 10.3390/biomedicines6040100 30384487 PMC6315982

[27] Li X-B Yang G Zhu L Tang Y-L Zhang C Ju Z. Gastric Lgr5+ stem cells are the cellular origin of invasive intestinal-type gastric cancer in mice. Cell Res. (2016) 26:838–49. 10.1038/cr.2016.47 27091432 PMC5129876

[28] Perekatt AO Shah PP Cheung S Jariwala N Wu A Gandhi V. SMAD4 suppresses WNT-driven dedifferentiation and oncogenesis in the differentiated gut epithelium. Cancer Res. (2018) 78:4878–90. 10.1158/0008-5472.CAN-18-0043 29986996 PMC6125228

[29] Mu X Español-Suñer R Mederacke I Affò S Manco R Sempoux C. Hepatocellular carcinoma originates from hepatocytes and not from the progenitor/biliary compartment. J Clin Invest. (2015) 125:3891–903. 10.1172/JCI77995 26348897 PMC4607132

[30] Jörs S Jeliazkova P Ringelhan M Thalhammer J Dürl S Ferrer J. Lineage fate of ductular reactions in liver injury and carcinogenesis. J Clin Invest. (2015) 125:2445–57. 10.1172/JCI78585 25915586 PMC4497753

[31] Holczbauer Á Factor VM Andersen JB Marquardt JU Kleiner DE Raggi C. Modeling pathogenesis of primary liver cancer in lineage-specific mouse cell types. Gastroenterology. (2013) 145:221–31. 10.1053/j.gastro.2013.03.013 23523670 PMC3913051

[32] Oikawa T. Cancer stem cells and their cellular origins in primary liver and biliary tract cancers. Hepatology. (2016) 64:645–51. 10.1002/hep.28485 26849406

[33] Polyak K. Breast cancer: origins and evolution. J Clin Invest. (2007) 117:3155–63. 10.1172/JCI33295 17975657 PMC2045618

[34] Zarzynska JM Chapter 12: The role of stem cells in breast cancer. In: Van Pham P editor. Breast Cancer - From Biology to Medicine. VNU-HCM University of Science; INTECH Open (2017) 231–49. 10.5772/66904

[35] Zhou J Chen Q Zou Y Chen H Qi L Chen Y. Stem cells and cellular origins of breast cancer: updates in the rationale, controversies, and therapeutic implications. Front. Oncol. (2019) 9:820. 10.3389/fonc.2019.00820 31555586 PMC6722475

[36] Wang J Xu B. Targeted therapeutic options and future perspectives for HER2-positive breast cancer. Sig Transduct Target Ther. (2019) 4:1–22. 10.1038/s41392-019-0069-2 31637013 PMC6799843

[37] Sánchez-Danés A Blanpain C. Deciphering the cells of origin of squamous cell carcinomas. Nat Rev Cancer. (2018) 18:549–61. 10.1038/s41568-018-0024-5 29849070 PMC7170720

[38] Yamano S Gi M Tago Y Doi K Okada S Hirayama Y. Role of deltaNp63(pos)CD44v(pos) cells in the development of n-nitroso-tris-chloroethylurea-induced peripheral-type mouse lung squamous cell carcinomas. Cancer Sci. (2016) 107:123–32. 10.1111/cas.12855 26663681 PMC4768398

[39] Hardavella G George R Sethi T. Lung cancer stem cells—characteristics, phenotype. Transl Lung Cancer Res. (2016) 5:272–9. 10.21037/tlcr.2016.02.01 27413709 PMC4931140

[40] Fukui T Shaykhiev R Agosto-Perez F Mezey JG Downey RJ Travis WD. Lung adenocarcinoma subtypes based on expression of human airway basal cell genes. Eur Respir J. (2013) 42:1332–44. 10.1183/09031936.00144012 23645403 PMC4124529

[41] Kim CF Jackson EL Woolfenden AE Lawrence S Babar I Vogel S. Identification of bronchioalveolar stem cells in normal lung and lung cancer. Cell. (2005) 121:823–35. 10.1016/j.cell.2005.03.032 15960971

[42] Sutherland KD Proost N Brouns I Adriaensen D Song J-Y Berns A. Cell of origin of small cell lung cancer: inactivation of trp53 and rb1 in distinct cell types of adult mouse lung. Cancer Cell. (2011) 19:754–64. 10.1016/j.ccr.2011.04.019 21665149

[43] Bonnet D Dick JE. Human acute myeloid leukemia is organized as a hierarchy that originates from a primitive hematopoietic cell. Nat Med. (1997) 3:730–7. 10.1038/nm0797-730 9212098

[44] Czeh M Rosenbauer F. Uncovering a new cellular origin for acute myeloid leukemia with lineage plasticity. Mol Cell Oncol. (2017) 4:e1268241. 10.1080/23723556.2016.1268241 28401180 PMC5383360

[45] Riemke P Czeh M Fischer J Walter C Ghani S Zepper M. Myeloid leukemia with transdifferentiation plasticity developing from T-cell progenitors. EMBO J. (2016) 35:2399–416. 10.15252/embj.201693927 27572462 PMC5109237

[46] Stuart SA Minami Y Wang JY. The CML stem cell: evolution of the progenitor. Cell CyCLe. (2009) 8:1338–43. 10.4161/cc.8.9.8209 19342894 PMC2792634

[47] Zhou H Xu R. Leukemia stem cells: the root of chronic myeloid leukemia. Protein Cell. (2015) 6:403–12. 10.1007/s13238-015-0143-7 25749979 PMC4444810

[48] Ebinger S Özdemir EZ Ziegenhain C Tiedt S Castro Alves C Grunert M. Characterization of rare, dormant, and therapy-Resistant cells in acute lymphoblastic leukemia. Cancer Cell. (2016) 30:849–62. 10.1016/j.ccell.2016.11.002 27916615 PMC5156313

[49] Martinez-CLiment JA Fontan L Gascoyne RD Siebert R Prosper F. Lymphoma stem cells: enough evidence to support their existence? Haematologica. (2010) 95:293–302. 10.3324/haematol.2009.013318 20139392 PMC2817033

[50] Lang F Wojcik B Rieger MA. Stem cell hierarchy and clonal evolution in acute lymphoblastic leukemia. Stem Cells Int. (2015) 2015:137164. 10.1155/2015/137164 26236346 PMC4506911

[51] Afify SM Seno M. Conversion of stem cells to cancer stem cells: undercurrent of cancer initiation. Cancers. (2019) 11:345. 10.3390/cancers11030345 30862050 PMC6468812

[52] Bu P Chen K-Y Lipkin SM Shen X. Asymmetric division: a marker for cancer stem cells? Oncotarget. (2013) 4:950–1. 10.18632/oncotarget.1029 23807730 PMC3759670

[53] Goardon N Marchi E Atzberger A Quek L Schuh A Soneji S. Coexistence of lMPP-like and gMP-like leukemia stem cells in acute myeloid leukemia. Cancer Cell. (2011) 19:138–52. 10.1016/j.ccr.2010.12.012 21251617

[54] Quek L Otto GW Garnett C Lhermitte L Karamitros D Stoilova B. Genetically distinct leukemic stem cells in human CD34- acute myeloid leukemia are arrested at a hemopoietic precursor-like stage. J Exp Med. (2016) 213:1513–35. 10.1084/jem.20151775 27377587 PMC4986529

[55] Chopra M Bohlander SK. The cell of origin and the leukemia stem cell in acute myeloid leukemia. Genes Chromosomes Cancer. (2019) 58:850–8. 10.1002/gcc.22805 31471945

[56] Cabrera MC Hollingsworth RE Hurt EM. Cancer stem cell plasticity and tumor hierarchy. World J Stem Cells. (2015) 7:27–36. 10.4252/wjsc.v7.i1.27 25621103 PMC4300934

[57] Rich JN. Cancer stem cells: understanding tumor hierarchy and heterogeneity. Medicine. (2016) 95:S2–7. 10.1097/MD.0000000000004764 27611934 PMC5599210

[58] Berger DP Mertelsmann R editors. Das Rote Buch: Hämatologie und Internistische Onkologie. Landsberg am Lech: ecomed MEDIZIN (2017). p. 1400.

[59] Sell S. Alpha-fetoprotein, stem cells and cancer: how study of the production of alpha-fetoprotein during chemical hepatocarcinogenesis led to reaffirmation of the stem cell theory of cancer. Tumour Biol. (2008) 29:161–80. 10.1159/000143402 18612221 PMC2679671

[60] Kim W-T Ryu CJ. Cancer stem cell surface markers on normal stem cells. BMB Rep. (2017) 50:285–98. 10.5483/BMBRep.2017.50.6.039 28270302 PMC5498139

[61] Su J Wu S Wu H Le Li Guo T. CD44 is functionally crucial for driving lung cancer stem cells metastasis through Wnt/β-catenin-FoxM1-Twist signaling. Mol Carcinog. (2016) 55:1962–73. 10.1002/mc.22443 26621583

[62] Satar NA Fakiruddin KS Lim MN Mok PL Zakaria N Fakharuzi NA. Novel triplepositive markers identified in human nonsmall cell lung cancer cell line with chemotherapy-resistant and putative cancer stem cell characteristics. Oncol Rep. (2018) 40:669–81. 10.3892/or.2018.6461 29845263 PMC6072294

[63] Hu B Ma Y Yang Y Zhang L Han H Chen J. CD44 promotes cell proliferation in non-small cell lung cancer. Oncol Lett. (2018) 15:5627–33. 10.3892/ol.2018.8051 29552200 PMC5840516

[64] Suda K Murakami I Yu H Kim J Tan A-C Mizuuchi H. CD44 facilitates epithelial-to-Mesenchymal transition phenotypic change at acquisition of resistance to EGFR kinase inhibitors in lung cancer. Mol Cancer Ther. (2018) 17:2257–65. 10.1158/1535-7163.MCT-17-1279 30049789

[65] Nishino M Ozaki M Hegab AE Hamamoto J Kagawa S Arai D. Variant CD44 expression is enriching for a cell population with cancer stem cell-like characteristics in human lung adenocarcinoma. J Cancer. (2017) 8:1774–85. 10.7150/jca.19732 28819374 PMC5556640

[66] Zakaria N Yusoff NM Zakaria Z Lim MN Baharuddin PJ Fakiruddin KS. Human non-small cell lung cancer expresses putative cancer stem cell markers and exhibits the transcriptomic profile of multipotent cells. BMC Cancer. (2015) 15:84. 10.1186/s12885-015-1086-3 25881239 PMC4349658

[67] Prabavathy D SwaRNAlatha Y Ramadoss N. Lung cancer stem cells-origin, characteristics and therapy. Stem Cell Investig. (2018) 5:6. 10.21037/sci.2018.02.01 29682513 PMC5897668

[68] Testa U Castelli G Pelosi E. Lung cancers: molecular characterization, clonal heterogeneity and evolution, and cancer stem cells. Cancers. (2018) 10:248. 10.3390/cancers10080248 30060526 PMC6116004

[69] Maiuthed A Chantarawong W Chanvorachote P. Lung cancer stem cells and cancer stem cell-targeting natural compounds. Anticancer Res. (2018) 38:3797–809. 10.21873/anticanres.12663 29970499

[70] Park E Park SY Sun P-L Jin Y Kim JE Jheon S. Prognostic significance of stem cell-related marker expression and its correlation with histologic subtypes in lung adenocarcinoma. Oncotarget. (2016) 7:42502–12. 10.18632/oncotarget.9894 27285762 PMC5173151

[71] Alamgeer M Neil Watkins D Banakh I Kumar B Gough DJ Markman B. A phase IIa study of HA-irinotecan, formulation of hyaluronic acid and irinotecan targeting CD44 in extensive-stage small cell lung cancer. Invest New Drugs. (2018) 36:288–98. 10.1007/s10637-017-0555-8 29277856

[72] Quan YH Lim J-Y Choi BH Choi Y Choi YH Park J-H. Self-targeted knockdown of CD44 improves cisplatin sensitivity of chemoresistant non-small cell lung cancer cells. Cancer Chemother Pharmacol. (2019) 83:399–410. 10.1007/s00280-018-3737-y 30515553

[73] Kawano Y Iwama E Tsuchihashi K Shibahara D Harada T Tanaka K. CD44 variant-dependent regulation of redox balance in EGFR mutation-positive non-small cell lung cancer: a target for treatment. Lung Cancer. (2017) 113:72–8. 10.1016/j.lungcan.2017.09.008 29110853

[74] Huang X Wan J Leng D Zhang Y Yang S. Dual-targeting nanomicelles with CD133 and CD44 aptamers for enhanced delivery of gefitinib to two populations of lung cancer-initiating cells. Exp Ther Med. (2020) 19:192–204. 10.3892/etm.2019.8220 31853290 PMC6909660

[75] Luo Y Wang X Du D Lin Y. Hyaluronic acid-conjugated apoferritin nanocages for lung cancer targeted drug delivery. Biomater Sci. (2015) 3:1386–94. 10.1039/C5BM00067J 26301700

[76] Alshaer W Hillaireau H Vergnaud J Ismail S Fattal E. Functionalizing liposomes with anti-CD44 aptamer for selective targeting of cancer cells. Bioconjug Chem. (2015) 26:1307–13. 10.1021/bc5004313 25343502

[77] Song Y Cai H Yin T Huo M Ma P Zhou J. PaCLitaxel-loaded redox-sensitive nanopartiCLes based on hyaluronic acid-vitamin E succinate conjugates for improved lung cancer treatment. Int J Nanomed. (2018) 13:1585–600. 10.2147/IJN.S155383 29588586 PMC5858821

[78] Parashar P Rathor M Dwivedi M Saraf SA. Hyaluronic acid decorated naringenin nanoparticles: appraisal of chemopreventive and curative potential for lung cancer. Pharmaceutics. (2018) 10:33. 10.3390/pharmaceutics10010033 29534519 PMC5874846

[79] Tian Y Zhang H Qin Y Li D Liu Y Wang H. Overcoming drug-resistant lung cancer by paCLitaxel-loaded hyaluronic acid-coated liposomes targeted to mitochondria. Drug Dev Ind Pharm. (2018) 44:2071–82. 10.1080/03639045.2018.1512613 30112929

[80] Zhang W Xu W Lan Y He X Liu K Liang Y. Antitumor effect of hyaluronic-acid-modified chitosan nanopartiCLes loaded with siRNA for targeted therapy for non-small cell lung cancer. Int J Nanomedicine. (2019) 14:5287–301. 10.2147/IJN.S203113 31406460 PMC6642624

[81] Shinohara S Hanagiri T Taira A Takenaka M Oka S Chikaishi Y. Immunohistochemical expression and serum levels of CD44 as prognostic indicators in patients with non-small cell lung cancer. Oncology. (2016) 90:327–38. 10.1159/000445951 27225749

[82] Qiu X Wang Z Li Y Miao Y Ren Y Luan Y. Characterization of sphere-forming cells with stem-like properties from the small cell lung cancer cell line H446. Cancer Lett. (2012) 323:161–70. 10.1016/j.canlet.2012.04.004 22521544

[83] Yan X Luo H Zhou X Zhu B Wang Y Bian X. Identification of CD90 as a marker for lung cancer stem cells in a549 and h446 cell lines. Oncol Rep. (2013) 30:2733–40. 10.3892/or.2013.2784 24101104

[84] Koren A Rijavec M Kern I Sodja E Korosec P Cufer T. BMI1, ALDH1A1, and CD133 transcripts connect epithelial-mesenchymal transition to cancer stem cells in lung carcinoma. Stem Cells Int. (2016) 2016:9714315. 10.1155/2016/9714315 26770215 PMC4685144

[85] Alama A Gangemi R Ferrini S Barisione G Orengo AM Truini M. CD133-Positive cells from non-Small cell lung cancer show distinct sensitivity to cisplatin and afatinib. Arch Immunol Ther Exp. (2015) 63:207–14. 10.1007/s00005-015-0330-5 25678473

[86] Eramo A Lotti F Sette G Pilozzi E Biffoni M Di Virgilio A. Identification and expansion of the tumorigenic lung cancer stem cell population. Cell Death Differ. (2008) 15:504–14. 10.1038/sj.cdd.4402283 18049477

[87] Huang Z Yu H Zhang J Jing H Zhu W Li X. Correlation of cancer stem cell markers and immune cell markers in resected non-small cell lung cancer. J Cancer. (2017) 8:3190–7. 10.7150/jca.20172 29158791 PMC5665035

[88] Li L Li J-C Yang H Zhang X Liu L-L Li Y. Expansion of cancer stem cell pool initiates lung cancer recurrence before angiogenesis. Proc Natl Acad Sci USA. (2018) 115:E8948–57. 10.1073/pnas.1806219115 30158168 PMC6156672

[89] Liu X Wei H Liu Y Zhao M Wu S Yang Y. Construction of high sensitive CD133 immune PLGA magnetic spheres platform for lung cancer stem cells isolation and its property evaluation. J Biomed Nanotechnol. (2018) 14:1066–74. 10.1166/jbn.2018.2562 29843871

[90] Su Y-J Lin W-H Chang Y-W Wei K-C Liang C-L Chen S-C. Polarized cell migration induces cancer type-specific CD133/integrin/Src/Akt/GSK3β/β-catenin signaling required for maintenance of cancer stem cell properties. Oncotarget. (2015) 6:38029–45. 10.18632/oncotarget.5703 26515729 PMC4741982

[91] Bertolini G D'Amico L Moro M Landoni E Perego P Miceli R. Microenvironment-modulated metastatic CD133+/CXCR4+/EpCAM- lung cancer-Initiating cells sustain tumor dissemination and correlate with poor prognosis. Cancer Res. (2015) 75:3636–49. 10.1158/0008-5472.CAN-14-3781 26141860

[92] Chen Y Zhang F Tsai Y Yang X Yang L Duan S. IL-6 signaling promotes DNA repair and prevents apoptosis in CD133+ stem-like cells of lung cancer after radiation. Radiat Oncol. (2015) 10:227. 10.1186/s13014-015-0534-1 26572130 PMC4647293

[93] Zhao W Luo Y Li B Zhang T. Tumorigenic lung tumorospheres exhibit stem-like features with significantly increased expression of CD133 and ABCG2. Mol Med Rep. (2016) 14:2598–606. 10.3892/mmr.2016.5524 27432082 PMC4991750

[94] Koyama K Katsurada N Jimbo N Tachihara M Tamura D Nakata K. Overexpression of CD 133 and bCL-2 in non-small cell lung cancer with neuroendocrine differentiation after transformation in ALK rearrangement-positive adenocarcinoma. Pathol Int. (2019) 69:294–9. 10.1111/pin.12782 30900377

[95] Moro M Bertolini G Caserini R Borzi C Boeri M Fabbri A. Establishment of patient derived xenografts as functional testing of lung cancer aggressiveness. Sci. Rep. (2017) 7:6689. 10.1038/s41598-017-06912-7 28751748 PMC5532258

[96] Zhao C Setrerrahmane S Xu H. Enrichment and characterization of cancer stem cells from a human non-small cell lung cancer cell line. Oncol Rep. (2015) 34:2126–32. 10.3892/or.2015.4163 26239272

[97] Sun F-F Hu Y-H Xiong L-P Tu X-Y Zhao J-H Chen S-S. Enhanced expression of stem cell markers and drug resistance in sphere-forming non-small cell lung cancer cells. Int J Clin Exp Pathol. (2015) 8:6287–300. 26261505 PMC4525839

[98] Yu J Wang S Zhao W Duan J Wang Z Chen H. Mechanistic exploration of cancer stem cell marker voltage-Dependent calcium channel α2δ1 subunit-mediated chemotherapy resistance in small-cell lung cancer. Clin Cancer Res. (2018) 24:2148–58. 10.1158/1078-0432.CCR-17-1932 29437792

[99] Sarvi S Mackinnon AC Avlonitis N Bradley M Rintoul RC Rassl DM. CD133+ cancer stem-like cells in small cell lung cancer are highly tumorigenic and chemoresistant but sensitive to a novel neuropeptide antagonist. Cancer Res. (2014) 74:1554–65. 10.1158/0008-5472.CAN-13-1541 24436149

[100] Meng X Li M Wang X Wang Y Ma D. Both CD133+ and CD133- subpopulations of a549 and h446 cells contain cancer-initiating cells. Cancer Sci. (2009) 100:1040–6. 10.1111/j.1349-7006.2009.01144.x 19385971 PMC11159162

[101] Huang X Huang J Leng D Yang S Yao Q Sun J. Gefitinib-loaded DSPE-PEG2000 nanomicelles with CD133 aptamers target lung cancer stem cells. World J Surg Oncol. (2017) 15:167. 10.1186/s12957-017-1230-4 28854941 PMC5577827

[102] Ma J Zhuang H Zhuang Z Lu Y Xia R Gan L. Development of docetaxel liposome surface modified with CD133 aptamers for lung cancer targeting. Artif Cells Nanomed Biotechnol. (2018) 46:1864–71. 10.1080/21691401.2017.1394874 29082764

[103] Zhou J Sun J Chen H Peng Q. Promoted delivery of salinomycin sodium to lung cancer cells by dual targeting PLGA hybrid nanopartiCLes. Int J Oncol. (2018) 53:1289–300. 10.3892/ijo.2018.4474 30015824

[104] Zhou J Sun M Jin S Fan L Zhu W Sui X. Combined using of paclitaxel and salinomycin active targeting nanostructured lipid carriers against non-small cell lung cancer and cancer stem cells. Drug Deliv. (2019) 26:281–9. 10.1080/10717544.2019.1580799 30880491 PMC6427498

[105] Bertolini G Roz L Perego P Tortoreto M Fontanella E Gatti L. Highly tumorigenic lung cancer CD133+ cells display stem-like features and are spared by cisplatin treatment. Proc Natl Acad Sci USA. (2009) 106:16281–6. 10.1073/pnas.0905653106 19805294 PMC2741477

[106] Wang D Wen G-M Hou W Xia P. The roles of CD133 expression in the patients with non-small cell lung cancer. Cancer Biomark. (2018) 22:385–94. 10.3233/CBM-170835 29758924 PMC13078474

[107] Miyata T Oyama T Yoshimatsu T Higa H Kawano D Sekimura A. The Clinical significance of cancer stem cell markers ALDH1A1 and CD133 in lung adenocarcinoma. Anticancer Res. (2017) 37:2541–7. 10.21873/anticanres.11597 28476825

[108] Su C Xu Y Li X Ren S Zhao C Hou L. Predictive and prognostic effect of CD133 and cancer-testis antigens in stage Ib-IIIA non-small cell lung cancer. Int J Clin Exp Pathol. (2015) 8:5509–18. 26191258 PMC4503129

[109] Qiu Z-X Zhao S Mo X-M Li W-M. Overexpression of PROM1 (CD133) confers poor prognosis in non-small cell lung cancer. Int J Clin Exp Pathol. (2015) 8:6589–95. 26261540 PMC4525874

[110] Wen G-M Mou F-F Hou W Wang D Xia P. Integrative analysis of CD133 mRNA in human cancers based on data mining. Stem Cell Rev Rep. (2019) 15:23–34. 10.1007/s12015-018-9865-2 30430389

[111] Sowa T Menju T Sonobe M Nakanishi T Shikuma K Imamura N. Association between epithelial-mesenchymal transition and cancer stemness and their effect on the prognosis of lung adenocarcinoma. Cancer Med. (2015) 4:1853–62. 10.1002/cam4.556 26471868 PMC5123719

[112] Chen E Zeng Z Bai B Zhu J Song Z. The prognostic value of CSCs biomarker CD133 in NSCLC: a meta-analysis. Oncotarget. (2016) 7:56526–39. 10.18632/oncotarget.10964 27489355 PMC5302932

[113] Zhao M Zhang Y Zhang H Wang S Zhang M Chen X. Hypoxia-induced cell stemness leads to drug resistance and poor prognosis in lung adenocarcinoma. Lung Cancer. (2015) 87:98–106. 10.1016/j.lungcan.2014.11.017 25512094

[114] Hanssen A Wagner J Gorges TM Taenzer A Uzunoglu FG Driemel C. Characterization of different CTC subpopulations in non-small cell lung cancer. Sci. Rep. (2016) 6:28010. 10.1038/srep28010 27302574 PMC4908396

[115] Jahchan NS Lim JS Bola B Morris K Seitz G Tran KQ. Identification and targeting of long-term tumor-Propagating cells in small cell lung cancer. Cell Rep. (2016) 16:644–56. 10.1016/j.celrep.2016.06.021 27373157 PMC4956576

[116] Chen L Peng M Li N Song Q Yao Y Xu B. Combined use of epCAM and FRα enables the high-efficiency capture of circulating tumor cells in non-small cell lung cancer. Sci. Rep. (2018) 8:1188. 10.1038/s41598-018-19391-1 29352248 PMC5775318

[117] Wit S de Rossi E Weber S Tamminga M Manicone M Swennenhuis JF. Single tube liquid biopsy for advanced non-small cell lung cancer. Int J Cancer. (2019) 144:3127–37. 10.1002/ijc.32056 30536653

[118] Zamay GS Kolovskaya OS Ivanchenko TI Zamay TN Veprintsev DV Grigorieva VL. Development of DNA aptamers to native EpCAM for isolation of lung circulating tumor cells from human blood. Cancers. (2019) 11:351. 10.3390/cancers11030351 30871104 PMC6468627

[119] Rud AK Boye K Fodstad Ø Juell S Jørgensen LH Solberg S. Detection of disseminated tumor cells in lymph nodes from patients with early stage non-small cell lung cancer. Diagn Pathol. (2016) 11:50. 10.1186/s13000-016-0504-4 27316334 PMC4912762

[120] Gao W Huang T Yuan H Yang J Jin Q Jia C. Highly sensitive detection and mutational analysis of lung cancer circulating tumor cells using integrated combined immunomagnetic beads with a droplet digital PCR chip. Talanta. (2018) 185:229–36. 10.1016/j.talanta.2018.03.083 29759193

[121] Alibolandi M Ramezani M Abnous K Sadeghi F Atyabi F Asouri M. *In vitro* and *in vivo* evaluation of therapy targeting epithelial-cell adhesion-molecule aptamers for non-small cell lung cancer. J Control Release. (2015) 209:88–100. 10.1016/j.jconrel.2015.04.026 25912964

[122] Thompson JC Fan R Black T Yu GH Savitch SL Chien A. Measurement and immunophenotyping of pleural fluid epCAM-positive cells and CLusters for the management of non-small cell lung cancer patients. Lung Cancer. (2019) 127:25–33. 10.1016/j.lungcan.2018.11.020 30642547 PMC6657687

[123] Zhou N Wang H Liu H Xue H Lin F Meng X. MTA1-upregulated epCAM is associated with metastatic behaviors and poor prognosis in lung cancer. J Exp Clin Cancer Res. (2015) 34:157. 10.1186/s13046-015-0263-1 26698569 PMC4690245

[124] Wit S de van Dalum G Lenferink AT Tibbe AG Hiltermann TJ Groen HJ. The detection of epCAM(+) and epCAM(-) circulating tumor cells. Sci. Rep. (2015) 5:12270. 10.1038/srep12270 26184843 PMC4505332

[125] Gao F Zhou B Xu J-C Gao X Li S-X Zhu G-C. The role of LGR5 and ALDH1A1 in non-small cell lung cancer: cancer progression and prognosis. Biochem Biophys Res Commun. (2015) 462:91–8. 10.1016/j.bbrc.2015.04.029 25881507

[126] Koh YW Han J-H Haam S Jung J. ALDH1 expression correlates with an epithelial-like phenotype and favorable prognosis in lung adenocarcinoma: a study based on immunohistochemistry and mRNA expression data. J Cancer Res Clin Oncol. (2019) 145:1427–36. 10.1007/s00432-019-02906-2 30923946 PMC11810340

[127] Codony-Servat J Codony-Servat C Cardona AF Giménez-Capitán A Drozdowskyj A Berenguer J. Cancer stem cell biomarkers in EGFR-mutation-positive non-small-cell lung cancer. Clin Lung Cancer. (2019) 20:167–77. 10.1016/j.CLlc.2019.02.005 30885551

[128] Jiang F Qiu Q Khanna A Todd NW Deepak J Xing L. Aldehyde dehydrogenase 1 is a tumor stem cell-associated marker in lung cancer. Mol Cancer Res. (2009) 7:330–8. 10.1158/1541-7786.MCR-08-0393 19276181 PMC4255559

[129] Masciale V Grisendi G Banchelli F D'Amico R Maiorana A Sighinolfi P. Isolation and identification of cancer stem-like cells in adenocarcinoma and squamous cell carcinoma of the lung: a Pilot study. Front. Oncol. (2019) 9:1394. 10.3389/fonc.2019.01394 31921651 PMC6930193

[130] Codony-Servat J Verlicchi A Rosell R. Cancer stem cells in small cell lung cancer. Transl Lung Cancer Res. (2016) 5:16–25. 10.3978/j.issn.2218-6751.2016.01.01 26958490 PMC4758966

[131] Rossi A Voigtlaender M Klose H Schlüter H Schön G Loges S. High aldehyde dehydrogenase levels are detectable in the serum of patients with lung cancer and may be exploited as screening biomarkers. J Oncol. (2019) 2019:8970645. 10.1155/2019/8970645 31534455 PMC6724438

[132] Liu X Wang L Cui W Yuan X Lin L Cao Q. Targeting ALDH1A1 by disulfiram/copper complex inhibits non-small cell lung cancer recurrence driven by ALDH-positive cancer stem cells. Oncotarget. (2016) 7:58516–30. 10.18632/oncotarget.11305 27542268 PMC5295448

[133] Kang JH Lee S-H Lee J-S Nam B Seong TW Son J. Aldehyde dehydrogenase inhibition combined with phenformin treatment reversed nSCLC through aTP depletion. Oncotarget. (2016) 7:49397–410. 10.18632/oncotarget.10354 27384481 PMC5226516

[134] Wang N-N Wang L-H Li Y Fu S-Y Xue X Jia L-N. Targeting ALDH2 with disulfiram/copper reverses the resistance of cancer cells to microtubule inhibitors. Exp Cell Res. (2018) 362:72–82. 10.1016/j.yexcr.2017.11.004 29155365

[135] Morise M Hishida T Takahashi A Yoshida J Ohe Y Nagai K. CLinicopathological significance of cancer stem-like cell markers in high-grade neuroendocrine carcinoma of the lung. J Cancer Res Clin Oncol. (2015) 141:2121–30. 10.1007/s00432-015-1985-3 25963795 PMC11823834

[136] Li S-J Huang J Zhou X-D Zhang W-B Lai Y-T Che G-W. CLinicopathological and prognostic significance of oct-4 expression in patients with non-small cell lung cancer: a systematic review and meta-analysis. J Thorac Dis. (2016) 8:1587–600. 10.21037/jtd.2016.06.01 27499947 PMC4958804

[137] Kouros-Mehr H Bechis SK Slorach EM Littlepage LE Egeblad M Ewald AJ. GATA-3 links tumor differentiation and dissemination in a luminal breast cancer model. Cancer Cell. (2008) 13:141–52. 10.1016/j.ccr.2008.01.011 18242514 PMC2262951

[138] Barnawi R Al-Khaldi S Colak D Tulbah A Al-Tweigeri T Fallatah M. β1 Integrin is essential for fascin-mediated breast cancer stem cell function and disease progression. Int J Cancer. (2019) 145:830–41. 10.1002/ijc.32183 30719702 PMC6593770

[139] Da Cruz Paula A Leitão C Marques O Rosa AM Santos AH Rêma A. Molecular characterization of CD44+/CD24-/Ck+/CD45- cells in benign and malignant breast lesions. Virchows Arch. (2017) 470:311–22. 10.1007/s00428-017-2068-4 28116522

[140] Nam K Oh S Lee KM Yoo SA Shin I. CD44 regulates cell proliferation, migration, and invasion via modulation of c-Src transcription in human breast cancer cells. Cell Signal. (2015) 27:1882–94. 10.1016/j.cellsig.2015.05.002 25979842

[141] Zhang H Brown RL Wei Y Zhao P Liu S Liu X. CD44 splice isoform switching determines breast cancer stem cell state. Genes Dev. (2019) 33:166–79. 10.1101/gad.319889.118 30692202 PMC6362815

[142] Zhang L Xu L Zhang F Vlashi E. Doxycycline inhibits the cancer stem cell phenotype and epithelial-to-mesenchymal transition in breast cancer. Cell Cycle. (2017) 16:737–45. 10.1080/15384101.2016.1241929 27753527 PMC5405729

[143] Ge G Zhou C Ren Y Tang X Wang K Zhang W. Enhanced SLC34A2 in breast cancer stem cell-like cells induces chemotherapeutic resistance to doxorubicin via SLC34A2-Bmi1-ABCC5 signaling. Tumour Biol. (2016) 37:5049–62. 10.1007/s13277-015-4226-0 26546432

[144] Colacino JA Azizi E Brooks MD Harouaka R Fouladdel S MCDermott SP. Heterogeneity of human breast stem and progenitor cells as revealed by transcriptional profiling. Stem Cell Reports. (2018) 10:1596–609. 10.1016/j.stemcr.2018.03.001 29606612 PMC5995162

[145] Hu J Li G Zhang P Zhuang X Hu G. A CD44v+ subpopulation of breast cancer stem-like cells with enhanced lung metastasis capacity. Cell Death Dis. (2017) 8:e2679. 10.1038/cddis.2017.72 28300837 PMC5386565

[146] Ji P Zhang Y Wang S-J Ge H-L Zhao G-P Xu Y-C. CD44hiCD24lo mammosphere-forming cells from primary breast cancer display resistance to multiple chemotherapeutic drugs. Oncol Rep. (2016) 35:3293–302. 10.3892/or.2016.4739 27109463

[147] Li W Ma H Zhang J Zhu L Wang C Yang Y. Unraveling the roles of CD44/CD24 and ALDH1 as cancer stem cell markers in tumorigenesis and metastasis. Sci. Rep. (2017) 7:13856. 10.1038/s41598-017-14364-2 29062075 PMC5653849

[148] Louhichi T Ziadi S Saad H Dhiab MB Mestiri S Trimeche M. CLinicopathological significance of cancer stem cell markers CD44 and ALDH1 expression in breast cancer. Breast Cancer. (2018) 25:698–705. 10.1007/s12282-018-0875-3 29845398

[149] Cho Y Lee H-W Kang H-G Kim H-Y Kim S-J Chun K-H. Cleaved CD44 intracellular domain supports activation of stemness factors and promotes tumorigenesis of breast cancer. Oncotarget. (2015) 6:8709–21. 10.18632/oncotarget.3325 25909162 PMC4496178

[150] Jin J Krishnamachary B Mironchik Y Kobayashi H Bhujwalla ZM. Phototheranostics of CD44-positive cell populations in triple negative breast cancer. Sci. Rep. (2016) 6:27871. 10.1038/srep27871 27302409 PMC4908597

[151] Qiu Y Zhou B Yang X Long D Hao Y Yang P. Novel single-cell analysis platform based on a solid-State zinc-Coadsorbed carbon quantum dots electrochemiluminescence probe for the evaluation of CD44 expression on breast cancer cells. ACS Appl Mater Interfaces. (2017) 9:16848–56. 10.1021/acsami.7b02793 28481500

[152] Wang Z Sau S Alsaab HO Iyer AK. CD44 directed nanomicellar payload delivery platform for selective anticancer effect and tumor specific imaging of triple negative breast cancer. Nanomedicine. (2018) 14:1441–54. 10.1016/j.nano.2018.04.004 29678787 PMC6192858

[153] Yaghjyan L Stoll E Ghosh K Scott CG Jensen MR Brandt KR. Tissue-based associations of mammographic breast density with breast stem cell markers. Breast Cancer Res. (2017) 19:100. 10.1186/s13058-017-0889-3 28851411 PMC5576318

[154] Yang R-M Fu C-P Fang J-Z Xu X-D Wei X-H Tang W-J. Hyaluronan-modified superparamagnetic iron oxide nanopartiCLes for bimodal breast cancer imaging and photothermal therapy. Int J Nanomedicine. (2017) 12:197–206. 10.2147/IJN.S121249 28096667 PMC5214799

[155] MunTIMadugu E Kumar R Saladi S Rafeeqi TA Khan W. CD44 targeted chemotherapy for co-eradication of breast cancer stem cells and cancer cells using polymeric nanopartiCLes of salinomycin and paclitaxel. Colloids Surf B Biointerfaces. (2016) 143:532–46. 10.1016/j.colsurfb.2016.03.075 27045981

[156] Agrawal S Dwivedi M Ahmad H Chadchan SB Arya A Sikandar R. CD44 targeting hyaluronic acid coated lapatinib nanocrystals foster the efficacy against triple-negative breast cancer. Nanomedicine. (2018) 14:327–37. 10.1016/j.nano.2017.10.010 29129754

[157] Aguirre-Alvarado C Segura-Cabrera A Velázquez-Quesada I Hernández-Esquivel MA García-Pérez CA Guerrero-Rodríguez SL. Virtual screening-driven repositioning of etoposide as CD44 antagonist in breast cancer cells. Oncotarget. (2016) 7:23772–84. 10.18632/oncotarget.8180 27009862 PMC5029662

[158] Chen J He H Deng C Yin L Zhong Z. Saporin-loaded CD44 and EGFR dual-targeted nanogels for potent inhibition of metastatic breast cancer *in vivo*. Int J Pharm. (2019) 560:57–64. 10.1016/j.ijpharm.2019.01.040 30699364

[159] Chen J Ouyang J Chen Q Deng C Meng F Zhang J. EGFR and CD44 dual-Targeted multifunctional hyaluronic acid nanogels boost protein delivery to ovarian and breast cancers *in vitro* and *in vivo*. ACS Appl Mater Interfaces. (2017) 9:24140–7. 10.1021/acsami.7b06879 28675028

[160] Fan W Wang X Ding B Cai H Wang X Fan Y. Thioaptamer-conjugated CD44-targeted delivery system for the treatment of breast cancer *in vitro* and *in vivo*. J Drug Target. (2016) 24:359–71. 10.3109/1061186X.2015.1077850 26299192

[161] Fan Y Wang Q Lin G Shi Y Gu Z Ding T. Combination of using prodrug-modified cationic liposome nanocomplexes and a potentiating strategy via targeted co-delivery of gemcitabine and docetaxel for CD44-overexpressed triple negative breast cancer therapy. Acta Biomater. (2017) 62:257–72. 10.1016/j.actbio.2017.08.034 28859899

[162] Fu W Sun H Zhao Y Chen M Yang L Yang X. Targeted delivery of CD44s-siRNA by ScFv overcomes *de novo* resistance to cetuximab in triple negative breast cancer. Mol Immunol. (2018) 99:124–33. 10.1016/j.molimm.2018.05.010 29777999

[163] Han N-K Shin DH Kim JS Weon KY Jang C-Y Kim J-S. Hyaluronan-conjugated liposomes encapsulating gemcitabine for breast cancer stem cells. Int J Nanomedicine. (2016) 11:1413–25. 10.2147/IJN.S95850 27103799 PMC4827594

[164] Liang D-S Zhang W-J Wang A-T Su H-T Zhong H-J Qi X-R. Treating metastatic triple negative breast cancer with CD44/neuropilin dual molecular targets of multifunctional nanopartiCLes. Biomaterials. (2017) 137:23–36. 10.1016/j.biomaterials.2017.05.022 28528300

[165] Xie X Huang X Tang H Ye F Yang L Guo X. Diallyl disulfide inhibits breast cancer stem cell progression and glucose metabolism by targeting CD44/PKM2/AMPK signaling. Curr Cancer Drug Targets. (2018) 18:592–9. 10.2174/1568009617666171024165657 29110616

[166] Yang C He Y Zhang H Liu Y Wang W Du Y. Selective killing of breast cancer cells expressing activated CD44 using CD44 ligand-coated nanopartiCLes *in vitro* and *in vivo*. Oncotarget. (2015) 6:15283–96. 10.18632/oncotarget.3681 25909172 PMC4558151

[167] McFarlane S Coulter JA Tibbits P O'Grady A McFarlane C Montgomery N CD44 increases the efficiency of distant metastasis of breast cancer. Oncotarget. (2015) 6:11465–76. 10.18632/oncotarget.3410 25888636 PMC4484469

[168] Rico MJ Perroud HA Herrera C Alasino CM Roggero EA Pezzotto SM. Putative biomarkers of response to treatment in breast cancer patients: a pilot assay. Cancer Invest. (2017) 35:377–85. 10.1080/07357907.2017.1309545 28426268

[169] Sanmartín E Ortiz-Martínez F Pomares-Navarro E García-Martínez A Rodrigo-Baños M García-Escolano M. CD44 induces FOXP3 expression and is related with favorable outcome in breast carcinoma. Virchows Arch. (2017) 470:81–90. 10.1007/s00428-016-2045-3 27885422

[170] Seo AN Lee HJ Kim EJ Jang MH Kim YJ Kim JH. Expression of breast cancer stem cell markers as predictors of prognosis and response to trastuzumab in HER2-positive breast cancer. Br J Cancer. (2016) 114:1109–16. 10.1038/bjc.2016.101 27115469 PMC4865964

[171] Da Cruz Paula A Marques O Sampaio R Rosa A Garcia J Rêma A. Characterization of CD44+ALDH1+Ki-67- cells in non-malignant and neoplastic lesions of the breast. Anticancer Res. (2016) 36:4629–38. 10.21873/anticanres.11013 27630305

[172] Rabinovich I Sebastião AP Lima RS Urban CD Junior ES Anselmi KF. Cancer stem cell markers ALDH1 and CD44+/CD24- phenotype and their prognosis impact in invasive ductal carcinoma. Eur J Histochem. (2018) 62:2943. 10.4081/ejh.2018.2943 30362671 PMC6240790

[173] Yaghjyan L Esnakula AK Scott CG Wijayabahu AT Jensen MR Vachon CM. Associations of mammographic breast density with breast stem cell marker-defined breast cancer subtypes. Cancer Causes Control. (2019) 30:1103–11. 10.1007/s10552-019-01207-w 31352658 PMC7433400

[174] Gómez-Miragaya J Palafox M Paré L Yoldi G Ferrer I Vila S. Resistance to taxanes in triple-negative breast cancer associates with the dynamics of a CD49f+ tumor-Initiating population. Stem Cell Reports. (2017) 8:1392–407. 10.1016/j.stemcr.2017.03.026 28457887 PMC5425727

[175] Ye F Zhong X Qiu Y Yang L Wei B Zhang Z CD49f can act as a biomarker for local or distant recurrence in breast cancer. J Breast Cancer. (2017) 20:142–9. 10.4048/jbc.2017.20.2.142 28690650 PMC5500397

[176] Gomez-Miragaya J González-Suárez E. Tumor-initiating CD49f cells are a hallmark of chemoresistant triple negative breast cancer. Mol Cell Oncol. (2017) 4:e1338208. 10.1080/23723556.2017.1338208 28868349 PMC5540214

[177] Krebsbach PH Villa-Diaz LG. The role of integrin α6 (CD49f) in stem cells: more than a conserved biomarker. Stem Cells Dev. (2017) 26:1090–9. 10.1089/scd.2016.0319 28494695 PMC5563922

[178] Hu T Zhou R Zhao Y Wu G. Integrin α6/Akt/Erk signaling is essential for human breast cancer resistance to radiotherapy. Sci. Rep. (2016) 6:33376. 10.1038/srep33376 27624978 PMC5022055

[179] Ye F Qiu Y Li L Yang L Cheng F Zhang H. The presence of epCAM(-)/CD49f(+) cells in breast cancer is associated with a poor clinical outcome. J Breast Cancer. (2015) 18:242–8. 10.4048/jbc.2015.18.3.242 26472974 PMC4600688

[180] Yeo SK Wen J Chen S Guan J-L. Autophagy differentially regulates distinct breast cancer stem-like cells in murine models via EGFR/Stat3 and Tgfβ/Smad signaling. Cancer Res. (2016) 76:3397–410. 10.1158/0008-5472.CAN-15-2946 27197172 PMC4990205

[181] Liu L Yin B Yi Z Liu X Hu Z Gao W. Breast cancer stem cells characterized by CD70 expression preferentially metastasize to the lungs. Breast Cancer. (2018) 25:706–16. 10.1007/s12282-018-0880-6 29948958

[182] Wang X Liu Y Zhou K Zhang G Wang F Ren J. Isolation and characterization of CD105+/CD90+ subpopulation in breast cancer mDA-MB-231 cell line. Int J Clin Exp Pathol. (2015) 8:5105–12. 26191205 PMC4503077

[183] Sansone P Berishaj M Rajasekhar VK Ceccarelli C Chang Q Strillacci A. Evolution of cancer stem-like cells in endocrine-resistant metastatic breast cancers is mediated by stromal microvesiCLes. Cancer Res. (2017) 77:1927–41. 10.1158/0008-5472.CAN-16-2129 28202520 PMC5392366

[184] Brugnoli F Grassilli S Al-Qassab Y Capitani S Bertagnolo V. CD133 in breast cancer cells: more than a stem cell marker. J Oncol. (2019) 2019:7512632. 10.1155/2019/7512632 31636668 PMC6766124

[185] Zhang D Sun B Zhao X Ma Y Ji R Gu Q. Twist1 expression induced by sunitinib accelerates tumor cell vasculogenic mimicry by increasing the population of CD133+ cells in triple-negative breast cancer. Mol Cancer. (2014) 13:207. 10.1186/1476-4598-13-207 25200065 PMC4168051

[186] Brugnoli F Grassilli S Lanuti P Marchisio M Al-Qassab Y Vezzali F. Up-modulation of pLC-β2 reduces the number and malignancy of triple-negative breast tumor cells with a CD133+/EpCAM+ phenotype: a promising target for preventing progression of TNBC. BMC Cancer. (2017) 17:617. 10.1186/s12885-017-3592-y 28870198 PMC5584040

[187] Sansone P Ceccarelli C Berishaj M Chang Q Rajasekhar VK PeRNA F. Self-renewal of CD133(hi) cells by IL6/Notch3 signalling regulates endocrine resistance in metastatic breast cancer. Nat Commun. (2016) 7:10442. 10.1038/ncomms10442 26858125 PMC4748123

[188] Swaminathan SK Roger E Toti U Niu L Ohlfest JR Panyam J. CD133-targeted paCLitaxel delivery inhibits local tumor recurrence in a mouse model of breast cancer. J Control Release. (2013) 171:280–7. 10.1016/j.jconrel.2013.07.014 23871962

[189] Brugnoli F Grassilli S Piazzi M Palomba M Nika E Bavelloni A. In triple negative breast tumor cells, pLC-β2 promotes the conversion of CD133high to CD133low phenotype and reduces the CD133-related invasiveness. Mol Cancer. (2013) 12:165. 10.1186/1476-4598-12-165 24330829 PMC3866498

[190] Latorre E Carelli S Raimondi I D'Agostino V Castiglioni I Zucal C. The ribonucleic complex HuR-MALAT1 represses CD133 expression and suppresses epithelial-Mesenchymal transition in breast cancer. Cancer Res. (2016) 76:2626–36. 10.1158/0008-5472.CAN-15-2018 27197265

[191] Bock C Kuhn C Ditsch N Krebold R Heublein S Mayr D. Strong correlation between n-cadherin and CD133 in breast cancer: role of both markers in metastatic events. J Cancer Res Clin Oncol. (2014) 140:1873–81. 10.1007/s00432-014-1750-z 24962344 PMC11824022

[192] Nozaki Y Tamori S Inada M Katayama R Nakane H Minamishima O. Correlation between c-Met and ALDH1 contributes to the survival and tumor-sphere formation of ALDH1 positive breast cancer stem cells and predicts poor Clinical outcome in breast cancer. Genes Cancer. (2017) 8:628–39. 10.18632/genesandcancer.148 28966724 PMC5620008

[193] Joseph C Arshad M Kurozomi S Althobiti M Miligy IM Al-Izzi S. Overexpression of the cancer stem cell marker CD133 confers a poor prognosis in invasive breast cancer. Breast Cancer Res Treat. (2019) 174:387–99. 10.1007/s10549-018-05085-9 30554343

[194] Zhang M Tsimelzon A Chang C-H Fan C Wolff A Perou CM. Intratumoral heterogeneity in a trp53-null mouse model of human breast cancer. Cancer Discov. (2015) 5:520–33. 10.1158/2159-8290.CD-14-1101 25735774 PMC4420701

[195] Yang L Tang H Kong Y Xie X Chen J Song C. LGR5 promotes breast cancer progression and maintains stem-Like cells through activation of wnt/β-Catenin signaling. Stem Cells. (2015) 33:2913–24. 10.1002/stem.2083 26086949

[196] Wang D Cai C Dong X Yu QC Zhang X-O Yang L. Identification of multipotent mammary stem cells by protein C receptor expression. Nature. (2015) 517:81–4. 10.1038/nature13851 25327250

[197] Ginestier C Hur MH Charafe-Jauffret E Monville F Dutcher J Brown M. ALDH1 is a marker of normal and malignant human mammary stem cells and a predictor of poor clinical outcome. Cell Stem Cell. (2007) 1:555–67. 10.1016/j.stem.2007.08.014 18371393 PMC2423808

[198] Kim R-J Park J-R Roh K-J Choi A-R Kim S-R Kim P-H. High aldehyde dehydrogenase activity enhances stem cell features in breast cancer cells by activating hypoxia-inducible factor-2α. Cancer Lett. (2013) 333:18–31. 10.1016/j.canlet.2012.11.026 23174107

[199] Rodriguez-Torres M Allan AL. Aldehyde dehydrogenase as a marker and functional mediator of metastasis in solid tumors. Clin Exp Metastasis. (2016) 33:97–113. 10.1007/s10585-015-9755-9 26445849 PMC4740561

[200] Tomita H Tanaka K Tanaka T Hara A. Aldehyde dehydrogenase 1A1 in stem cells and cancer. Oncotarget. (2016) 7:11018–32. 10.18632/oncotarget.6920 26783961 PMC4905455

[201] Liu P Kumar IS Brown S Kannappan V Tawari PE Tang JZ Disulfiram targets cancer stem-like cells and reverses resistance and cross-resistance in acquired paCLitaxel-resistant triple-negative breast cancer cells. Br J Cancer. (2013) 109:1876–85. 10.1038/bjc.2013.534 24008666 PMC3790184

[202] Matsunaga N Ogino T Hara Y Tanaka T Koyanagi S Ohdo S. Optimized dosing schedule based on circadian dynamics of mouse breast cancer stem cells improves the antitumor effects of aldehyde dehydrogenase inhibitor. Cancer Res. (2018) 78:3698–708. 10.1158/0008-5472.CAN-17-4034 29735553

[203] Charafe-Jauffret E Ginestier C Iovino F Tarpin C Diebel M Esterni B. Aldehyde dehydrogenase 1-positive cancer stem cells mediate metastasis and poor Clinical outcome in inflammatory breast cancer. Clin Cancer Res. (2010) 16:45–55. 10.1158/1078-0432.CCR-09-1630 20028757 PMC2874875

[204] Liu Y Lv D-l Duan J-j Xu S-l Zhang J-f Yang X-j. ALDH1A1 expression correlates with CLinicopathologic features and poor prognosis of breast cancer patients: a systematic review and meta-analysis. BMC Cancer. (2014) 14:444. 10.1186/1471-2407-14-444 24938375 PMC4070403

[205] Marcato P Dean CA Liu R-Z Coyle KM Bydoun M Wallace M. Aldehyde dehydrogenase 1A3 influences breast cancer progression via differential retinoic acid signaling. Mol Oncol. (2015) 9:17–31. 10.1016/j.molonc.2014.07.010 25106087 PMC5528683

[206] Khoury T Ademuyiwa FO Chandrasekhar R Chandraseekhar R Jabbour M Deleo A. Aldehyde dehydrogenase 1A1 expression in breast cancer is associated with stage, triple negativity, and outcome to neoadjuvant chemotherapy. Mod Pathol. (2012) 25:388–97. 10.1038/modpathol.2011.172 22080062 PMC3426278

[207] Woodward WA Krishnamurthy S Lodhi A Xiao L Gong Y Cristofanilli M Aldehyde dehydrogenase1 immunohistochemical staining in primary breast cancer cells independently predicted overall survival but did not correlate with the presence of circulating or disseminated tumors cells. J Cancer. (2014) 5:360–7. 10.7150/jca.7885 24799954 PMC4007524

[208] Zhong Y Shen S Zhou Y Mao F Guan J Lin Y. ALDH1 is a better clinical indicator for relapse of invasive ductal breast cancer than the CD44+/CD24- phenotype. Med Oncol. (2014) 31:864. 10.1007/s12032-014-0864-0 24519209

[209] Tan EY Thike AA Tan PH. ALDH1 expression is enriched in breast cancers arising in young women but does not predict outcome. Br J Cancer. (2013) 109:109–13. 10.1038/bjc.2013.297 23787917 PMC3708574

[210] Collina F Di Bonito M Li Bergolis V Laurentiis M de Vitagliano C Cerrone M. Prognostic value of cancer stem cells markers in triple-negative breast cancer. Biomed Res Int. (2015) 2015:158682. 10.1155/2015/158682 26504780 PMC4609334

[211] Srinivasan M Bharali DJ Sudha T Khedr M Guest I Sell S. Downregulation of BMI1 in breast cancer stem cells suppresses tumor growth and proliferation. Oncotarget. (2017) 8:38731–42. 10.18632/oncotarget.16317 28418883 PMC5503567

[212] Kim S-H Singh SV. The role of polycomb group protein BMI-1 and notch4 in breast cancer stem cell inhibition by benzyl isothiocyanate. Breast Cancer Res Treat. (2015) 149:681–92. 10.1007/s10549-015-3279-5 25663545 PMC4329083

[213] Yuan W Yuan Y Zhang T Wu S. Role of BMI-1 in regulation of ionizing irradiation-induced epithelial-mesenchymal transition and migration of breast cancer cells. PLoS ONE. (2015) 10:e0118799. 10.1371/jouRNAl.pone.0118799 25734775 PMC4348174

[214] Yan Y Wang Y Zhao P Ma W Hu Z Zhang K. BMI-1 promotes self-Renewal of radio- and temozolomide (TMZ)-Resistant breast cancer cells. Reprod Sci. (2017) 24:1620–9. 10.1177/1933719117697255 28270035

[215] Gong X-F Yu A-L Tang J Wang C-L He J-R Chen G-Q. MicroRNA-630 inhibits breast cancer progression by directly targeting BMI1. Exp Cell Res. (2018) 362:378–85. 10.1016/j.yexcr.2017.11.039 29208462

[216] Griffith J Andrade D Mehta M Berry W Benbrook DM Aravindan N. Silencing BMI1 radiosensitizes human breast cancer cells by inducing DNA damage and autophagy. Oncol Rep. (2017) 37:2382–90. 10.3892/or.2017.5478 28260023 PMC5367353

[217] Ojo D Lin X Wu Y Cockburn J Bane A Tang D. Polycomb complex protein BMI1 confers resistance to tamoxifen in estrogen receptor positive breast cancer. Cancer Lett. (2018) 426:4–13. 10.1016/j.canlet.2018.03.048 29626519

[218] Elango R Vishnubalaji R Manikandan M Binhamdan SI Siyal A-A Alshawakir YA. Concurrent targeting of BMI1 and CDK4/6 abrogates tumor growth *in vitro* and *in vivo*. Sci. Rep. (2019) 9:13696. 10.1038/s41598-019-50140-0 31548560 PMC6757061

[219] Janaki Ramaiah M Vaishnave S. BMI1 and PTEN are key determinants of breast cancer therapy: a plausible therapeutic target in breast cancer. Gene. (2018) 678:302–11. 10.1016/j.gene.2018.08.022 30096458

[220] Wang D Lu P Zhang H Luo M Zhang X Wei X. Oct-4 and nanog promote the epithelial-mesenchymal transition of breast cancer stem cells and are associated with poor prognosis in breast cancer patients. Oncotarget. (2014) 5:10803–15. 10.18632/oncotarget.2506 25301732 PMC4279411

[221] Yang F Zhang J Yang H. OCT4, SOX2, and NANOG positive expression correlates with poor differentiation, advanced disease stages, and worse overall survival in HER2+ breast cancer patients. Onco Targets Ther. (2018) 11:7873–81. 10.2147/OTT.S173522 30464534 PMC6228048

[222] D'Angelo RC Ouzounova M Davis A Choi D Tchuenkam SM Kim G. Notch reporter activity in breast cancer cell lines identifies a subset of cells with stem cell activity. Mol Cancer Ther. (2015) 14:779–87. 10.1158/1535-7163.MCT-14-0228 25673823 PMC4456218

[223] Rodilla V Dasti A Huyghe M Lafkas D Laurent C Reyal F. Luminal progenitors restrict their lineage potential during mammary gland development. PLoS Biol. (2015) 13:e1002069. 10.1371/jouRNAl.pbio.1002069 25688859 PMC4331521

[224] Mamaeva V Niemi R Beck M Özliseli E Desai D Landor S. Inhibiting notch activity in breast cancer stem cells by glucose functionalized nanoparticles carrying γ-secretase inhibitors. Mol Ther. (2016) 24:926–36. 10.1038/mt.2016.42 26916284 PMC4881775

[225] Anjanappa M Hao Y Simpson ER Bhat-Nakshatri P Nelson JB Tersey SA. A system for detecting high impact-low frequency mutations in primary tumors and metastases. Oncogene. (2018) 37:185–96. 10.1038/onc.2017.322 28892047 PMC5764779

[226] Baker A Wyatt D Bocchetta M Li J Filipovic A Green A. Notch-1-PTEN-ERK1/2 signaling axis promotes hER2+ breast cancer cell proliferation and stem cell survival. Oncogene. (2018) 37:4489–504. 10.1038/s41388-018-0251-y 29743588 PMC9115842

[227] Diluvio G Del Gaudio F Giuli MV Franciosa G Giuliani E Palermo R. NOTCH3 inactivation increases triple negative breast cancer sensitivity to gefitinib by promoting eGFR tyrosine dephosphorylation and its intracellular arrest. Oncogenesis. (2018) 7:42. 10.1038/s41389-018-0051-9 29795369 PMC5968025

[228] Ran Y Hossain F Pannuti A Lessard CB Ladd GZ Jung J. γ-Secretase inhibitors in cancer clinical trials are pharmacologically and functionally distinct. Embo Mol Med. (2017) 9:950–66. 10.15252/emmm.201607265 28539479 PMC5494507

[229] Sizemore GM Balakrishnan S Hammer AM Thies KA Trimboli AJ Wallace JA Stromal pTEN inhibits the expansion of mammary epithelial stem cells through jagged-1. Oncogene. (2017) 36:2297–308. 10.1038/onc.2016.383 27797378 PMC5398932

[230] Bhola NE Jansen VM Koch JP Li H Formisano L Williams JA. Treatment of triple-Negative breast cancer with TORC1/2 inhibitors sustains a drug-Resistant and notch-dependent cancer stem cell population. Cancer Res. (2016) 76:440–52. 10.1158/0008-5472.CAN-15-1640-T 26676751 PMC4715956

[231] Leontovich AA Jalalirad M Salisbury JL Mills L Haddox C Schroeder M. NOTCH3 expression is linked to breast cancer seeding and distant metastasis. Breast Cancer Res. (2018) 20:105. 10.1186/s13058-018-1020-0 30180881 PMC6123953

[232] Dou X-W Liang Y-K Lin H-Y Wei X-L Zhang Y-Q Bai J-W. Notch3 maintains luminal phenotype and suppresses tumorigenesis and metastasis of breast cancer via trans-activating estrogen receptor-α. Theranostics. (2017) 7:4041–56. 10.7150/thno.19989 29109797 PMC5667424

[233] Wieland E Rodriguez-Vita J Liebler SS Mogler C Moll I Herberich SE. Endothelial notch1 activity facilitates metastasis. Cancer Cell. (2017) 31:355–67. 10.1016/j.ccell.2017.01.007 28238683

[234] Gu X Lu C He D Lu Y Jin J Liu D. Notch3 negatively regulates chemoresistance in breast cancers. Tumour Biol. (2016) 37. 10.1007/s13277-016-5412-4 27743379

[235] Janghorban M Xin L Rosen JM Zhang XH-F Notch signaling as a regulator of the tumor immune response: to target or not to target? Front Immunol. (2018) 9:1649 10.3389/fimmu.2018.01649 30061899 PMC6055003

[236] Siddharth S Goutam K Das S Nayak A Nayak D Sethy C. Nectin-4 is a breast cancer stem cell marker that induces wNT/β-catenin signaling via pi3k/Akt axis. Int J Biochem Cell Biol. (2017) 89:85–94. 10.1016/j.biocel.2017.06.007 28600142

[237] Jang G-B Hong I-S Kim R-J Lee S-Y Park S-J Lee E-S. Wnt/β-catenin small-molecule inhibitor CWP232228 preferentially inhibits the growth of breast cancer stem-like cells. Cancer Res. (2015) 75:1691–702. 10.1158/0008-5472.CAN-14-2041 25660951

[238] Zhang C Li C He F Cai Y Yang H. Identification of CD44+CD24+ gastric cancer stem cells. J Cancer Res Clin Oncol. (2011) 137:1679–86. 10.1007/s00432-011-1038-5 21882047 PMC11828146

[239] Brungs D Aghmesheh M Vine KL Becker TM Carolan MG Ranson M. Gastric cancer stem cells: evidence, potential markers, and clinical implications. J Gastroenterol. (2016) 51:313–26. 10.1007/s00535-015-1125-5 26428661

[240] Li K Dan Z Nie Y-Q. Gastric cancer stem cells in gastric carcinogenesis, progression, prevention and treatment. World J Gastroenterol. (2014) 20:5420–6. 10.3748/wjg.v20.i18.5420 24833872 PMC4017057

[241] Jiao X-L Zhao C Niu M Chen D. Downregulation of CD24 inhibits invasive growth, facilitates apoptosis and enhances chemosensitivity in gastric cancer AGS cells. Eur Rev Med Pharmacol Sci. (2013) 17:1709–15. 23852892

[242] Fujikuni N Yamamoto H Tanabe K Naito Y Sakamoto N Tanaka Y. Hypoxia-mediated CD24 expression is correlated with gastric cancer aggressiveness by promoting cell migration and invasion. Cancer Sci. (2014) 105:1411–20. 10.1111/cas.12522 25174257 PMC4462374

[243] Wu J-X Zhao Y-Y Wu X An H-X. CLinicopathological and prognostic significance of CD24 overexpression in patients with gastric cancer: a meta-analysis. PLoS ONE. (2014) 9:e114746. 10.1371/jouRNAl.pone.0114746 25503963 PMC4264770

[244] Zhao H Wen J Dong X He R Gao C Zhang W. Identification of AQP3 and CD24 as biomarkers for carcinogenesis of gastric intestinal metaplasia. Oncotarget. (2017) 8:63382–91. 10.18632/oncotarget.18817 28968998 PMC5609930

[245] Fu Y Du P Zhao J Hu C'e Qin Y Huang G. Gastric cancer stem cells: mechanisms and therapeutic approaches. Yonsei Med J. (2018) 59:1150–8. 10.3349/ymj.2018.59.10.1150 30450848 PMC6240570

[246] Takaishi S Okumura T Tu S Wang SS Shibata W Vigneshwaran R. Identification of gastric cancer stem cells using the cell surface marker CD44. Stem Cells. (2009) 27:1006–20. 10.1002/stem.30 19415765 PMC2746367

[247] Zhang X Hua R Wang X Huang M Gan L Wu Z. Identification of stem-like cells and clinical significance of candidate stem cell markers in gastric cancer. Oncotarget. (2016) 7:9815–31. 10.18632/oncotarget.6890 26769843 PMC4891086

[248] Lau WM Teng E Chong HS Lopez KA Tay AY Salto-Tellez M. CD44v8-10 is a cancer-specific marker for gastric cancer stem cells. Cancer Res. (2014) 74:2630–41. 10.1158/0008-5472.CAN-13-2309 24618343

[249] Nguyen PH Giraud J Chambonnier L Dubus P Wittkop L Belleannée G. Characterization of biomarkers of tumorigenic and chemoresistant cancer stem cells in human gastric carcinoma. Clin Cancer Res. (2017) 23:1586–97. 10.1158/1078-0432.CCR-15-2157 27620279

[250] Nosrati A Naghshvar F Khanari S. Cancer stem cell markers CD44, CD133 in primary gastric adenocarcinoma. Int J Mol Cell Med. (2014) 3:279–86. 25635255 PMC4293616

[251] Shu X Liu H Pan Y Sun L Yu L Sun L. Distinct biological characterization of the CD44 and CD90 phenotypes of cancer stem cells in gastric cancer cell lines. Mol Cell Biochem. (2019) 459:35–47. 10.1007/s11010-019-03548-1 31073886

[252] Bekaii-Saab T El-Rayes B. Identifying and targeting cancer stem cells in the treatment of gastric cancer. Cancer. (2017) 123:1303–12. 10.1002/cncr.30538 28117883 PMC5412889

[253] Watanabe T Okumura T Hirano K Yamaguchi T Sekine S Nagata T. Circulating tumor cells expressing cancer stem cell marker CD44 as a diagnostic biomarker in patients with gastric cancer. Oncol Lett. (2017) 13:281–8. 10.3892/ol.2016.5432 28123556 PMC5244869

[254] Lu L Wu M Sun L Li W Fu W Zhang X. CLinicopathological and prognostic significance of cancer stem cell markers CD44 and CD133 in patients with gastric cancer: a comprehensive meta-analysis with 4729 patients involved. Medicine. (2016) 95:e5163. 10.1097/MD.0000000000005163 27759647 PMC5079331

[255] Jang E Kim E Son H-Y Lim E-K Lee H Choi Y. Nanovesicle-mediated systemic delivery of microRNA-34a for CD44 overexpressing gastric cancer stem cell therapy. Biomaterials. (2016) 105:12–24. 10.1016/j.biomaterials.2016.07.036 27497057

[256] Yao H-J Zhang Y-G Sun L Liu Y. The effect of hyaluronic acid functionalized carbon nanotubes loaded with salinomycin on gastric cancer stem cells. Biomaterials. (2014) 35:9208–23. 10.1016/j.biomaterials.2014.07.033 25115788

[257] Chen H Lin J Shan Y Zhengmao L. The promotion of nanopartiCLe delivery to two populations of gastric cancer stem cells by CD133 and CD44 antibodies. Biomed Pharmacothe. (2019) 115:108857. 10.1016/j.biopha.2019.108857 31048191

[258] Kodama H Murata S Ishida M Yamamoto H Yamaguchi T Kaida S. Prognostic impact of CD44-positive cancer stem-like cells at the invasive front of gastric cancer. Br J Cancer. (2017) 116:186–94. 10.1038/bjc.2016.401 27931044 PMC5243989

[259] Yoon C Park DJ Schmidt B Thomas NJ Lee H-J Kim TS. CD44 expression denotes a subpopulation of gastric cancer cells in which hedgehog signaling promotes chemotherapy resistance. Clin Cancer Res. (2014) 20:3974–88. 10.1158/1078-0432.CCR-14-0011 24947926 PMC4135312

[260] Senel F Kökenek Unal TD Karaman H Inanç M Aytekin A. Prognostic value of cancer stem cell markers CD44 and ALDH1/2 in gastric cancer cases. Asian Pac J Cancer Prev. (2017) 18:2527–31. 10.22034/APJCP.2017.18.9.2527 28952294 PMC5720661

[261] Howard R Al Diffalha S Pimiento J Mejia J Enderling H Giuliano A. CD133 expression as a helicobacter pylori-independent biomarker of gastric cancer progression. Anticancer Res. (2018) 38:4443–8. 10.21873/anticanres.12746 30061208 PMC7771274

[262] Xia P Song C-L Liu J-F Wang D Xu X-Y. Prognostic value of circulating CD133(+) cells in patients with gastric cancer. Cell Prolif . (2015) 48:311–7. 10.1111/cpr.12175 25727099 PMC6496317

[263] Yiming L Yunshan G Bo M Yu Z Tao W Gengfang L. CD133 overexpression correlates with CLinicopathological features of gastric cancer patients and its impact on survival: a systematic review and meta-analysis. Oncotarget. (2015) 6:42019–27. 10.18632/oncotarget.5714 26503471 PMC4747206

[264] Hashimoto K Aoyagi K Isobe T Kouhuji K Shirouzu K. Expression of CD133 in the cytoplasm is associated with cancer progression and poor prognosis in gastric cancer. Gastric Cancer. (2014) 17:97–106. 10.1007/s10120-013-0255-9 23558457 PMC3889295

[265] Chen X-L Chen X-Z Wang Y-G Du He Lu Z-H Liu K. Clinical significance of putative markers of cancer stem cells in gastric cancer: a retrospective cohort study. Oncotarget. (2016) 7:62049–69. 10.18632/oncotarget.11384 27557490 PMC5308710

[266] Fujita T Chiwaki F Takahashi R-U Aoyagi K Yanagihara K Nishimura T. Identification and characterization of CXCR4-Positive gastric cancer stem cells. PLoS ONE. (2015) 10:e0130808. 10.1371/jouRNAl.pone.0130808 26110809 PMC4481351

[267] Xue L-J Mao X-B Ren L-L Chu X-Y. Inhibition of CXCL12/CXCR4 axis as a potential targeted therapy of advanced gastric carcinoma. Cancer Med. (2017) 6:1424–36. 10.1002/cam4.1085 28544785 PMC5463074

[268] Yang W Lai Z Li Y Mu J Yang M Xie J. Immune signature profiling identified prognostic factors for gastric cancer. Chin J Cancer Res. (2019) 31:463–70. 10.21147/j.issn.1000-9604.2019.03.08 31354215 PMC6613504

[269] Yu C Zhang Y. Characterization of the prognostic values of CXCR family in gastric cancer. Cytokine. (2019) 123:154785. 10.1016/j.cyto.2019.154785 31344595

[270] Jiang Q Sun Y Liu X. CXCR4 as a prognostic biomarker in gastrointestinal cancer: a meta-analysis. Biomarkers. (2019) 24:510–6. 10.1080/1354750X.2019.1637941 31244335

[271] Han M Lv S Zhang Y Yi R Huang B Fu H. The prognosis and CLinicopathology of CXCR4 in gastric cancer patients: a meta-analysis. Tumour Biol. (2014) 35:4589–97. 10.1007/s13277-013-1603-4 24464926

[272] Dai M Yuan F Fu C Shen G Hu S Shen G. Relationship between epithelial cell adhesion molecule (EpCAM) overexpression and gastric cancer patients: a systematic review and meta-analysis. PLoS ONE. (2017) 12:e0175357. 10.1371/jouRNAl.pone.0175357 28403178 PMC5389808

[273] Knödler M Körfer J Kunzmann V Trojan J Daum S Schenk M. Randomised phase iI trial to investigate catumaxomab (anti-EpCAM × anti-CD3) for treatment of peritoneal carcinomatosis in patients with gastric cancer. Br J Cancer. (2018) 119:296–302. 10.1038/s41416-018-0150-6 29988111 PMC6070920

[274] Nakajima T Uehara T Maruyama Y Iwaya M Kobayashi Y Ota H. Distribution of Lgr5-positive cancer cells in intramucosal gastric signet-ring cell carcinoma. Pathol Int. (2016) 66:518–23. 10.1111/pin.12451 27593551

[275] Xi HQ Cai AZ Wu XS Cui JX Shen WS Bian SB. Leucine-rich repeat-containing g-protein-coupled receptor 5 is associated with invasion, metastasis, and could be a potential therapeutic target in human gastric cancer. Br J Cancer. (2014) 110:2011–20. 10.1038/bjc.2014.112 24594994 PMC3992491

[276] Gong X Azhdarinia A Ghosh SC Xiong W An Z Liu Q. LGR5-Targeted antibody-drug conjugate eradicates gastrointestinal tumors and prevents recurrence. Mol Cancer Ther. (2016) 15:1580–90. 10.1158/1535-7163.MCT-16-0114 27207778

[277] Xi H-Q Cui J-X Shen W-S Wu X-S Bian S-B Li J-Y. Increased expression of Lgr5 is associated with chemotherapy resistance in human gastric cancer. Oncol Rep. (2014) 32:181–8. 10.3892/or.2014.3207 24859092

[278] Liu X-S Lin X-K Mei Y Ahmad S Yan C-X Jin H-L. Regulatory T cells promote overexpression of Lgr5 on gastric cancer cells via TGF-beta1 and confer poor prognosis in gastric cancer. Front Immunol. (2019) 10:1741. 10.3389/fimmu.2019.01741 31417548 PMC6682668

[279] Huang T Qiu X Xiao J Wang Q Wang Y Zhang Y. The prognostic role of leucine-rich repeat-containing G-protein-coupled receptor 5 in gastric cancer: a systematic review with meta-analysis. Clin Res Hepatol Gastroenterol. (2016) 40:246–53. 10.1016/j.CLinre.2015.07.009 26387842

[280] Jo JH Park SB Park S Lee HS Kim C Jung DE. Novel gastric cancer stem cell-Related marker lINGO2 is associated with cancer cell phenotype and patient outcome. Int J Mol Sci. (2019) 20:555. 10.3390/ijms20030555 30696080 PMC6387145

[281] Nishikawa S Konno M Hamabe A Hasegawa S Kano Y Ohta K. Aldehyde dehydrogenase high gastric cancer stem cells are resistant to chemotherapy. Int J Oncol. (2013) 42:1437–42. 10.3892/ijo.2013.1837 23440340

[282] Di Wu Mou Y-P Chen K Cai J-Q Zhou Y-C Pan Y. Aldehyde dehydrogenase 3A1 is robustly upregulated in gastric cancer stem-like cells and associated with tumorigenesis. Int J Oncol. (2016) 49:611–22. 10.3892/ijo.2016.3551 27279633

[283] Li H Piao L Xu D Xuan Y. LETM1 is a potential biomarker that predicts poor prognosis in gastric adenocarcinoma. Exp Mol Pathol. (2019) 112:104333. 10.1016/j.yexmp.2019.104333 31705880

[284] Yang Z Li J Shi Y Li L Guo X. Increased musashi 2 expression indicates a poor prognosis and promotes malignant phenotypes in gastric cancer. Oncol Lett. (2019) 17:2599–606. 10.3892/ol.2019.9889 30854035 PMC6365935

[285] Wang B Chen Q Cao Y Ma X Yin C Jia Y. LGR5 is a gastric cancer stem cell marker associated with stemness and the EMT signature genes NANOG, NANOGP8, PRRX1, TWIST1, and BMI1. PLoS ONE. (2016) 11:e0168904. 10.1371/jouRNAl.pone.0168904 28033430 PMC5199039

[286] Santaliz-Ruiz LE Xie X Old M Teknos TN Pan Q. Emerging role of nanog in tumorigenesis and cancer stem cells. Int J Cancer. (2014) 135:2741–8. 10.1002/ijc.28690 24375318 PMC4065638

[287] Lin T Ding Y-Q Li J-M. Overexpression of nanog protein is associated with poor prognosis in gastric adenocarcinoma. Med Oncol. (2012) 29:878–85. 10.1007/s12032-011-9860-9 21336986

[288] Basati G Mohammadpour H Emami Razavi A. Association of high expression levels of SOX2, NANOG, and OCT4 in gastric cancer tumor tissues with progression and poor prognosis. J Gastrointest Cancer. (2019) 51:41–7. 10.1007/s12029-018-00200-x 30628031

[289] Chen Z Xu W-R Qian H Zhu W Bu X-F Wang S. Oct4, a novel marker for human gastric cancer. J Surg Oncol. (2009) 99:414–9. 10.1002/jso.21270 19347886

[290] Carrasco-Garcia E Álvarez-Satta M García-Puga M Ribeiro ML Arevalo S Arauzo-Bravo M. Therapeutic relevance of SOX9 stem cell factor in gastric cancer. Expert Opin Ther Targets. (2019) 23:143–52. 10.1080/14728222.2019.1559826 30572738

[291] Luo J Yan R He X He J. SOX2 inhibits cell proliferation and metastasis, promotes apoptotic by downregulating cCND1 and pARP in gastric cancer. Am J Transl Res. (2018) 10:639–47. 29511458 PMC5835829

[292] Hütz K Mejías-Luque R Farsakova K Ogris M Krebs S Anton M. The stem cell factor SOX2 regulates the tumorigenic potential in human gastric cancer cells. Carcinogenesis. (2014) 35:942–50. 10.1093/carcin/bgt410 24325912

[293] Chen Y Huang Y Zhu L Chen M Huang Y Zhang J. SOX2 inhibits metastasis in gastric cancer. J Cancer Res Clin Oncol. (2016) 142:1221–30. 10.1007/s00432-016-2125-4 26960758 PMC11819337

[294] Tsai S-C Lin C-C Shih T-C Tseng R-J Yu M-C Lin Y-J. The miR-200b-ZEB1 circuit regulates diverse stemness of human hepatocellular carcinoma. Mol Carcinog. (2017) 56:2035–47. 10.1002/mc.22657 28383782

[295] Wang R Sun Q Wang P Liu M Xiong S Luo J. Notch and wnt/β-catenin signaling pathway play important roles in activating liver cancer stem cells. Oncotarget. (2016) 7:5754–68. 10.18632/oncotarget.6805 26735577 PMC4868719

[296] Wang R Li Y Tsung A Huang H Du Q Yang M. iNOS promotes CD24+CD133+ liver cancer stem cell phenotype through a TACE/ADAM17-dependent notch signaling pathway. Proc Natl Acad Sci USA. (2018) 115:E10127–36. 10.1073/pnas.1722100115 30297396 PMC6205478

[297] Sukowati CHC. Heterogeneity of hepatic cancer stem cells. Adv Exp Med Biol. (2019) 1139:59–81. 10.1007/978-3-030-14366-4_4 31134495

[298] Xiang Y Yang T Pang B-Y Zhu Y Liu YN. The progress and prospects of putative biomarkers for liver cancer stem cells in hepatocellular carcinoma. Stem Cells Int. (2016) 2016:7614971. 10.1155/2016/7614971 27610139 PMC5005617

[299] Asai R Tsuchiya H Amisaki M Makimoto K Takenaga A Sakabe T. CD44 standard isoform is involved in maintenance of cancer stem cells of a hepatocellular carcinoma cell line. Cancer Med. (2019) 8:773–82. 10.1002/cam4.1968 30636370 PMC6382709

[300] Liu R Shen Y Nan K Mi B Wu T Guo J. Association between expression of cancer stem cell markers and poor differentiation of hepatocellular carcinoma: a meta-analysis (PRISMA). Medicine. (2015) 94:e1306. 10.1097/MD.0000000000001306 26252310 PMC4616593

[301] Choi GH Kim GI Yoo JE Na DC Han DH Roh YH. Increased expression of circulating cancer stem cell markers during the perioperative period predicts early recurrence after curative resection of hepatocellular carcinoma. Ann Surg Oncol. (2015) 22(Suppl. 3):S1444–52. 10.1245/s10434-015-4480-9 25791790

[302] Luo Y Tan Y. Prognostic value of CD44 expression in patients with hepatocellular carcinoma: meta-analysis. Cancer Cell Int. (2016) 16:47. 10.1186/s12935-016-0325-2 27330410 PMC4912706

[303] Morine Y Imura S Ikemoto T Iwahashi S Saito Yu Shimada M. CD44 expression is a prognostic factor in patients with intrahepatic cholangiocarcinoma after surgical resection. Anticancer Res. (2017) 37:5701–5. 10.21873/anticanres.12007 28982889

[304] Kim BH Park J-W Kim JS Lee S-K Hong EK. Stem cell markers predict the response to sorafenib in patients with hepatocellular carcinoma. Gut Liver. (2019) 13:342–8. 10.5009/gnl18345 30600675 PMC6529171

[305] Luo J Wang P Wang R Wang J Liu M Xiong S The notch pathway promotes the cancer stem cell characteristics of CD90+ cells in hepatocellular carcinoma. Oncotarget. (2016) 7:9525–37. 10.18632/oncotarget.6672 26848615 PMC4891057

[306] Yoshida M Yamashita T Okada H Oishi N Nio K Hayashi T. Sorafenib suppresses extrahepatic metastasis *de novo* in hepatocellular carcinoma through inhibition of mesenchymal cancer stem cells characterized by the expression of CD90. Sci. Rep. (2017) 7:11292. 10.1038/s41598-017-11848-z 28900199 PMC5596021

[307] Zhang K Che S Su Z Zheng S Zhang H Yang S. CD90 promotes cell migration, viability and sphere-forming ability of hepatocellular carcinoma cells. Int J Mol Med. (2018) 41:946–54. 10.3892/ijmm.2017.3314 29251325 PMC5752240

[308] Zhu L Zhang W Wang J Liu R. Evidence of CD90+CXCR4+ cells as circulating tumor stem cells in hepatocellular carcinoma. Tumour Biol. (2015) 36:5353–60. 10.1007/s13277-015-3196-6 25672610

[309] Zhao RC Zhou J Chen KF Gong J Liu J He JY. The prognostic value of combination of CD90 and OCT4 for hepatocellular carcinoma after curative resection. Neoplasma. (2016) 63:288–98. 10.4149/neo_2016_036 26674131

[310] Suetsugu A Nagaki M Aoki H Motohashi T Kunisada T Moriwaki H. Characterization of CD133+ hepatocellular carcinoma cells as cancer stem/progenitor cells. Biochem Biophys Res Commun. (2006) 351:820–4. 10.1016/j.bbrc.2006.10.128 17097610

[311] Li M-M Tang Y-Q Gong Y-F Cheng W Li H-L Kong F-E. Development of an oncogenic dedifferentiation SOX signature with prognostic significance in hepatocellular carcinoma. BMC Cancer. (2019) 19:851. 10.1186/s12885-019-6041-2 31462277 PMC6714407

[312] Liu K Hao M Ouyang Y Zheng J Chen D. CD133+ cancer stem cells promoted by VEGF accelerate the recurrence of hepatocellular carcinoma. Sci. Rep. (2017) 7:41499. 10.1038/srep41499 28134312 PMC5278354

[313] Cheung PF Cheung TT Yip CW Ng LW Fung SW Lo CM. Hepatic cancer stem cell marker granulin-epithelin precursor and β-catenin expression associate with recurrence in hepatocellular carcinoma. Oncotarget. (2016) 7:21644–57. 10.18632/oncotarget.7803 26942873 PMC5008312

[314] Jun SY Jeon S-J Yoon J-Y Lee J-J Yoon HR Choi M-H. The positive correlation of TIPRL with lC3 and CD133 contributes to cancer aggressiveness: potential biomarkers for early liver cancer. Sci. Rep. (2019) 9:16802. 10.1038/s41598-019-53191-5 31727942 PMC6856114

[315] Vilchez V Turcios L Zaytseva Y Stewart R Lee EY Maynard E. Cancer stem cell marker expression alone and in combination with microvascular invasion predicts poor prognosis in patients undergoing transplantation for hepatocellular carcinoma. Am J Surg. (2016) 212:238–45. 10.1016/j.amjsurg.2015.12.019 27033253

[316] Chen D Li Z Cheng Q Wang Y Qian L Gao J. Genetic alterations and expression of PTEN and its relationship with cancer stem cell markers to investigate pathogenesis and to evaluate prognosis in hepatocellular carcinoma. J Clin Pathol. (2019) 72:588–96. 10.1136/jCLinpath-2019-205769 31126975

[317] Ding Q Xia Y Ding S Lu P Sun L Liu M. An alternatively spliced variant of CXCR3 mediates the metastasis of CD133+ liver cancer cells induced by CXCL9. Oncotarget. (2016) 7:14405–14. 10.18632/oncotarget.7360 26883105 PMC4924724

[318] Su R Nan H Guo H Ruan Z Jiang L Song Y. Associations of components of PTEN/AKT/mTOR pathway with cancer stem cell markers and prognostic value of these biomarkers in hepatocellular carcinoma. Hepatol Res. (2016) 46:1380–91. 10.1111/hepr.12687 26932478

[319] Yu G-F Lin X Luo R-C Fang W-Y. Nuclear CD133 expression predicts poor prognosis for hepatocellular carcinoma. Int J Clin Exp Pathol. (2018) 11:2092–9. 31938317 PMC6958201

[320] Chen Y-L Lin P-Y Ming Y-Z Huang W-C Chen R-F Chen P-M. The effects of the location of cancer stem cell marker CD133 on the prognosis of hepatocellular carcinoma patients. BMC Cancer. (2017) 17:474. 10.1186/s12885-017-3460-9 28687090 PMC5501948

[321] Seino S Tsuchiya A Watanabe Y Kawata Y Kojima Y Ikarashi S. Clinical outcome of hepatocellular carcinoma can be predicted by the expression of hepatic progenitor cell markers and serum tumour markers. Oncotarget. (2018) 9:21844–60. 10.18632/oncotarget.25074 29774107 PMC5955154

[322] Felden J von Schulze K Krech T Ewald F Nashan B Pantel K. Circulating tumor cells as liquid biomarker for high HCC recurrence risk after curative liver resection. Oncotarget. (2017) 8:89978–87. 10.18632/oncotarget.21208 29163804 PMC5685725

[323] Dai X-M Huang T Yang S-L Zheng X-M Chen GG Zhang T Peritumoral EpCAM is an independent prognostic marker after curative resection of HBV-Related hepatocellular carcinoma. Dis Markers. (2017) 2017:8495326 10.1155/2017/3765279 28572700 PMC5442434

[324] Ko C-J Li C-J Wu M-Y Chu P-Y. Overexpression of epithelial cell adhesion molecule as a predictor of poor outcome in patients with hepatocellular carcinoma. Exp Ther Med. (2018) 16:4810–6. 10.3892/etm.2018.6794 30542436 PMC6257457

[325] Shen J Wang W-S Zhu X-L Ni C-F. High epithelial cell adhesion molecule-Positive circulating tumor cell count predicts poor survival of patients with unresectable hepatocellular carcinoma treated with transcatheter arterial chemoembolization. J Vasc Interv Radiol. (2018) 29:1678–84. 10.1016/j.jvir.2018.07.030 30392801

[326] Noh C-K Wang HJ Kim CM Kim J Yoon SY Lee GH. EpCAM as a predictive marker of tumor recurrence and survival in patients who underwent surgical resection for hepatocellular carcinoma. Anticancer Res. (2018) 38:4101–9. 10.21873/anticanres.12700 29970536

[327] Zhou L Zhu Y. The EpCAM overexpression is associated with Clinicopathological significance and prognosis in hepatocellular carcinoma patients: a systematic review and meta-analysis. Int J Surg. (2018) 56:274–80. 10.1016/j.ijsu.2018.06.025 29936198

[328] Zhu M Li W Lu Y Dong X Chen Y Lin B. Alpha fetoprotein antagonizes apoptosis induced by paclitaxel in hepatoma cells *in vitro*. Sci. Rep. (2016) 6:26472. 10.1038/srep26472 27255186 PMC4891737

[329] Jin J Niu X Zou L Li L Li S Han J. AFP mRNA level in enriched circulating tumor cells from hepatocellular carcinoma patient blood samples is a pivotal predictive marker for metastasis. Cancer Lett. (2016) 378:33–7. 10.1016/j.canlet.2016.04.033 27160647

[330] Lorente L. New prognostic biomarkers of mortality in patients undergoing liver transplantation for hepatocellular carcinoma. World J Gastroenterol. (2018) 24:4230–42. 10.3748/wjg.v24.i37.4230 30310256 PMC6175764

[331] Chang T-S Wu Y-C Chi C-C Su W-C Chang P-J Lee K-F. Activation of IL6/IGFIR confers poor prognosis of HBV-related hepatocellular carcinoma through induction of OCT4/NANOG expression. Clin Cancer Res. (2015) 21:201–10. 10.1158/1078-0432.CCR-13-3274 25564572

[332] Was H Czarnecka J Kominek A Barszcz K BeRNAs T Piwocka K . Some chemotherapeutics-treated colon cancer cells display a specific phenotype being a combination of stem-like and senescent cell features. Cancer Biol Ther. (2018) 19:63–75. 10.1080/15384047.2017.1385675 29053388 PMC5790359

[333] Manhas J Bhattacharya A Agrawal SK Gupta B Das P Deo SV. Characterization of cancer stem cells from different grades of human colorectal cancer. Tumour Biol. (2016) 37:14069–81. 10.1007/s13277-016-5232-6 27507615

[334] Zhou J-Y Chen M Ma L Wang X Chen Y-G Liu S-L. Role of CD44(high)/CD133(high) HCT-116 cells in the tumorigenesis of colon cancer. Oncotarget. (2016) 7:7657–66. 10.18632/oncotarget.7084 26840024 PMC4884945

[335] Leng Z Xia Q Chen J Li Y Xu J Zhao E. Lgr5+CD44+EpCAM+ strictly defines cancer stem cells in human colorectal cancer. Cell Physiol Biochem. (2018) 46:860–72. 10.1159/000488743 29627827

[336] Zhou Y Xia L Wang H Oyang L Su M Liu Q Cancer stem cells in progression of colorectal cancer. Oncotarget. (2018) 9:33403–15. 10.18632/oncotarget.23607 30279970 PMC6161799

[337] Lee SY Kim KA Kim CH Kim YJ Lee J-H Kim HR. CD44-shRNA recombinant adenovirus inhibits cell proliferation, invasion, and migration, and promotes apoptosis in HCT116 colon cancer cells. Int J Oncol. (2017) 50:329–36. 10.3892/ijo.2016.3801 27959393

[338] Tsunekuni K Konno M Haraguchi N Koseki J Asai A Matsuoka K. CD44/CD133-positive colorectal cancer stem cells are sensitive to trifluridine exposure. Sci Rep. (2019) 9:14861. 10.1038/s41598-019-50968-6 31619711 PMC6795793

[339] Ozawa M Ichikawa Y Zheng Y-W Oshima T Miyata H Nakazawa K. Prognostic significance of CD44 variant 2 upregulation in colorectal cancer. Br J Cancer. (2014) 111:365–74. 10.1038/bjc.2014.253 24921913 PMC4102936

[340] Lim SH Jang J Park JO Kim K-M Kim ST Park YS. CD133-positive tumor cell content is a predictor of early recurrence in colorectal cancer. J Gastrointest Oncol. (2014) 5:447–56. 10.3978/j.issn.2078-6891.2014.071 25436124 PMC4226826

[341] Ning S-T Lee S-Y Wei M-F Peng C-L Lin SY-F Tsai M-H. Targeting colorectal cancer stem-like cells with anti-CD133 antibody-Conjugated SN-38 nanopartiCLes. ACS Appl Mater Interfaces. (2016) 8:17793–804. 10.1021/acsami.6b04403 27348241

[342] Zhao L Yang Y Zhou P Ma H Zhao X He X. Targeting CD133high colorectal cancer cells *in vitro* and *in vivo* with an asymmetric bispecific antibody. J Immunother. (2015) 38:217–28. 10.1097/CJI.0000000000000086 26049545

[343] Schmohl JU Gleason MK Dougherty PR Miller JS Vallera DA. Heterodimeric bispecific single chain variable fragments (ScFv) killer engagers (BiKEs) enhance NK-cell activity against CD133+ colorectal cancer cells. Target Oncol. (2016) 11:353–61. 10.1007/s11523-015-0391-8 26566946 PMC4873478

[344] Zhao Y Peng J Zhang E Jiang N Li J Zhang Q. CD133 expression may be useful as a prognostic indicator in colorectal cancer, a tool for opTIMizing therapy and supportive evidence for the cancer stem cell hypothesis: a meta-analysis. Oncotarget. (2016) 7:10023–36. 10.18632/oncotarget.7054 26840260 PMC4891101

[345] AlShamaileh H Wang T Xiang D Yin W Tran PH-L Barrero RA. Aptamer-mediated survivin RNAi enables 5-fluorouracil to eliminate colorectal cancer stem cells. Sci Rep. (2017) 7:5898. 10.1038/s41598-017-05859-z 28724889 PMC5517644

[346] Xiang D Shigdar S Bean AG Bruce M Yang W Mathesh M. Transforming doxorubicin into a cancer stem cell killer via EpCAM aptamer-mediated delivery. Theranostics. (2017) 7:4071–86. 10.7150/thno.20168 29158811 PMC5694998

[347] Boesch M Spizzo G Seeber A. Concise review: aggressive colorectal cancer: role of epithelial cell adhesion molecule in cancer stem cells and epithelial-to-Mesenchymal transition. Stem Cells Transl Med. (2018) 7:495–501. 10.1002/sctm.17-0289 29667344 PMC5980125

[348] Sousa e Melo F de Kurtova AV Harnoss JM Kljavin N Hoeck JD Hung J. A distinct role for Lgr5+ stem cells in primary and metastatic colon cancer. Nature. (2017) 543:676–80. 10.1038/nature21713 28358093

[349] Shimokawa M Ohta Y Nishikori S Matano M Takano A Fujii M. Visualization and targeting of Lgr5+ human colon cancer stem cells. Nature. (2017) 545:187–92. 10.1038/nature22081 28355176

[350] Cortina C Turon G Stork D HeRNAndo-Momblona X Sevillano M Aguilera M. A genome editing approach to study cancer stem cells in human tumors. Embo Mol Med. (2017) 9:869–79. 10.15252/emmm.201707550 28468934 PMC5494503

[351] Baker A-M Graham TA Elia G Wright NA Rodriguez-Justo M. Characterization of Lgr5 stem cells in colorectal adenomas and carcinomas. Sci Rep. (2015) 5:8654. 10.1038/srep08654 25728748 PMC4345329

[352] Junttila MR Mao W Wang X Wang B-E Pham T Flygare J. Targeting Lgr5+ cells with an antibody-drug conjugate for the treatment of colon cancer. Sci Transl Med. (2015) 7:314ra186. 10.1126/scitranslmed.aac7433 26582901

[353] He S Zhou H Zhu X Hu S Fei M Wan D. Expression of Lgr5, a marker of intestinal stem cells, in colorectal cancer and its CLinicopathological significance. Biomed Pharmacother. (2014) 68:507–13. 10.1016/j.biopha.2014.03.016 24751002

[354] Jiang Y Li W He X Zhang H Jiang F Chen Z. Lgr5 expression is a valuable prognostic factor for colorectal cancer: evidence from a meta-analysis. BMC Cancer. (2015) 15:948. 10.1186/s12885-015-1985-3 26674601 PMC4682230

[355] Vishnubalaji R Manikandan M Fahad M Hamam R Alfayez M Kassem M. Molecular profiling of ALDH1+ colorectal cancer stem cells reveals preferential activation of mAPK, FAK, and oxidative stress pro-survival signalling pathways. Oncotarget. (2018) 9:13551–64. 10.18632/oncotarget.24420 29568377 PMC5862598

[356] Kozovska Z Patsalias A Bajzik V Durinikova E Demkova L Jargasova S. ALDH1A inhibition sensitizes colon cancer cells to chemotherapy. BMC Cancer. (2018) 18:656. 10.1186/s12885-018-4572-6 29902974 PMC6003038

[357] Kahlert C Gaitzsch E Steinert G Mogler C Herpel E Hoffmeister M. Expression analysis of aldehyde dehydrogenase 1A1 (ALDH1A1) in colon and rectal cancer in association with prognosis and response to chemotherapy. Ann Surg Oncol. (2012) 19:4193–201. 10.1245/s10434-012-2518-9 22878609

[358] Piao L Feng Y Yang Z Qi W Li H Han H. LETM1 is a potential cancer stem-like cell marker and predicts poor prognosis in colorectal adenocarcinoma. Pathol Res Pract. (2019) 215:152437. 10.1016/j.prp.2019.152437 31101574

[359] Yang L Ding C Tang W Yang T Liu M Wu H. INPP4B exerts a dual function in the stemness of colorectal cancer stem-like cells through regulating sox2 and nanog expression. Carcinogenesis. (2019) 41:78–90. 10.1093/carcin/bgz110 31179504

[360] Yao C Su L Shan J Zhu C Liu L Liu C. IGF/STAT3/NANOG/Slug signaling axis simultaneously controls epithelial-Mesenchymal transition and stemness maintenance in colorectal cancer. Stem Cells. (2016) 34:820–31. 10.1002/stem.2320 26840943

[361] Wang H Liu B Wang J Li J Gong Y Li S. Reduction of NANOG mediates the inhibitory effect of aspirin on tumor growth and stemness in colorectal cancer. Cell Physiol Biochem. (2017) 44:1051–63. 10.1159/000485405 29179207

[362] Xu F Dai C Zhang R Zhao Y Peng S Jia C. Nanog: a potential biomarker for liver metastasis of colorectal cancer. Dig Dis Sci. (2012) 57:2340–6. 10.1007/s10620-012-2182-8 22562535

[363] Fujino S Miyoshi N. Oct4 gene expression in primary colorectal cancer promotes liver metastasis. Stem Cells Int. (2019) 2019:7896524. 10.1155/2019/7896524 31191684 PMC6525814

[364] Lee JH Yun CW Han Y-S Kim S Jeong D Kwon HY. Melatonin and 5-fluorouracil co-suppress colon cancer stem cells by regulating cellular prion protein-Oct4 axis. J Pineal Res. (2018) 65:e12519. 10.1111/jpi.12519 30091203

[365] Miyoshi N Fujino S Ohue M Yasui M Takahashi Y Sugimura K. The POU5F1 gene expression in colorectal cancer: a novel prognostic marker. Surg Today. (2018) 48:709–15. 10.1007/s00595-018-1644-9 29488015

[366] Ardalan Khales S Abbaszadegan MR Abdollahi A Raeisossadati R Tousi MF Forghanifard MM. SALL4 as a new biomarker for early colorectal cancers. J Cancer Res Clin Oncol. (2015) 141:229–35. 10.1007/s00432-014-1808-y 25156818 PMC11824103

[367] Lundberg IV Edin S Eklöf V Öberg Å Palmqvist R Wikberg ML. SOX2 expression is associated with a cancer stem cell state and down-regulation of CDX2 in colorectal cancer. BMC Cancer. (2016) 16:471. 10.1186/s12885-016-2509-5 27411517 PMC4944515

[368] Takeda K Mizushima T Yokoyama Y Hirose H Wu X Qian Y. Sox2 is associated with cancer stem-like properties in colorectal cancer. Sci Rep. (2018) 8:17639. 10.1038/s41598-018-36251-0 30518951 PMC6281572

[369] Miller TJ McCoy MJ Hemmings C Bulsara MK Iacopetta B Platell CF. The prognostic value of cancer stem-like cell markers SOX2 and CD133 in stage iII colon cancer is modified by expression of the immune-related markers FoxP3, PD-L1 and CD3. Pathology. (2017) 49:721–30. 10.1016/j.pathol.2017.08.007 29102042

[370] Haubner S PeRNA F Köhnke T Schmidt C Berman S Augsberger C. Coexpression profile of leukemic stem cell markers for combinatorial targeted therapy in AML. Leukemia. (2019) 33:64–74. 10.1038/s41375-018-0180-3 29946192 PMC6326956

[371] Zhang CC Yan Z Pascual B Jackson-Fisher A Huang DS Zong Q. Gemtuzumab ozogamicin (GO) inclusion to induction chemotherapy eliminates leukemic initiating cells and significantly improves survival in mouse models of acute myeloid leukemia. Neoplasia. (2018) 20:1–11. 10.1016/j.neo.2017.10.008 29172076 PMC5702869

[372] Tang X Yang L Li Z Nalin AP Dai H Xu T First-in-man clinical trial of CAR NK-92 cells: safety test of CD33-CAR NK-92 cells in patients with relapsed and refractory acute myeloid leukemia. Am J Cancer Res. (2018) 8:1083–9. 30034945 PMC6048396

[373] Braciak TA Roskopf CC Wildenhain S Fenn NC Schiller CB Schubert IA. Dual-targeting triplebody 33-16-123 (SPM-2) mediates effective redirected lysis of primary blasts from patients with a broad range of AML subtypes in combination with natural killer cells. Oncoimmunology. (2018) 7:e1472195. 10.1080/2162402X.2018.1472195 30228941 PMC6140553

[374] Petrov JC Wada M Pinz KG Yan LE Chen KH Shuai X. Compound CAR T-cells as a double-pronged approach for treating acute myeloid leukemia. Leukemia. (2018) 32:1317–26. 10.1038/s41375-018-0075-3 29515236 PMC5990523

[375] Cartellieri M Feldmann A Koristka S Arndt C Loff S Ehninger A. Switching CAR T cells on and off: a novel modular platform for retargeting of t cells to AML blasts. Blood Cancer J. (2016) 6:e458. 10.1038/bcj.2016.61 27518241 PMC5022178

[376] Pizzitola I Anjos-Afonso F Rouault-Pierre K Lassailly F Tettamanti S Spinelli O. Chimeric antigen receptors against CD33/CD123 antigens efficiently target primary acute myeloid leukemia cells *in vivo*. Leukemia. (2014) 28:1596–605. 10.1038/leu.2014.62 24504024

[377] Vallera DA Felices M McElmurry R McCullar V Zhou X Schmohl JU. IL15 trispecific killer engagers (Trike) make natural killer cells specific to CD33+ targets while also inducing persistence, *in vivo* expansion, and enhanced function. Clin Cancer Res. (2016) 22:3440–50. 10.1158/1078-0432.CCR-15-2710 26847056 PMC4947440

[378] Hoseini SS Guo H Wu Z Hatano MN Cheung N-KV. A potent tetravalent t-cell-engaging bispecific antibody against CD33 in acute myeloid leukemia. Blood Adv. (2018) 2:1250–8. 10.1182/bloodadvances.2017014373 29858209 PMC5998923

[379] Kovtun Y Noordhuis P Whiteman KR Watkins K Jones GE Harvey L. IMGN779, a novel CD33-Targeting antibody–drug conjugate with DNA-Alkylating activity, exhibits potent antitumor activity in models of AML. Mol Cancer Ther. (2018) 17:1271–9. 10.1158/1535-7163.MCT-17-1077 29588393

[380] Vasu S He S Cheney C Gopalakrishnan B Mani R Lozanski G. Decitabine enhances anti-CD33 monoclonal antibody BI 836858-mediated natural killer ADCC against AML blasts. Blood. (2016) 127:2879–89. 10.1182/blood-2015-11-680546 27013443 PMC4900955

[381] Kloess S Ede Valverde da Silva A Oberschmidt O Gardlowski T Matthies N Vyas M. Triplebody mediates increased anti-Leukemic reactivity of IL-2 activated donor natural killer (NK) cells and impairs viability of their CD33-expressing NK subset. Front Immunol. (2017) 8:1100. 10.3389/fimmu.2017.01100 28943878 PMC5596090

[382] Schneider D Xiong Y Hu P Wu D Chen W Ying T. A unique human immunoglobulin heavy chain variable domain-only CD33 CAR for the treatment of acute myeloid leukemia. Front. Oncol. (2018) 8:539. 10.3389/fonc.2018.00539 30524966 PMC6262782

[383] Kenderian SS Ruella M Shestova O Klichinsky M Aikawa V Morrissette JJ. CD33-specific chimeric antigen receptor T cells exhibit potent preClinical activity against human acute myeloid leukemia. Leukemia. (2015) 29:1637–47. 10.1038/leu.2015.52 25721896 PMC4644600

[384] Hills RK Castaigne S Appelbaum FR Delaunay J Petersdorf S Othus M. Addition of gemtuzumab ozogamicin to induction chemotherapy in adult patients with acute myeloid leukaemia: a meta-analysis of individual patient data from randomised controlled trials. Lancet Oncol. (2014) 15:986–96. 10.1016/S1470-2045(14)70281-5 25008258 PMC4137593

[385] Gamis AS Alonzo TA Meshinchi S Sung L Gerbing RB Raimondi SC. Gemtuzumab ozogamicin in children and adolescents with *de novo* acute myeloid leukemia improves event-free survival by reducing relapse risk: results from the randomized phase iII children's oncology group trial AAML0531. JCO. (2014) 32:3021–32. 10.1200/JCO.2014.55.3628 25092781 PMC4162498

[386] Stein EM Walter RB Erba HP Fathi AT Advani AS Lancet JE. A phase 1 trial of vadastuximab talirine as monotherapy in patients with CD33-positive acute myeloid leukemia. Blood. (2018) 131:387–96. 10.1182/blood-2017-06-789800 29196412 PMC5813721

[387] Wang Q-S Wang Y Lv H-Y Han Q-W Fan H Guo B. Treatment of CD33-directed chimeric antigen receptor-modified T cells in one patient with relapsed and refractory acute myeloid leukemia. Mol Ther. (2015) 23:184–91. 10.1038/mt.2014.164 25174587 PMC4426796

[388] Zahler S Bhatia M Ricci A Roy S Morris E Harrison L. A phase I Study of reduced-Intensity conditioning and allogeneic stem cell transplantation followed by dose escalation of targeted consolidation immunotherapy with gemtuzumab ozogamicin in children and adolescents with CD33+ acute myeloid leukemia. Biol Blood Marrow Transpl. (2016) 22:698–704. 10.1016/j.bbmt.2016.01.019 26785332

[389] Niktoreh N Lerius B Zimmermann M Gruhn B Escherich G Bourquin J-P. Gemtuzumab ozogamicin in children with relapsed or refractory acute myeloid leukemia: a report by Berlin-Frankfurt-Münster study group. Haematologica. (2019) 104:120–7. 10.3324/haematol.2018.191841 30093401 PMC6312035

[390] Tarlock K Alonzo TA Gerbing RB Raimondi SC Hirsch BA Sung L. Gemtuzumab ozogamicin reduces relapse risk in FLT3/ITD acute myeloid leukemia: a report from the children's oncology group. Clin Cancer Res. (2016) 22:1951–7. 10.1158/1078-0432.CCR-15-1349 26644412 PMC4834220

[391] Amadori S Suciu S Selleslag D Aversa F Gaidano G Musso M. Gemtuzumab ozogamicin versus best supportive care in older patients with newly diagnosed acute myeloid leukemia unsuitable for intensive chemotherapy: results of the randomized phase III EORTC-GIMEMA AML-19 trial. J Clin Oncol. (2016) 34:972–9. 10.1200/JCO.2015.64.0060 26811524

[392] Portwood S Puchalski RA Walker RM Wang ES Combining IMGN779, a novel anti-CD33 antibody-drug conjugate (ADC), with the pARP inhibitor, olaparib, results in enhanced anti-Tumor activity in preclinical acute myeloid leukemia (AML) models. Blood. (2016) 128:1645 10.1182/blood.V128.22.1645.1645

[393] Pollard JA Loken M Gerbing RB Raimondi SC Hirsch BA Aplenc R. CD33 expression and its association with gemtuzumab ozogamicin response: results from the randomized phase III children's oncology group trial AAML0531. J Clin Oncol. (2016) 34:747–55. 10.1200/JCO.2015.62.6846 26786921 PMC4872025

[394] Yabushita T Satake H Maruoka H Morita M Katoh D Shimomura Y. Expression of multiple leukemic stem cell markers is associated with poor prognosis in *de novo* acute myeloid leukemia. Leuk Lymphoma. (2018) 59:2144–51. 10.1080/10428194.2017.1410888 29251166

[395] Al-Mawali A Gillis D Lewis I. Immunoprofiling of leukemic stem cells CD34+/CD38-/CD123+ delineate FLT3/ITD-positive CLones. J Hematol Oncol. (2016) 9:61. 10.1186/s13045-016-0292-z 27465508 PMC4964068

[396] Jordan CT Upchurch D Szilvassy SJ Guzman ML Howard DS Pettigrew AL. The interleukin-3 receptor alpha chain is a unique marker for human acute myelogenous leukemia stem cells. Leukemia. (2000) 14:1777–84. 10.1038/sj.leu.2401903 11021753

[397] Xie LH Biondo M Busfield SJ Arruda A Yang X Vairo G. CD123 target validation and preClinical evaluation of ADCC activity of anti-CD123 antibody CSL362 in combination with NKs from AML patients in remission. Blood Cancer J. (2017) 7:e567. 10.1038/bcj.2017.52 28574487 PMC5520399

[398] Bras AE Haas V de van Stigt A Jongen-Lavrencic M Beverloo HB Te Marvelde JG. CD123 expression levels in 846 acute leukemia patients based on standardized immunophenotyping. Cytometry B Clin Cytom. (2019) 96:134–42. 10.1002/cyto.b.21745 30450744 PMC6587863

[399] Arai N Homma M Abe M Baba Y Murai S Watanuki M. Impact of CD123 expression, analyzed by immunohistochemistry, on Clinical outcomes in patients with acute myeloid leukemia. Int J Hematol. (2019) 109:539–44. 10.1007/s12185-019-02616-y 30847774

[400] Bonifant CL Szoor A Torres D Joseph N Velasquez MP Iwahori K. CD123-engager T cells as a novel immunotherapeutic for acute myeloid leukemia. Mol Ther. (2016) 24:1615–26. 10.1038/mt.2016.116 27401038 PMC5113097

[401] Li F Sutherland MK Yu C Walter RB Westendorf L Valliere-Douglass J. Characterization of SGN-CD123A, a potent CD123-directed antibody-drug conjugate for acute myeloid leukemia. Mol Cancer Ther. (2018) 17:554–64. 10.1158/1535-7163.MCT-17-0742 29142066

[402] Kovtun Y Jones GE Adams S Harvey L Audette CA Wilhelm A. A CD123-targeting antibody-drug conjugate, IMGN632, designed to eradicate AML while sparing normal bone marrow cells. Blood Adv. (2018) 2:848–58. 10.1182/bloodadvances.2018017517 29661755 PMC5916008

[403] Han L Jorgensen JL Brooks C Shi C Zhang Q Nogueras González GM. Antileukemia efficacy and mechanisms of action of SL-101, a novel anti-CD123 antibody conjugate, in acute myeloid leukemia. Clin Cancer Res. (2017) 23:3385–95. 10.1158/1078-0432.CCR-16-1904 28096272 PMC5496806

[404] Fan D Li Z Zhang X Yang Y Yuan X Zhang X. AntiCD3Fv fused to human interleukin-3 deletion variant redirected T cells against human acute myeloid leukemic stem cells. J Hematol Oncol. (2015) 8:18. 10.1186/s13045-015-0109-5 25879549 PMC4389834

[405] Busfield SJ Biondo M Wong M Ramshaw HS Lee EM Ghosh S. Targeting of acute myeloid leukemia *in vitro* and *in vivo* with an anti-CD123 mAb engineered for optimal ADCC. Leukemia. (2014) 28:2213–21. 10.1038/leu.2014.128 24705479

[406] Williams BA Wang X-H Leyton JV Maghera S Deif B Reilly RM. CD16+NK-92 and anti-CD123 monoclonal antibody prolongs survival in primary human acute myeloid leukemia xenografted mice. Haematologica. (2018) 103:1720–9. 10.3324/haematol.2017.187385 29976748 PMC6165813

[407] Hutmacher C Volta L Rinaldi F Murer P Myburgh R Manz MG. Development of a novel fully-human anti-CD123 antibody to target acute myeloid leukemia. Leuk Res. (2019) 84:106178. 10.1016/j.leukres.2019.106178 31326578

[408] Thokala R Olivares S Mi T Maiti S Deniger D Huls H. Redirecting specificity of t cells using the sleeping beauty system to express chimeric antigen receptors by mix-and-matching of VL and VH domains targeting CD123+ tumors. PLoS ONE. (2016) 11:e0159477. 10.1371/jouRNAl.pone.0159477 27548616 PMC4993583

[409] Oberschmidt O Morgan M Huppert V Kessler J Gardlowski T Matthies N. Development of automated separation, expansion, and quality control protocols for clinical-scale manufacturing of primary human NK cells and alpharetroviral chimeric antigen receptor engineering. Hum Gene Ther Methods. (2019) 30:102–20. 10.1089/hgtb.2019.039 30997855 PMC6590729

[410] Tasian SK Kenderian SS Shen F Ruella M Shestova O Kozlowski M. Optimized depletion of chimeric antigen receptor T cells in murine xenograft models of human acute myeloid leukemia. Blood. (2017) 129:2395–407. 10.1182/blood-2016-08-736041 28246194 PMC5409446

[411] He SZ Busfield S Ritchie DS Hertzberg MS Durrant S Lewis ID A phase 1 study of the safety, pharmacokinetics and anti-leukemic activity of the anti-CD123 monoclonal antibody CSL360 in relapsed, refractory or high-risk acute myeloid leukemia. Leuk Lymphoma. (2015) 56:1406–15. 10.3109/10428194.2014.956316 25248882

[412] Al-Hussaini M Rettig MP Ritchey JK Karpova D Uy GL Eissenberg LG. Targeting CD123 in acute myeloid leukemia using a T-cell-directed dual-affinity retargeting platform. Blood. (2016) 127:122–31. 10.1182/blood-2014-05-575704 26531164 PMC4705603

[413] Zahran AM Aly SS Rayan A El-Badawy O Fattah MA Ali AM. Survival outcomes of CD34+CD38-LSCs and their expression of CD123 in adult AML patients. Oncotarget. (2018) 9:34056–65. 10.18632/oncotarget.26118 30344921 PMC6183348

[414] Jiang Y-P Liu BY Zheng Q Panuganti S Chen R Zhu J. CLT030, a leukemic stem cell-targeting CLL1 antibody-drug conjugate for treatment of acute myeloid leukemia. Blood Adv. (2018) 2:1738–49. 10.1182/bloodadvances.2018020107 30037800 PMC6058235

[415] van Rhenen A van Dongen GA Kelder A Rombouts EJ Feller N Moshaver B. The novel AML stem cell associated antigen CLL-1 aids in discrimination between normal and leukemic stem cells. Blood. (2007) 110:2659–66. 10.1182/blood-2007-03-083048 17609428

[416] Bill M Aggerholm A Kjeldsen E Roug AS Hokland P Nederby L. Revisiting CLEC12A as leukaemic stem cell marker in AML: highlighting the necessity of precision diagnostics in patients eligible for targeted therapy. Br J Haematol. (2019) 184:769–81. 10.1111/bjh.15711 30520015

[417] Leong SR Sukumaran S Hristopoulos M Totpal K Stainton S Lu E. An anti-CD3/anti–CLL-1 bispecific antibody for the treatment of acute myeloid leukemia. Blood. (2017) 129:609–18. 10.1182/blood-2016-08-735365 27908880 PMC5290988

[418] Lin TY Zhu Y Li Y Zhang H Ma AH Long Q. Daunorubicin-containing CLL1-targeting nanomicelles have anti-leukemia stem cell activity in acute myeloid leukemia. Nanomedicine. (2019) 20:102004. 10.1016/j.nano.2019.04.007 31055076 PMC8237247

[419] Kenderian SS Ruella M Shestova O Klichinsky M Kim M Soderquist C Targeting CLEC12A with chimeric antigen receptor T Cells can overcome the chemotherapy refractoriness of leukemia stem cells. Biol. Blood Marrow Transpl. (2017) 23:S247–8. 10.1016/j.bbmt.2016.12.413

[420] Darwish NHE Sudha T Godugu K Elbaz O Abdelghaffar HA Hassan EEA. Acute myeloid leukemia stem cell markers in prognosis and targeted therapy: potential impact of BMI-1, TIM-3 and CLL-1. Oncotarget. (2016) 7:57811–20. 10.18632/oncotarget.11063 27506934 PMC5295391

[421] Kikushige Y Shima T Takayanagi S-i Urata S Miyamoto T Iwasaki H. TIM-3 is a promising target to selectively kill acute myeloid leukemia stem cells. Cell Stem Cell. (2010) 7:708–17. 10.1016/j.stem.2010.11.014 21112565

[422] Homayouni V Ganjalikhani-hakemi M Rezaei A Khanahmad H Behdani M Lomedasht FK. Preparation and characterization of a novel nanobody against T-cell immunoglobulin and mucin-3 (TIM-3). Iran J Basic Med Sci. (2016) 19:1201–8. 10.15171/ijb.1427 27917276 PMC5126221

[423] Dama P Tang M Fulton N Kline J Liu H. Gal9/TIM-3 expression level is higher in AML patients who fail chemotherapy. J. ImmunoTher Cancer. (2019) 7:175. 10.1186/s40425-019-0611-3 31291985 PMC6621946

[424] van Hoang T Buss EC Wang W Hoffmann I Raffel S Zepeda-Moreno A. The rarity of ALDH(+) cells is the key to separation of normal versus leukemia stem cells by ALDH activity in AML patients. Int J Cancer. (2015) 137:525–36. 10.1002/ijc.29410 25545165 PMC4755039

[425] Blume R Rempel E Manta L Saeed BR Wang W Raffel S. The molecular signature of AML with increased ALDH activity suggests a stem cell origin. Leuk Lymphoma. (2018) 59:2201–10. 10.1080/10428194.2017.1422862 29334844

[426] Voeltzel T Flores-Violante M Zylbersztejn F Lefort S Billandon M Jeanpierre S. A new signaling cascade linking BMP4, BMPR1A, ΔNp73 and NANOG impacts on stem-like human cell properties and patient outcome. Cell Death Dis. (2018) 9:1011. 10.1038/s41419-018-1042-7 30262802 PMC6160490

[427] Kakiuchi S Minami Y Miyata Y Mizutani Y Goto H Kawamoto S. NANOG expression as a responsive biomarker during treatment with hedgehog signal inhibitor in acute myeloid leukemia. Int J Mol Sci. (2017) 18:486. 10.3390/ijms18030486 28245563 PMC5372502

[428] Picot T Kesr S Wu Y Aanei CM Flandrin-Gresta P Tondeur S. Potential role of OCT4 in leukemogenesis. Stem Cells Dev. (2017) 26:1637–47. 10.1089/scd.2017.0134 28911263

[429] Yin J-Y Tang Q Zhai L-L Zhou L-Y Qian J Lin J. High expression of OCT4 is frequent and may cause undesirable treatment outcomes in patients with acute myeloid leukemia. Tumour Biol. (2015) 36:9711–6. 10.1007/s13277-015-3731-5 26152287

[430] Xiang Y Zhou X. Octamer-binding transcription factor 4 correlates with complex karyotype, fLT3-ITD mutation and poorer risk stratification, and predicts unfavourable prognosis in patients with acute myeloid leukaemia. Hematology. (2018) 23:721–8. 10.1080/10245332.2018.1482050 29950146

[431] Zhang L-Y Yuan Y-Q Zhou D-M Wang Z-Y Ju S-G Sun Y Impact of global and gene-specific DNA methylation in *de novo* or relapsed acute myeloid leukemia patients treated with decitabine. Asian Pac J Cancer Prev. (2016) 17:431–7. 10.7314/APJCP.2016.17.1.431 26838251

[432] Tosic N Petrovic I Grujicic NK Davidovic S Virijevic M Vukovic NS. Prognostic significance of SOX2, SOX3, SOX11, SOX14 and SOX18 gene expression in adult *de novo* acute myeloid leukemia. Leuk Res. (2018) 67:32–8. 10.1016/j.leukres.2018.02.001 29428447

[433] Sadovnik I Hoelbl-Kovacic A Herrmann H Eisenwort G Cerny-Reiterer S Warsch W. Identification of CD25 as STAT5-Dependent growth-Regulator of leukemic stem cells in PH+ CML. Clin Cancer Res. (2015) 22:2051–61. 10.1158/1078-0432.CCR-15-0767 26607600 PMC4817228

[434] Herrmann H Sadovnik I Cerny-Reiterer S Rülicke T Stefanzl G Willmann M. Dipeptidylpeptidase IV (CD26) defines leukemic stem cells (LSC) in chronic myeloid leukemia. Blood. (2014) 123:3951–62. 10.1182/blood-2013-10-536078 24778155

[435] Landberg N Palffy S von Askmyr M Lilljebjörn H Sandén C Rissler M. CD36 defines primitive chronic myeloid leukemia cells less responsive to imatinib but vulnerable to antibody-based therapeutic targeting. Haematologica. (2018) 103:447–55. 10.3324/haematol.2017.169946 29284680 PMC5830390

[436] Warfvinge R Geironson L Sommarin MN Lang S Karlsson C Roschupkina T. Single-cell molecular analysis defines therapy response and immunophenotype of stem cell subpopulations in CML. Blood. (2017) 129:2384–94. 10.1182/blood-2016-07-728873 28122740 PMC5484462

[437] Landberg N Hansen N Askmyr M Ågerstam H Lassen C Rissler M. IL1RAP expression as a measure of leukemic stem cell burden at diagnosis of chronic myeloid leukemia predicts therapy outcome. Leukemia. (2016) 30:255–8. 10.1038/leu.2015.135 26067823

[438] Houshmand M Simonetti G Circosta P Gaidano V Cignetti A Martinelli G. Chronic myeloid leukemia stem cells. Leukemia. (2019) 33:1543–56. 10.1038/s41375-019-0490-0 31127148 PMC6755964

[439] Sadovnik I Herrmann H Eisenwort G Blatt K Hoermann G Mueller N. Expression of CD25 on leukemic stem cells in BCR-ABL1+ CML: potential diagnostic value and functional implications. Exp Hematol. (2017) 51:17–24. 10.1016/j.exphem.2017.04.003 28457753 PMC6044418

[440] Valent P Sadovnik I Eisenwort G Bauer K Herrmann H Gleixner KV. Immunotherapy-Based targeting and elimination of leukemic stem cells in AML and CML. Int J Mol Sci. (2019) 20. 10.3390/ijms20174233 31470642 PMC6747233

[441] Madhumathi J Sridevi S Verma RS. CD25 targeted therapy of chemotherapy resistant leukemic stem cells using DR5 specific TRAIL peptide. Stem Cell Res. (2017) 19:65–75. 10.1016/j.scr.2017.01.001 28076753

[442] Bocchia M Sicuranza A Abruzzese E Iurlo A Sirianni S Gozzini A. Residual peripheral blood CD26+ leukemic stem cells in chronic myeloid leukemia patients during TKI therapy and during treatment-free remission. Front. Oncol. (2018) 8:194. 10.3389/fonc.2018.00194 29900128 PMC5988870

[443] Culen M Borsky M Nemethova V Razga F Smejkal J Jurcek T. Quantitative assessment of the CD26+ leukemic stem cell compartment in chronic myeloid leukemia: patient-subgroups, prognostic impact, and technical aspects. Oncotarget. (2016) 7:33016–24. 10.18632/oncotarget.9108 27145281 PMC5078071

[444] Galimberti S Grassi S Baratè C Guerrini F Ciabatti E Perutelli F. The polycomb BMI1 protein is co-expressed with CD26+ in leukemic stem cells of chronic myeloid leukemia. Front. Oncol. (2018) 8:555. 10.3389/fonc.2018.00555 30574454 PMC6291509

[445] Zhou S Zhu X Liu W Cheng F Zou P You Y. Comparison of chronic myeloid leukemia stem cells and hematopoietic stem cells by global proteomic analysis. Biochem Biophys Res Commun. (2020) 522:362–7. 10.1016/j.bbrc.2019.11.092 31767149

[446] Raspadori D Pacelli P Sicuranza A Abruzzese E Iurlo A Cattaneo D. Flow cytometry assessment of CD26+ leukemic stem cells in peripheral blood: a simple and rapid new diagnostic tool for chronic myeloid leukemia. Cytometry B Clin Cytom. (2019) 96:294–9. 10.1002/cyto.b.21764 30714299 PMC6767040

[447] Willmann M Sadovnik I Eisenwort G Entner M Bernthaler T Stefanzl G. Evaluation of cooperative antileukemic effects of nilotinib and vildagliptin in Ph+ chronic myeloid leukemia. Exp Hematol. (2018) 57:50–9.e6. 10.1016/j.exphem.2017.09.012 29031704 PMC7115814

[448] Zhou S Li W Xiao Y Zhu X Zhong Z Li Q. A novel chimeric antigen receptor redirecting T-cell specificity towards CD26+ cancer cells. Leukemia. (2020). 10.1038/s41375-020-0824-y 32317776

[449] Frolova O Benito J Brooks C Wang R-Y Korchin B Rowinsky EK. SL-401 and SL-501, targeted therapeutics directed at the interleukin-3 receptor, inhibit the growth of leukaemic cells and stem cells in advanced phase chronic myeloid leukaemia. Br J Haematol. (2014) 166:862–74. 10.1111/bjh.12978 24942980 PMC4146738

[450] Nievergall E Ramshaw HS Yong AS Biondo M Busfield SJ Vairo G. Monoclonal antibody targeting of IL-3 receptor α with CSL362 effectively depletes CML progenitor and stem cells. Blood. (2014) 123:1218–28. 10.1182/blood-2012-12-475194 24363400

[451] Zhang J Zhao A Sun L Chen W Zhang H Chen Z. Selective surface marker and miRNA profiles of CD34+ blast-derived microvesiCLes in chronic myelogenous leukemia. Oncol Lett. (2017) 14:1866–74. 10.3892/ol.2017.6336 28789422 PMC5529767

[452] Ågerstam H Hansen N Palffy S von Sandén C Reckzeh K Karlsson C. IL1RAP antibodies block IL-1-induced expansion of candidate CML stem cells and mediate cell killing in xenograft models. Blood. (2016) 128:2683–93. 10.1182/blood-2015-11-679985 27621309

[453] Warda W Larosa F Neto Da Rocha M Trad R Deconinck E Fajloun Z. CML hematopoietic stem cells expressing IL1RAP can be targeted by chimeric antigen receptor-engineered T Cells. Cancer Res. (2019) 79:663–75. 10.1158/0008-5472.CAN-18-1078 30514753

[454] Zhou H Mak PY Mu H Mak DH Zeng Z Cortes J. Combined inhibition of β-catenin and Bcr-Abl synergistically targets tyrosine kinase inhibitor-resistant blast crisis chronic myeloid leukemia blasts and progenitors *in vitro* and *in vivo*. Leukemia. (2017) 31:2065–74. 10.1038/leu.2017.87 28321124 PMC5628102

[455] Jing Hu Min Feng Zhang-Ling Liu Yi Liu Zheng-Lan Huang Hui Li. Potential role of Wnt/β-catenin signaling in blastic transformation of chronic myeloid leukemia: cross talk between β-catenin and BCR-ABL. Tumor Biol. (2016) 37:15859–72. 10.1007/s13277-016-5413-3 27817074

[456] de Cássia Viu Carrara R Fontes AM Abraham KJ Orellana MD Haddad SK Palma PVB Expression differences of genes in the PI3K/AKT, WNT/b-catenin, SHH, NOTCH and MAPK signaling pathways in CD34+ hematopoietic cells obtained from chronic phase patients with chronic myeloid leukemia and from healthy controls. Clin Transl Oncol. (2018) 20:542–9. 10.1007/s12094-017-1751-x 28905209

[457] Masamoto Y Kurokawa M. Targeting chronic myeloid leukemia stem cells: can transcriptional program be a druggable target for cancers? Stem Cell Investig. (2018) 5:10. 10.21037/sci.2018.03.05 29780814 PMC5945907

[458] Zhao Y Masiello D McMillian M Nguyen C Wu Y Melendez E. CBP/catenin antagonist safely eliminates drug-resistant leukemia-initiating cells. Oncogene. (2016) 35:3705–17. 10.1038/onc.2015.438 26657156 PMC5526055

[459] Jin B Wang C Li J Du X Ding K Pan J. Anthelmintic niclosamide disrupts the interplay of p65 and FOXM1/beta-catenin and eradicates leukemia stem cells in chronic myelogenous leukemia. Clin Cancer Res. (2017) 23:789–803. 10.1158/1078-0432.CCR-16-0226 27492973

[460] Pellicano F Scott MT Helgason GV Hopcroft LE Allan EK Aspinall-O'Dea M. The antiproliferative activity of kinase inhibitors in chronic myeloid leukemia cells is mediated by FOXO transcription factors. Stem Cells. (2014) 32:2324–37. 10.1002/stem.1748 24806995 PMC4282530

[461] Sadarangani A Pineda G Lennon KM Chun H-J Shih A Schairer AE. GLI2 inhibition abrogates human leukemia stem cell dormancy. J Transl Med. (2015) 13:98. 10.1186/s12967-015-0453-9 25889765 PMC4414375

[462] Cui J Li P Liu X Hu H Wei W. Abnormal expression of the notch and Wnt/β-catenin signaling pathways in stem-like ALDHHI CD44+ cells correlates highly with Ki-67 expression in breast cancer. Oncol Lett. (2015) 9:1600–6. 10.3892/ol.2015.2942 25789008 PMC4356390

[463] Ponta H Sherman L Herrlich PA. CD44: from adhesion molecules to signalling regulators. Nat Rev Mol Cell Biol. (2003) 4:33–45. 10.1038/nrm1004 12511867

[464] Akamine T Tagawa T Ijichi K Toyokawa G Takamori S Hirai F. The significance of CD44 variant 9 in resected lung adenocarcinoma: correlation with pathological early-stage and EGFR mutation. Ann Surg Oncol. (2019) 26:1544–51. 10.1245/s10434-018-07137-2 30798450

[465] Li M Zhang B Zhang Z Liu X Qi X Zhao J. Stem cell-like circulating tumor cells indicate poor prognosis in gastric cancer. Biomed Res Int. (2014) 2014:981261. 10.1155/2014/981261 24963492 PMC4054962

[466] Stroomer JW Roos JC Sproll M Quak JJ Heider KH Wilhelm BJ. Safety and biodistribution of 99mTechnetium-labeled anti-CD44v6 monoclonal antibody BIWA 1 in head and neck cancer patients. Clin Cancer Res. (2000) 6:3046–55. 10955783

[467] Börjesson PK Postema EJ Roos JC Colnot DR Marres HA van Schie MH. Phase I therapy study with 186Re-labeled humanized monoclonal antibody BIWA 4 (Bivatuzumab) in patients with head and neck squamous cell carcinoma. Clin Cancer Res. (2003) 9(10 Pt 2):3961–72s. 14506195

[468] Menke-van der Houven van Oordt CW Gomez-Roca C van Herpen C Coveler AL Mahalingam D Verheul HMW. First-in-human phase I clinical trial of RG7356, an anti-CD44 humanized antibody, in patients with advanced, CD44-expressing solid tumors. Oncotarget. (2016) 7:80046–58. 10.18632/oncotarget.11098 27507056 PMC5346770

[469] Li Z. CD133: a stem cell biomarker and beyond. Exp Hematol Oncol. (2013) 2:17. 10.1186/2162-3619-2-17 23815814 PMC3701589

[470] Ren F Sheng WQ Du X. CD133: a cancer stem cells marker, is used in colorectal cancers. World J Gastroenterol. (2013) 19:2603–11. 10.3748/wjg.v19.i17.2603 23674867 PMC3645378

[471] Gazzaniga P Gradilone A Petracca A Nicolazzo C Raimondi C Iacovelli R. Molecular markers in circulating tumour cells from metastatic colorectal cancer patients. J Cell Mol Med. (2010) 14:2073–7. 10.1111/j.1582-4934.2010.01117.x 20597995 PMC3822998

[472] Xia P. CD133 mRNA may be a suitable prognostic marker for human breast cancer. Stem Cell Investig. (2017) 4:87. 10.21037/sci.2017.10.03 29270413 PMC5723734

[473] Trzpis M MCLaughlin PM Leij LM de Harmsen MC. Epithelial cell adhesion molecule: more than a carcinoma marker and adhesion molecule. Am J Pathol. (2007) 171:386–95. 10.2353/ajpath.2007.070152 17600130 PMC1934518

[474] Cirulli V Crisa L Beattie GM Mally MI Lopez AD Fannon A. KSA antigen Ep-CAM mediates cell-cell adhesion of pancreatic epithelial cells: morphoregulatory roles in pancreatic islet development. J Cell Biol. (1998) 140:1519–34. 10.1083/jcb.140.6.1519 9508783 PMC2132663

[475] Huang L Yang Y Yang F Liu S Zhu Z Lei Z. Functions of EpCAM in physiological processes and diseases (review). Int J Mol Med. (2018) 42:1771–85. 10.3892/ijmm.2018.3764 30015855 PMC6108866

[476] Balzar M Winter MJ Boer CJ de Litvinov SV. The biology of the 17-1A antigen (Ep-CAM). J Mol Med. (1999) 77:699–712. 10.1007/s001099900038 10606205

[477] Schmelzer E Zhang L Bruce A Wauthier E Ludlow J Yao H-l. Human hepatic stem cells from fetal and postnatal donors. J Exp Med. (2007) 204:1973–87. 10.1084/jem.20061603 17664288 PMC2118675

[478] Kamimoto K Kaneko K Kok CY-Y Okada H Miyajima A Itoh T. Heterogeneity and stochastic growth regulation of biliary epithelial cells dictate dynamic epithelial tissue remodeling. Elife. (2016) 5:15034. 10.7554/eLife.15034 27431614 PMC4951195

[479] Schnell U Cirulli V Giepmans BN. EpCAM: structure and function in health and disease. Biochim Biophys Acta. (2013) 1828:1989–2001. 10.1016/j.bbamem.2013.04.018 23618806

[480] Hiraga T Ito S Nakamura H. EpCAM expression in breast cancer cells is associated with enhanced bone metastasis formation. Int J Cancer. (2016) 138:1698–708. 10.1002/ijc.29921 26576938

[481] Kim J Orkin SH. Embryonic stem cell-specific signatures in cancer: insights into genomic regulatory networks and implications for medicine. Genome Med. (2011) 3:75. 10.1186/gm291 22126538 PMC3308030

[482] Hadjimichael C Chanoumidou K Papadopoulou N Arampatzi P Papamatheakis J Kretsovali A. Common stemness regulators of embryonic and cancer stem cells. World J Stem Cells. (2015) 7:1150–84. 10.4252/wjsc.v7.i9.1150 26516408 PMC4620423

[483] Rosner MH Vigano MA Ozato K TIMmons PM Poirier F Rigby PW. A POU-domain transcription factor in early stem cells and germ cells of the mammalian embryo. Nature. (1990) 345:686–92. 10.1038/345686a0 1972777

[484] Carrasco-Garcia E Santos JC Garcia I Brianti M García-Puga M Pedrazzoli J. Paradoxical role of SOX2 in gastric cancer. Am J Cancer Res. (2016) 6:701–13. 27186426 PMC4859879

[485] Wang Y-J Herlyn M. The emerging roles of Oct4 in tumor-initiating cells. Am J Physiol Cell Physiol. (2015) 309:C709–18. 10.1152/ajpcell.00212.2015 26447206 PMC4725440

[486] Dai X Ge J Wang X Qian X Zhang C Li X. OCT4 regulates epithelial-mesenchymal transition and its knockdown inhibits colorectal cancer cell migration and invasion. Oncol Rep. (2013) 29:155–60. 10.3892/or.2012.2086 23076549

[487] Jeter CR Yang T Wang J Chao H-P Tang DG. Concise review: NANOG in cancer stem cells and tumor development: an update and outstanding questions. Stem Cells. (2015) 33:2381–90. 10.1002/stem.2007 25821200 PMC4509798

[488] Quéré R Andradottir S Brun AC Zubarev RA Karlsson G Olsson K. High levels of the adhesion molecule CD44 on leukemic cells generate acute myeloid leukemia relapse after withdrawal of the initial transforming event. Leukemia. (2010) 25:515–26. 10.1038/leu.2010.281 21116281 PMC3072510

[489] Wang N-S Wei M Ma W-l Meng W Zheng W-l. Knockdown of CD44 enhances chemosensitivity of acute myeloid leukemia cells to ADM Ara-C. Tumour Biol. (2014) 35:3933–40. 10.1007/s13277-013-1523-3 24375249

[490] Huang X Li D Li T Zhao BO Chen X. Prognostic value of the expression of phosphatase and tensin homolog and CD44 in elderly patients with refractory acute myeloid leukemia. Oncol Lett. (2015) 10:103–10. 10.3892/ol.2015.3189 26170984 PMC4486892

[491] Zhou H-S Carter BZ Andreeff M. Bone marrow niche-mediated survival of leukemia stem cells in acute myeloid leukemia: Yin and Yang. Cancer Biol Med. (2016) 13:248–59. 10.20892/j.issn.2095-3941.2016.0023 27458532 PMC4944541

[492] Testa U Pelosi E Castelli G. CD123 as a therapeutic target in the treatment of hematological malignancies. Cancers. (2019) 11:1358. 10.3390/cancers11091358 31547472 PMC6769702

[493] Pollyea DA Jordan CT. Therapeutic targeting of acute myeloid leukemia stem cells. Blood. (2017) 129:1627–35. 10.1182/blood-2016-10-696039 28159738

[494] Wittwer NL Brumatti G Marchant C Sandow JJ Pudney MK Dottore M. High CD123 levels enhance proliferation in response to IL-3, but reduce chemotaxis by downregulating CXCR4 expression. Blood Adv. (2017) 1:1067–79. 10.1182/bloodadvances.2016002931 29296749 PMC5728309

[495] Faez A Subh SA-M. Impact of leukemia stem cells phenotype expression on response to induction therapy in acute myeloid leukemia patients. Cardiovasc Hematol Disord Drug Targets. (2020) 20:145–151. 10.2174/1871529X19666190719105954 31438833

[496] Zhou J Chng W-J. Identification and targeting leukemia stem cells: the path to the cure for acute myeloid leukemia. World J Stem Cells. (2014) 6:473–84. 10.4252/wjsc.v6.i4.473 25258669 PMC4172676

[497] Davis JR Benjamin DJ Jonas BA. New and emerging therapies for acute myeloid leukaemia. J Investig Med. (2018) 66:1088–95. 10.1136/jim-2018-000807 30127098 PMC6733983

[498] Kikushige Y Miyamoto T. Identification of TIM-3 as a leukemic stem cell surface molecule in primary acute myeloid leukemia. Oncology. (2015) 89(Suppl. 1):28–32. 10.1159/000431062 26551150

[499] Grainger S Traver D Willert K. Wnt signaling in hematological malignancies. Prog Mol Biol Transl Sci. (2018) 153:321–41. 10.1016/bs.pmbts.2017.11.002 29389522 PMC5972548

[500] Zhao Y Wu K Wu Y Melendez E Smbatyan G Massiello D Characterization of imatinib resistant CML leukemic stem/Initiating cells and their sensitivity to CBP/Catenin antagonists. Curr Mol Pharmacol. (2018) 11:113–21. 10.2174/1874467210666170919155739 28933312

[501] Blaudszun A-R Moldenhauer G Schneider M Philippi A. A photosensitizer delivered by bispecific antibody redirected T lymphocytes enhances cytotoxicity against EpCAM-expressing carcinoma cells upon light irradiation. J Control Release. (2015) 197:58–68. 10.1016/j.jconrel.2014.10.025 25449805

[502] Hosoya N Miyagawa K. Targeting DNA damage response in cancer therapy. Cancer Sci. (2014) 105:370–88. 10.1111/cas.12366 24484288 PMC4317796

[503] Ruhland MK Coussens LM Stewart SA. Senescence and cancer: an evolving inflammatory paradox. Biochim Biophys Acta. (2016) 1865:14–22. 10.1016/j.bbcan.2015.10.001 26453912 PMC4733607

[504] Ewald JA Desotelle JA Wilding G Jarrard DF. Therapy-induced senescence in cancer. J Natl Cancer Inst. (2010) 102:1536–46. 10.1093/jnci/djq364 20858887 PMC2957429

[505] Dodig S Cepelak I Pavić I. Hallmarks of senescence and aging. Biochem Med. (2019) 29:030501. 10.11613/BM.2019.030501 31379458 PMC6610675

[506] Lee S Lee J-S. Cellular senescence: a promising strategy for cancer therapy. BMB Rep. (2019) 52:35–41. 10.5483/BMBRep.2019.52.1.294 30526771 PMC6386234

[507] Chakradeo S Elmore LW Gewirtz DA. Is senescence reversible? Curr Drug Targets. (2016) 17:460–6. 10.2174/1389450116666150825113500 26302802

[508] Zhao H Darzynkiewicz Z. Biomarkers of cell senescence assessed by imaging cytometry. Methods Mol Biol. (2013) 965:83–92. 10.1007/978-1-62703-239-1_5 23296652 PMC3541526

[509] Aljabery F Shabo I Gimm O Jahnson S Olsson H. The expression profile of p14, p53 and p21 in tumour cells is associated with disease-specific survival and the outcome of postoperative chemotherapy treatment in muscle-invasive bladder cancer. Urol Oncol. (2018) 36:530.e7-530.e18. 10.1016/j.urolonc.2018.05.025 30539751

[510] Fallah M Mohammadi H Shaki F Hosseini-Khah Z Moloudizargari M Dashti A. Doxorubicin and liposomal doxorubicin induce senescence by enhancing nuclear factor kappa B and mitochondrial membrane potential. Life Sci. (2019) 232:116677. 10.1016/j.lfs.2019.116677 31340166

[511] Hayman L Chaudhry WR Revin VV Zhelev N Bourdon J-C. What is the potential of p53 isoforms as a predictive biomarker in the treatment of cancer? Expert Rev Mol Diagn. (2019) 19:149–59. 10.1080/14737159.2019.1563484 30582376

[512] Moraes JK de Wagner VP Fonseca FP Amaral-Silva GK Farias CB de Pilar EF. Activation of BDNF/TrkB/Akt pathway is associated with aggressiveness and unfavorable survival in oral squamous cell carcinoma. Oral Dis. (2019) 25:1925–36. 10.1111/odi.13190 31498938

[513] Lin T Hou P-F Meng S Chen F Jiang T Li M-L. Emerging roles of p53 related lncRNAs in cancer progression: a systematic review. Int J Biol Sci. (2019) 15:1287–98. 10.7150/ijbs.33218 31223287 PMC6567798

[514] van den Bossche J Deben C Pauw I de Lambrechts H Hermans C Deschoolmeester V. *In vitro* study of the polo-like kinase 1 inhibitor volasertib in non-small-cell lung cancer reveals a role for the tumor suppressor p53. Mol Oncol. (2019) 13:1196–213. 10.1002/1878-0261.12477 30859681 PMC6487694

[515] Zamorano-León JJ Ballesteros S las Heras N de Alvarez-Sala L La SeRNA-Soto M de Zekri-Nechar K. Effect of pectin on the expression of proteins associated with mitochondrial biogenesis and cell senescence in HT29-human colorectal adenocarcinoma cells. Prev Nutr Food Sci. (2019) 24:187–96. 10.3746/pnf.2019.24.2.187 31328124 PMC6615348

[516] Wagner J Damaschke N Yang B Truong M Guenther C McCormick J. Overexpression of the novel senescence marker β-Galactosidase (GLB1) in prostate cancer predicts reduced PSA recurrence. PLoS ONE. (2015) 10:e0124366. 10.1371/jouRNAl.pone.0124366 25876105 PMC4398352

[517] Zhang R Chen W Adams PD. Molecular dissection of formation of senescence-Associated heterochromatin foci?†. Mol Cell Biol. (2007) 27:2343–58. 10.1128/MCB.02019-06 17242207 PMC1820509

[518] Bernadotte A Mikhelson VM Spivak IM. Markers of cellular senescence. telomere shortening as a marker of cellular senescence. Aging. (2016) 8:3–11. 10.18632/aging.100871 26805432 PMC4761709

[519] Hartman ML Sztiller-Sikorska M Czyz M. Whole-exome sequencing reveals novel genetic variants associated with diverse phenotypes of melanoma cells. Mol Carcinog. (2019) 58:588–602. 10.1002/mc.22953 30556601

[520] Long F He Y Fu H Li Y Bao X Wang Q. PreClinical characterization of SHR6390, a novel CDK 4/6 inhibitor, *in vitro* and in human tumor xenograft models. Cancer Sci. (2019) 110:1420–30. 10.1111/cas.13957 30724426 PMC6447953

[521] Knutson KL CLynes R Shreeder B Yeramian P Kemp KP Ballman K. Improved survival of HER2+ breast cancer patients treated with trastuzumab and chemotherapy is associated with host antibody immunity against the HER2 intracellular domain. Cancer Res. (2016) 76:3702–10. 10.1158/0008-5472.CAN-15-3091 27197192 PMC5594563

[522] Gunaratna RT Santos A Luo L Nagi C Lambertz I Spier M. Dynamic role of the codon 72 p53 single-Nucleotide polymorphism in mammary tumorigenesis in a humanized mouse model. Oncogene. (2019) 38:3535–50. 10.1038/s41388-018-0630-4 30651598 PMC6756019

[523] Smolle E Fink-Neuboeck N Lindenmann J Smolle-Juettner F Pichler M. The biological and clinical relevance of inhibitor of growth (ING) genes in non-Small cell lung cancer. Cancers. (2019) 11. 10.3390/cancers11081118 31390718 PMC6721451

[524] Xiang X-H Yang L Zhang X Ma X-H Miao R-C Gu J-X. Seven-senescence-associated gene signature predicts overall survival for asian patients with hepatocellular carcinoma. World J Gastroenterol. (2019) 25:1715–28. 10.3748/wjg.v25.i14.1715 31011256 PMC6465944

[525] Eggert T Wolter K Ji J Ma C Yevsa T Klotz S. Distinct functions of senescence-associated immune responses in liver tumor surveillance and tumor progression. Cancer Cell. (2016) 30:533–47. 10.1016/j.ccell.2016.09.003 27728804 PMC7789819

[526] Coppé J-P Desprez P-Y Krtolica A Campisi J. The senescence-associated secretory phenotype: the dark side of tumor suppression. Annu Rev Pathol. (2010) 5:99–118. 10.1146/annurev-pathol-121808-102144 20078217 PMC4166495

[527] Ma F Chen D Chen F Chi Y Han Z Feng X. Human umbilical cord mesenchymal stem cells promote breast cancer metastasis by interleukin-8- and interleukin-6-dependent induction of CD44(+)/CD24(-) cells. Cell Transplant. (2015) 24:2585–99. 10.3727/096368915X687462 25695620

[528] Ortiz-Montero P Londoño-Vallejo A Vernot J-P. Senescence-associated IL-6 and IL-8 cytokines induce a self- and cross-reinforced senescence/inflammatory milieu strengthening tumorigenic capabilities in the MCF-7 breast cancer cell line. Cell Commun Signal. (2017) 15:17. 10.1186/s12964-017-0172-3 28472950 PMC5418812

[529] Yamaguchi N Nakayama Y Yamaguchi N. Down-regulation of forkhead box protein A1 (FOXA1) leads to cancer stem cell-like properties in tamoxifen-resistant breast cancer cells through induction of interleukin-6. J Biol Chem. (2017) 292:8136–48. 10.1074/jbc.M116.763276 28270510 PMC5437223

[530] Peng D Tanikawa T Li W Zhao L Vatan L Szeliga W. Myeloid-Derived suppressor cells endow stem-like qualities to breast cancer cells through IL6/STAT3 and NO/NOTCH cross-talk signaling. Cancer Res. (2016) 76:3156–65. 10.1158/0008-5472.CAN-15-2528 27197152 PMC4891237

[531] Saha S Mukherjee S Khan P Kajal K Mazumdar M Manna A. Aspirin suppresses the acquisition of chemoresistance in breast cancer by disrupting an NFκB-IL6 signaling axis responsible for the generation of cancer stem cells. Cancer Res. (2016) 76:2000–12. 10.1158/0008-5472.CAN-15-1360 26842876

[532] Wolf B Krieg K Falk C Breuhahn K Keppeler H Biedermann T. Inducing differentiation of premalignant hepatic cells as a novel therapeutic strategy in hepatocarcinoma. Cancer Res. (2016) 76:5550–61. 10.1158/0008-5472.CAN-15-3453 27488521

[533] Oh K Lee O-Y Park Y Seo MW Lee D-S. IL-1β induces IL-6 production and increases invasiveness and estrogen-independent growth in a TG2-dependent manner in human breast cancer cells. BMC Cancer. (2016) 16:724. 10.1186/s12885-016-2746-7 27609180 PMC5017052

[534] Gallo M Frezzetti D Roma C Chicchinelli N Barbieri A Arra C. RANTES and IL-6 cooperate in inducing a more aggressive phenotype in breast cancer cells. Oncotarget. (2018) 9:17543–53. 10.18632/oncotarget.24784 29707128 PMC5915136

[535] Yevsa T Kang T-W Zender L. Immune surveillance of pre-cancerous senescent hepatocytes limits hepatocellular carcinoma development. Oncoimmunology. (2012) 1:398–9. 10.4161/onci.19128 22737629 PMC3382854

[536] Kang T-W Yevsa T Woller N Hoenicke L Wuestefeld T Dauch D. Senescence surveillance of pre-malignant hepatocytes limits liver cancer development. Nature. (2011) 479:547–51. 10.1038/nature10599 22080947

[537] Was H Barszcz K Czarnecka J Kowalczyk A Bernas T Uzarowska E . Bafilomycin A1 triggers proliferative potential of senescent cancer cells *in vitro* and in NOD/SCID mice. Oncotarget. (2016) 8:9303–22. 10.18632/oncotarget.14066 28030837 PMC5354733

[538] Koboldt DC Fulton RS McLellan MD Schmidt H Kalicki-Veizer J McMichael JF Comprehensive molecular portraits of human breast tumours. Nature. (2012) 490:61–70. 10.1038/nature11412 23000897 PMC3465532

[539] Yamao T Yamashita Y-I Yamamura K Nakao Y Tsukamoto M Nakagawa S. Cellular senescence, represented by expression of caveolin-1, in cancer-associated fibroblasts promotes tumor invasion in pancreatic cancer. Ann Surg Oncol. (2019) 26:1552–9. 10.1245/s10434-019-07266-2 30805811

[540] Jabbour W Wion D. Biomarkers of aging associated with past treatments in breast cancer survivors: when therapy-induced pathways turn out to be potential therapeutic targets. NPJ Breast Cancer. (2018) 4:1–2. 10.1038/s41523-018-0058-6 29532007 PMC5840380

[541] Di G-H Liu Y Lu Y Liu J Wu C Duan H-F. IL-6 secreted from senescent mesenchymal stem cells promotes proliferation and migration of breast cancer cells. PLoS ONE. (2014) 9:e113572. 10.1371/jouRNAl.pone.0113572 25419563 PMC4242635

[542] Marotta LL Almendro V Marusyk A Shipitsin M Schemme J Walker SR. The JAK2/STAT3 signaling pathway is required for growth of CD44+CD24− stem cell-like breast cancer cells in human tumors. J Clin Invest. (2011) 121:2723–35. 10.1172/JCI44745 21633165 PMC3223826

[543] Wang T Song P Zhong T Wang X Xiang X Liu Q. The inflammatory cytokine IL-6 induces FRA1 deacetylation promoting colorectal cancer stem-like properties. Oncogene. (2019) 38:4932–47. 10.1038/s41388-019-0763-0 30804456 PMC6756002

[544] Dou Z Berger SL. Senescence elicits stemness: a surprising mechanism for cancer relapse. Cell Metab. (2018) 27:710–1. 10.1016/j.cmet.2018.03.009 29617638 PMC7205594

[545] Poltavets V Kochetkova M Pitson SM Samuel MS. The role of the extracellular matrix and its molecular and cellular regulators in cancer cell plasticity. Front. Oncol. (2018) 8:431. 10.3389/fonc.2018.00431 30356678 PMC6189298

[546] Aloia L McKie MA Huch M. Cellular plasticity in the adult liver and stomach. J Physiol. (2016) 594:4815–25. 10.1113/JP271769 27028579 PMC5009796

[547] Chen Y Wong PP Sjeklocha L Steer CJ Sahin MB. Mature hepatocytes exhibit unexpected plasticity by direct dedifferentiation into liver progenitor cells in culture. Hepatology. (2012) 55:563–74. 10.1002/hep.24712 21953633 PMC3268884

[548] Chaffer CL Marjanovic ND Lee T Bell G Kleer CG Reinhardt F. Poised chromatin at the ZEB1 promoter enables breast cancer cell plasticity and enhances tumorigenicity. Cell. (2013) 154:61–74. 10.1016/j.cell.2013.06.005 23827675 PMC4015106

[549] Akunuru S James Zhai Q Zheng Y. Non-small cell lung cancer stem/progenitor cells are enriched in multiple distinct phenotypic subpopulations and exhibit plasticity. Cell Death Dis. (2012) 3:e352. 10.1038/cddis.2012.93 22825470 PMC3406592

[550] Vassalli G. Aldehyde dehydrogenases: not just markers, but functional regulators of stem cells. Stem Cells Int. (2019) 2019:3904645. 10.1155/2019/3904645 30733805 PMC6348814

[551] Clark DW Palle K. Aldehyde dehydrogenases in cancer stem cells: potential as therapeutic targets. Ann Transl Med. (2016) 4:518. 10.21037/atm.2016.11.82 28149880 PMC5233526

[552] Doherty MR Parvani JG Tamagno I Junk DJ Bryson BL Cheon HJ. The opposing effects of interferon-beta and oncostatin-M as regulators of cancer stem cell plasticity in triple-negative breast cancer. Breast Cancer Res. (2019) 21:1–12. 10.1186/s13058-019-1136-x 31036052 PMC6489282

[553] Pereira L Mariadason JM Hannan RD Dhillon AS. Implications of epithelial-mesenchymal plasticity for heterogeneity in colorectal cancer. Front. Oncol. (2015) 5:13. 10.3389/fonc.2015.00013 25699236 PMC4313606

[554] Zhan T Ambrosi G Wandmacher AM Rauscher B Betge J Rindtorff N. MEK inhibitors activate Wnt signalling and induce stem cell plasticity in colorectal cancer. Nat Commun. (2019) 10:2197. 10.1038/s41467-019-09898-0 31097693 PMC6522484

[555] Legge DN Shephard AP Collard TJ Greenhough A Chambers AC CLarkson RW. BCL-3 promotes a cancer stem cell phenotype by enhancing β-catenin signalling in colorectal tumour cells. Dis Model Mech. (2019) 12:037697. 10.1242/dmm.037697 30792270 PMC6451435

[556] Liu D Du L Chen D Ye Z Duan H Tu T. Reduced CD146 expression promotes tumorigenesis and cancer stemness in colorectal cancer through activating wnt/β-catenin signaling. Oncotarget. (2016) 7:40704–18. 10.18632/oncotarget.9930 27302922 PMC5130037

[557] Mohammed MK Shao C Wang J Wei Q Wang X Collier Z. Wnt/beta-catenin signaling plays an ever-expanding role in stem cell self-renewal, tumorigenesis and cancer chemoresistance. Genes Dis. (2016) 3:11–40. 10.1016/j.gendis.2015.12.004 27077077 PMC4827448

[558] Mao J Fan S Ma W Fan P Wang B Zhang J. Roles of wnt/β-catenin signaling in the gastric cancer stem cells proliferation and salinomycin treatment. Cell Death Dis. (2014) 5:e1039. 10.1038/cddis.2013.515 24481453 PMC4040703

[559] Correnti M Raggi C. Stem-like plasticity and heterogeneity of circulating tumor cells: current status and prospect challenges in liver cancer. Oncotarget. (2017) 8:7094–115. 10.18632/oncotarget.12569 27738343 PMC5351693

[560] Shibue T Weinberg RA. EMT, CSCs, and drug resistance: the mechanistic link and Clinical implications. Nat Rev Clin Oncol. (2017) 14:611–29. 10.1038/nrCLinonc.2017.44 28397828 PMC5720366

[561] Zhang D Tang DG Rycaj K. Cancer stem cells: regulation programs, immunological properties and immunotherapy. Semin Cancer Biol. (2018) 52:94–106. 10.1016/j.semcancer.2018.05.001 29752993 PMC7859848

[562] Pan Q Li Q Liu S Ning N Zhang X Xu Y. Concise review: targeting cancer stem cells using immunologic approaches. Stem Cells. (2015) 33:2085–92. 10.1002/stem.2039 25873269 PMC4478204

[563] Schmidts A Maus MV. Making CAR T cells a solid option for solid tumors. Front Immunol. (2018) 9:2593. 10.3389/fimmu.2018.02593 30467505 PMC6235951

[564] Long KB Young RM Boesteanu AC Davis MM Melenhorst JJ Lacey SF. CAR T cell therapy of non-hematopoietic malignancies: detours on the road to clinical success. Front Immunol. (2018) 9:2740. 10.3389/fimmu.2018.02740 30559740 PMC6287001

[565] Zhu X Prasad S Gaedicke S Hettich M Firat E Niedermann G. Patient-derived glioblastoma stem cells are killed by CD133-specific CAR T cells but induce the T cell aging marker CD57. Oncotarget. (2015) 6:171–84. 10.18632/oncotarget.2767 25426558 PMC4381586

[566] Hu B Zou Y Zhang L Tang J Niedermann G Firat E. Nucleofection with plasmid DNA for CRISPR/Cas9-mediated inactivation of programmed cell death protein 1 in CD133-specific CAR T Cells. Hum Gene Ther. (2019) 30:446–58. 10.1089/hum.2017.234 29706119

[567] Klapdor R Wang S Hacker U Büning H Morgan M Dörk T. Improved killing of ovarian cancer stem cells by combining a novel chimeric antigen receptor-Based immunotherapy and chemotherapy. Hum Gene Ther. (2017) 28:886–96. 10.1089/hum.2017.168 28836469

[568] Wang Y Chen M Wu Z Tong C Dai H Guo Y. CD133-directed CAR T cells for advanced metastasis malignancies: a phase I trial. Oncoimmunology. (2018) 7:e1440169. 10.1080/2162402X.2018.1440169 29900044 PMC5993480

[569] Feng K-C Guo Y-L Liu Y Dai H-R Wang Y Lv H-Y. Cocktail treatment with EGFR-specific and CD133-specific chimeric antigen receptor-modified T cells in a patient with advanced cholangiocarcinoma. J Hematol Oncol. (2017) 10:4. 10.1186/s13045-016-0378-7 28057014 PMC5217546

[570] Colnot DR Quak JJ Roos JC van Lingen A Wilhelm AJ van Kamp GJ. Phase I therapy study of 186Re-labeled chimeric monoclonal antibody U36 in patients with squamous cell carcinoma of the head and neck. J Nucl Med. (2000) 41:1999–2010. 11138685

[571] Colnot DR Ossenkoppele GJ Roos JC Quak JJ Bree Rd Börjesson PK Reinfusion of unprocessed, granulocyte colony-stimulating factor-stimulated whole blood allows dose escalation of 186Relabeled chimeric monoclonal antibody U36 radioimmunotherapy in a phase I dose escalation study. Clin Cancer Res. (2002) 8:3401–6. 12429627

[572] Vey N Delaunay J Martinelli G Fiedler W Raffoux E Prebet T. Phase I clinical study of RG7356, an anti-CD44 humanized antibody, in patients with acute myeloid leukemia. Oncotarget. (2016) 7:32532–42. 10.18632/oncotarget.8687 27081038 PMC5078031

[573] Tijink BM Buter J Bree Rd Giaccone G Lang MS Staab A A phase I dose escalation study with anti-CD44v6 bivatuzumab mertansine in patients with incurable squamous cell carcinoma of the head and neck or esophagus. Clin Cancer Res. (2006) 12:6064–72. 10.1158/1078-0432.CCR-06-0910 17062682

[574] Rupp U Schoendorf-Holland E Eichbaum M Schuetz F Lauschner I Schmidt P. Safety and pharmacokinetics of bivatuzumab mertansine in patients with CD44v6-positive metastatic breast cancer: final results of a phase I study. Anticancer Drugs. (2007) 18:477–85. 10.1097/CAD.0b013e32801403f4 17351401

[575] Riechelmann H Sauter A Golze W Hanft G Schroen C Hoermann K. Phase I trial with the CD44v6-targeting immunoconjugate bivatuzumab mertansine in head and neck squamous cell carcinoma. Oral Oncol. (2008) 44:823–9. 10.1016/j.oraloncology.2007.10.009 18203652

[576] Leuci V Casucci GM Grignani G Rotolo R Rossotti U Vigna E. CD44v6 as innovative sarcoma target for CAR-redirected CIK cells. Oncoimmunology. (2018) 7:e1423167. 10.1080/2162402X.2017.1423167 29721373 PMC5927525

[577] Zhou Y Wen P Li M Li Y Li X-A. Construction of chimeric antigen receptor-modified T cells targeting EpCAM and assessment of their anti-tumor effect on cancer cells. Mol Med Rep. (2019) 20:2355–64. 10.3892/mmr.2019.10460 31322180

[578] Armstrong A Eck SL. EpCAM: a new therapeutic target for an old cancer antigen. Cancer Biol Ther. (2003) 2:320–6. 10.4161/cbt.2.4.451 14508099

[579] Zhang B-L Li D Gong Y-L Huang Y Qin D-Y Jiang L. Preclinical evaluation of chimeric antigen receptor-modified T Cells specific to epithelial cell adhesion molecule for treating colorectal cancer. Hum Gene Ther. (2019) 30:402–12. 10.1089/hum.2018.229 30693795

[580] Zhang Q Zhang H Ding J Liu H Li H Li H. Combination therapy with EPCAM-CAR-NK-92 cells and regorafenib against human colorectal cancer models. J Immunol Res. (2018) 2018:1–11. 10.1155/2018/4263520 30410941 PMC6205314

[581] Ang WX Li Z Chi Z Du S-H Chen C Tay JCK. Intraperitoneal immunotherapy with T cells stably and transiently expressing anti-EpCAM CAR in xenograft models of peritoneal carcinomatosis. Oncotarget. (2017) 8:13545–59. 10.18632/oncotarget.14592 28088790 PMC5355119

[582] Study Evaluating Safety Efficacy of CAR-T Cells Targeting CD123 in Patients with Acute Leukemia. CLinicalTrials.gov (2020). Available online at: https://clinicaltrials.gov/ct2/show/NCT03672851?term=NCT03672851&draw=2&rank=1 (accessed April 27, 2020).

[583] Hegde M Mukherjee M Grada Z Pignata A Landi D Navai SA Tandem CAR T cells targeting HER2 and IL13Rα2 mitigate tumor antigen escape. J Clin Invest. (2016) 126:3036–52. 10.1172/JCI83416 27427982 PMC4966331

[584] Bielamowicz K Fousek K Byrd TT Samaha H Mukherjee M Aware N Trivalent CAR T cells overcome interpatient antigenic variability in glioblastoma. Neuro Oncol. (2018) 20:506–18. 10.1093/neuonc/nox182 29016929 PMC5909636

[585] Guedan S Calderon H Posey AD Maus MV. Engineering and design of chimeric antigen receptors. Mol Ther Methods Clin Dev. (2019) 12:145–56. 10.1016/j.omtm.2018.12.009 30666307 PMC6330382

[586] Minutolo NG Hollander EE Powell DJ. The emergence of universal immune receptor T cell therapy for cancer. Front Oncol. (2019) 9:176. 10.3389/fonc.2019.00176 30984613 PMC6448045

[587] MacKay M Afshinnekoo E Rub J Hassan C Khunte M Baskaran N. The therapeutic landscape for cells engineered with chimeric antigen receptors. Nature Biotechnol. (2020) 38:233–44. 10.1038/s41587-019-0329-2 31907405

[588] Bachmann M. The uni CAR system: a modular CAR T cell approach to improve the safety of CAR T cells. Immunol Lett. (2019) 211:13–22. 10.1016/j.imlet.2019.05.003 31091431

[589] Cho JH Collins JJ Wong WW. Universal chimeric antigen receptors for multiplexed and logical control of T cell responses. Cell. (2018) 173:1426–38.e11. 10.1016/j.cell.2018.03.038 29706540 PMC5984158

[590] Hübner J Hoseini SS Suerth JD Hoffmann D Maluski M Herbst J. Generation of genetically engineered precursor T-Cells from human umbilical cord blood using an optimized alpharetroviral vector platform. Mol Ther. (2016) 24:1216–26. 10.1038/mt.2016.89 27138041 PMC5088766

[591] Gargett T Brown MP. The inducible caspase-9 suicide gene system as a “safety switch” to limit on-target, off-tumor toxicities of chimeric antigen receptor T cells. Front Pharmacol. (2014) 5:235. 10.3389/fphar.2014.00235 25389405 PMC4211380

[592] Fedorov VD Themeli M Sadelain M. PD-1– and CTLA-4–based inhibitory chimeric antigen receptors (ICARs) divert off-Target immunotherapy responses. Sci Transl Med. (2013) 5:215ra172. 10.1126/scitranslmed.3006597 24337479 PMC4238416

[593] Roybal KT Rupp LJ Morsut L Walker WJ McNally KA Park JS. Precision tumor recognition by T Cells with combinatorial antigen-sensing circuits. Cell. (2016) 164:770–9. 10.1016/j.cell.2016.01.011 26830879 PMC4752902

[594] Zhang Q Tian K Xu J Zhang H Li L Fu Q. Synergistic effects of cabozantinib and EGFR-specific CAR-NK-92 cells in renal cell carcinoma. J Immunol Res. (2017) 2017:6915912. 10.1155/2017/6915912 29423418 PMC5750507

[595] Kwilas AR Ardiani A Donahue RN Aftab DT Hodge JW. Dual effects of a targeted small-molecule inhibitor (cabozantinib) on immune-mediated killing of tumor cells and immune tumor microenvironment permissiveness when combined with a cancer vaccine. J Transl Med. (2014) 12:294. 10.1186/s12967-014-0294-y 25388653 PMC4236498

[596] Li H Ding J Lu M Liu H Miao Y Li L. CAIX-specific CAR-T cells and sunitinib show synergistic effects against metastatic renal cancer models. J Immunother. (2019) 43:16–28. 10.1097/CJI.0000000000000301 31574023

[597] Wu X Huang S. HER2-specific chimeric antigen receptor-engineered natural killer cells combined with apatinib for the treatment of gastric cancer. Bull Cancer. (2019) 106:946–58. 10.1016/j.bulcan.2019.03.012 31711572

[598] PeRNA F Berman SH Soni RK Mansilla-Soto J Eyquem J Hamieh M. Integrating proteomics and transcriptomics for systematic combinatorial chimeric antigen receptor therapy of AML. Cancer Cell. (2017) 32:506–19.e5. 10.1016/j.ccell.2017.09.004 29017060 PMC7025434

[599] Short S Fielder E Miwa S von Zglinicki T. Senolytics and senostatics as adjuvant tumour therapy. EBioMedicine. (2019) 41:683–92. 10.1016/j.ebiom.2019.01.056 30737084 PMC6441870

